# Evolutionary route of nasopharyngeal carcinoma metastasis and its clinical significance

**DOI:** 10.1038/s41467-023-35995-2

**Published:** 2023-02-04

**Authors:** Mei Lin, Xiao-Long Zhang, Rui You, You-Ping Liu, Hong-Min Cai, Li-Zhi Liu, Xue-Fei Liu, Xiong Zou, Yu-Long Xie, Ru-Hai Zou, Yi-Nuan Zhang, Rui Sun, Wei-Yi Feng, Hai-Yan Wang, Gui-Hua Tao, Hao-Jiang Li, Wen-Jie Huang, Chao Zhang, Pei-Yu Huang, Jin Wang, Qi Zhao, Qi Yang, Hong-Wan Zhang, Ting Liu, Hui-Feng Li, Xiao-Bing Jiang, Jun Tang, Yang-Kui Gu, Tao Yu, Zhi-Qiang Wang, Lin Feng, Tie-Bang Kang, Zhi-Xiang Zuo, Ming-Yuan Chen

**Affiliations:** 1 Department of Nasopharyngeal Carcinoma, Sun Yat-sen University Cancer Center, 651 Dongfeng East Road, Guangzhou, 510060 P. R. China; 2 Department of Radiation Oncology, The First Affiliated Hospital of Sun Yat-Sen University, Zhongshan 2nd Road, Guangzhou, 510080 P. R. China; 3 Sun Yat-sen University Cancer Center, State Key Laboratory of Oncology in South China, Collaborative Innovation Center for Cancer Medicine, Guangzhou, P. R. China; 4 Guangdong Key Laboratory of Nasopharyngeal Carcinoma Diagnosis and Therapy, Guangzhou, 510060 China; 5 Take2 Health (Shenzhen) Limited, Shenzhen, 518066 P. R. China; 6 School of Computer Science and Engineering, South China University of Technology, 382 East Waihuan Road, Guangzhou, 510006 P. R. China; 7 Imaging Diagnosis and Interventional Center, Sun Yat-sen University Cancer Center, 651 Dongfeng East Road, Guangzhou, 510060 P. R. China; 8 Department of Ultrasound, Sun Yat-sen University Cancer Center, 651 Dongfeng East Road, Guangzhou, 510060 P. R. China; 9 College of Mathematics and Informatics, South China Agricultural University, 483 Wushan Road, Guangzhou, 510642 P. R. China; 10 Department of Pathology, Sun Yat-sen University Cancer Center, 651 Dongfeng East Road, Guangzhou, 510060 P. R. China; 11 Department of Musculoskeletal Oncology, Sun Yat-sen University Cancer Center, 651 Dongfeng East Road, Guangzhou, 510060 P. R. China; 12 Department of Neurosurgery, Sun Yat-sen University Cancer Center, 651 Dongfeng East Road, Guangzhou, 510060 P. R. China; 13 Department of Breast Oncology, Sun Yat-sen University Cancer Center, 51 Dongfeng East Road, Guangzhou, 510060 P. R. China; 14 Department of Minimally Invasive Interventional Radiology, Sun Yat-sen University Cancer Center, 51 Dongfeng East Road, Guangzhou, 510060 P. R. China

**Keywords:** Cancer genomics, Head and neck cancer, Metastasis, Tumour heterogeneity, Cancer therapeutic resistance

## Abstract

It is critical to understand factors associated with nasopharyngeal carcinoma (NPC) metastasis. To track the evolutionary route of metastasis, here we perform an integrative genomic analysis of 163 matched blood and primary, regional lymph node metastasis and distant metastasis tumour samples, combined with single-cell RNA-seq on 11 samples from two patients. The mutation burden, gene mutation frequency, mutation signature, and copy number frequency are similar between metastatic tumours and primary and regional lymph node tumours. There are two distinct evolutionary routes of metastasis, including metastases evolved from regional lymph nodes (lymphatic route, 61.5%, 8/13) and from primary tumours (hematogenous route, 38.5%, 5/13). The hematogenous route is characterised by higher IFN-γ response gene expression and a higher fraction of exhausted CD8^+^ T cells. Based on a radiomics model, we find that the hematogenous group has significantly better progression-free survival and PD-1 immunotherapy response, while the lymphatic group has a better response to locoregional radiotherapy.

## Abstract (web summary)

It is critical to understand the factors that are associated with nasopharyngeal carcinoma (NPC) progression, metastasis and response to therapy. Here, the authors analyse primary and metastatic NPC samples using bulk and single-cell sequencing, and find two distinct evolutionary routes that lead to metastasis.

## Introduction

Nasopharyngeal carcinoma (NPC) originates from the nasopharyngeal epithelium and is most prevalent in southeastern Asia, with the highest incidence reported among the Cantonese population from Guangdong, China^1^. The incidence of synchronous distant metastasis in endemic NPC ranges from 6% to 8% at the time of presentation^2^. Local therapy has been used for metastatic disease with the intent of reducing the primary tumour burden, propagating metastases, or relieving symptoms^3^. Our previous clinical trial demonstrated that chemotherapy plus high-dose locoregional radiotherapy could improve overall survival (OS) in de novo metastatic NPC patients^4^. As soon as the promising results of the clinical trial were published, the well-known National Comprehensive Cancer Network (NCCN) guidelines recommended systemic therapy followed by radiotherapy for de novo metastatic NPC patients (category 2 A)^5^. Furthermore, addition to the expected reduction in the locoregional relapses rate, locoregional radiotherapy also resulted in fewer distant metastatic recurrences (54.0% vs. 68.3%). This raises the question about the mechanisms linking the treatment of the primary tumour to effects on the metastatic disease trajectory. However, 17 of 63 (27.0%) patients did not achieve an objective response (complete or partial response) after the completion of locoregional radiotherapy. Therefore, it is critical to unveil the mechanisms contributing to this synergy observed in the clinic and to screen appropriate patients who could benefit from intense combined locoregional radiotherapy.

Large-scale genomic and transcriptomic sequencing studies have revealed a comprehensive mutation landscape and diverse disease subtypes in NPC samples^6–11^. However, the genomic architectures of metastases have not been defined to discern temporal or spatial patterns of metastatic evolution. In addition, single-cell RNA sequencing (scRNA-seq), a high-resolution technology, could provide new insights into tumour evolution and has been successfully used to reconstruct clonal evolutionary trajectories during chemotherapy in breast cancer^12^. scRNA-seq could also be used to dissect the intratumour heterogeneity (ITH) of primary and metastatic tumour ecosystems in NPC^13^ and draw a map of the immune phenotype in NPC^14–22^. Studies that focuses on the evolutionary route of metastasis in NPC are still lacking.

In this work, we collected paired primary and metastatic tumour samples from a previous trial (NCT02111460) in de novo metastatic NPC and conducted integrative genomic and transcriptomic sequencing, including scRNA-seq, to trace the evolutionary history of distant metastases in NPC. We reasoned that through integrative analysis of the large-scale bulk and single-cell sequencing data of paired primary and metastatic NPC tumours, a finer understanding of the evolutionary history of NPC metastases and the mechanism underpinning the effect of locoregional treatment on metastatic NPC could be obtained. Subsequently, we attempted to identify biomarkers to predict patient outcomes and aid in therapeutic decision-making and validate them in clinical trial cohorts.

## Results

### Genomic comparison of matched NPC primary, regional lymph node and metastatic tumours

**Fig. 1:**
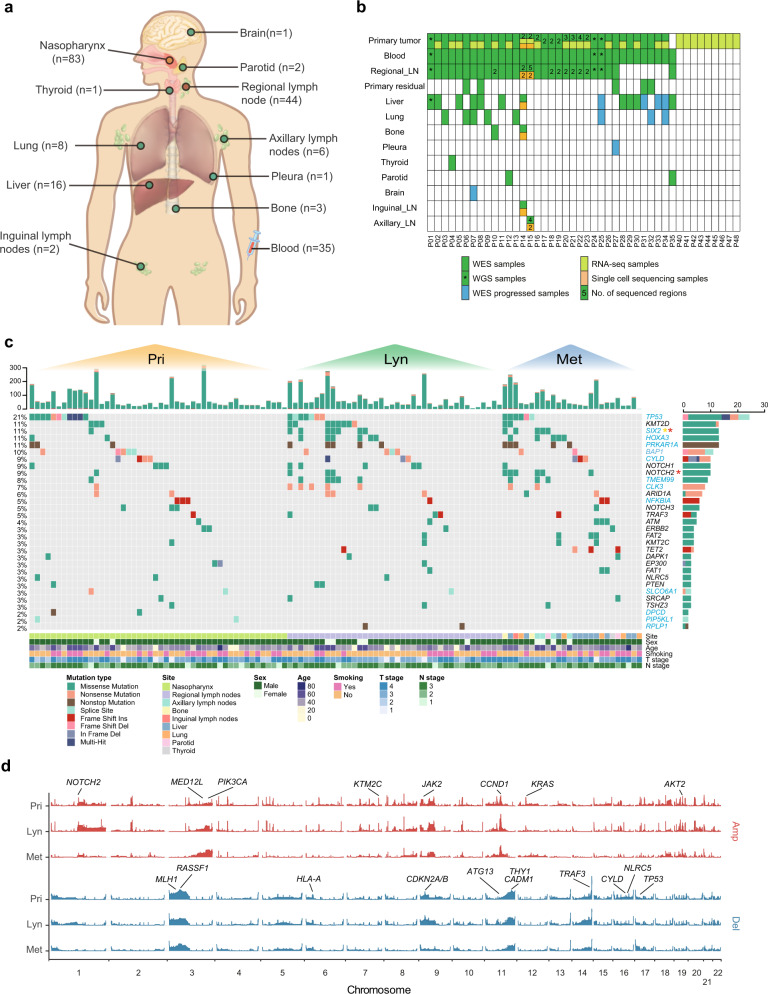
Genomic landscape of NPC primary and metastatic tumours. **a** Site distribution of the sequenced samples. The number of samples from each site is shown in brackets. **b** The sequencing strategy across all samples. **c** An overview of somatic mutations detected in primary tumour, regional lymph node metastasis and distant metastasis samples. The top panel shows the total mutations for each sample. The middle panel shows the mutation details for putative driver genes. The significantly mutated genes evaluated by MutSigCV are shown in blue, and the genes with significantly different frequencies in primary tumours and regional lymph node or distant metastasis are marked with asterisks (Pri vs. Lyn in yellow and Pri vs. Met in red). Clinical information is shown in the bottom panel. **d** The CNV landscape of primary tumour, regional lymph node metastasis and distant metastasis samples. The height of each bar refers to the G-score. Important frequently altered genes are highlighted. Source data are provided as a Source Data file.

First, to investigate the evolutionary routes of NPC metastases, we obtained 167 tumour samples (83 primary lesions, 44 regional lymph node lesions, and 40 metastatic lesions) and 35 matched blood samples from 44 NPC patients (Fig. 1a; Supplementary Table 1). Whole-exome/whole-genome sequencing (WES/WGS) was performed on 163 samples from 35 patients (400.97× on WES, and 89.11× on WGS) (Fig. 1b; Supplementary Data 1), among which 14 tumour samples were obtained after standard treatment (five samples were from residual primary lesions that were sampled when locoregional radiotherapy was completed, and nine samples were from progressed distant metastases tumours). To further investigate ITH, WES was performed on two to five multiregion samples from the primary tumour lesions of ten patients and the regional lymph node lesions of nine patients. Transcriptome sequencing (RNA-seq) was also performed on 28 patients with available primary tumour tissues (Fig. 1b; Supplementary Data 2). Moreover, high-resolution 10× genomics 3’ v2 scRNA-seq was performed on 11 samples from two patients (including two primary tumours, four regional lymph node tumours and five distant metastatic tumours) to explore the evolutionary routes of NPC metastases at the cellular resolution level (Fig. 1b; Supplementary Table 2).

On average, 70 (range from 6 to 326) non-silent somatic mutations were identified (Supplementary Data 3). Validation of candidate mutations with Sanger sequencing and TA vector clones showed that the true positive rate was 93.9% (Supplementary Data 4). The metastatic tumour samples had a non-silent mutation burden similar to that of primary and regional lymph node tumours (Supplementary Fig. 1a). The predominant type of substitution in primary, regional lymph node and metastatic tumours was C > T transition which was also the predominant signature reported in different NPC pathologic subtypes^11,23^ (Supplementary Fig. 1b). Using combined nonnegative matrix factorization clustering, we identified five robust mutational signatures across all samples (Supplementary Fig. 1c–e). The five mutation signatures were annotated to curated mutational signatures 2, 4, 5, 6 and 13 in line with the Catalogue of Somatic Mutations in Cancer (COSMIC) database. We then deconvoluted the mutation signatures for each sample according to the five mutation signatures plus signature 1, as it is universally found in almost all cancer types and in most tumour samples^24,25^. Overall, different samples had different dominant mutational signatures, but we also found that the AID/APOBEC-related signatures, including Signature 13 and Signature 2, were the top two contributing signatures across all samples, and the overall pattern of the mutation spectrum in metastatic tumour samples was similar to that of primary and regional lymph node tumour samples (Supplementary Fig. 1f; Supplementary Fig. 2a). Moreover, when longitudinally comparing the number of mutations, proportion of mutational signatures and proportion of subclone mutations across different patients, we found that these characteristics showed significant positive correlations in primary vs. regional lymph node tumours, primary vs. distant metastatic tumours, and regional lymph node vs. distant metastatic tumours (Supplementary Fig. 2b–d). Interestingly, the defective DNA mismatch repair signature (signature 6), which plays an essential role in maintaining genomic stability^26^ was predominant at all three sites. Consistently, previous studies have found that the DNA mismatch repair signature was dominant and associated with inferior survival^27^. Collectively, the dominance of signature 6 in metastatic tumours further indicated that defective DNA mismatch repair might exert a broad and important influence on the progression of NPC (Supplementary Fig. 1d, e).

To evaluate the ITH in NPC tumours, we reconstructed phylogenetic trees based on multiregion primary tumour samples from ten NPC patients and multiregion regional lymph node tumour samples from nine patients (Supplementary Fig. 3a, b). On average, only 20.79% (range from 4.80% to 38.28%) and 22.29% (range from 2.45% to 38.28%) of mutations were presented as trunk mutations that were shared by all regions in primary and regional lymph node tumours, respectively, suggesting that substantial high ITH exists in primary and regional lymph node tumours. Moreover, by classifying somatic variants into clonal and subclonal mutations according to the cancer cell fraction (CCF), we found that the proportion of subclonal mutations in metastatic tumours was comparable to that in primary and regional lymph nodes (Supplementary Fig. 3c). Furthermore, the proportion of subclonal mutations in primary tumours was positively correlated with that in regional lymph node/distant metastasis tumours (R = 0.5, *P* < 0.001; Supplementary Fig. 3d). Concordantly, the MATH score^28^ was also adopted to evaluate the ITH of tumours, which showed that ITH was similar in primary, regional lymph node and metastatic tumours (Supplementary Fig. 3e, f). All these results suggested that NPC possessed high ITH, and regional lymph node and metastatic tumours might inherit this characteristic.

We then applied MutSigCV^29^ to separately identify significantly mutated genes (SMGs) in primary, regional lymph node and distant metastatic tumours. For tissues with multiregion samples, we combined their mutation results for the above analysis to avoid the influence of the number of samples. In addition to genes that were reported to be frequently mutated in previous genomic studies of NPC, such as *TP53*, *BAP1*, *CYLD* and *NFKBIA*, we also identified other SMGs, such as *SIX2* and *RPLP1* (Fig. 1c). A combined list of 31 “driver genes” was generated, which consisted of the SMGs we identified, the genes identified as key genes in previous NPC studies^6–8^ and the frequently mutated genes (>3%) in our samples (Fig. 1c). While most driver genes had highly similar mutational frequencies across the three sites, the mutational frequencies of *SIX2* and *NOTCH2* were significantly higher in metastatic tumour samples than in primary tumour samples (Fig. 1c), suggesting that *SIX2* and *NOTCH2* might play an important role in NPC metastasis. Recent studies have reported that *SIX2*, a developmental transcription factor, promotes breast cancer metastasis via the epithelial-mesenchymal transition (EMT) pathway or the induction of the cancer stem cell program^30,31^. *NOTCH2*, a newly identified oncogene that is commonly overexpressed in a range of cancers^32^, can promote cancer growth and metastasis in bladder cancer^33^.

We derived somatic copy number alterations (SCNAs) using WES segmentation data via the GISTIC algorithm^34^ (Supplementary Data 5). We obtained “amplification (AMP)” and “deletion (DEL)” alterations at the gene level based on the “high-level amplification (or deletion) thresholds of segment mean” provided by GISTIC2 (Fig. 1d). No significantly differential SCNAs were detected in regional lymph node or distant metastasis tumours when compared to primary tumours. Previously reported well-known copy number deletions at 9p21.3 (*CDKN2A*), 3p21.31 (*RASSF1*), 3p22.2 (*MLH1*), and 14q32.32 (*TRAF3*) and amplifications at 11q13.3 (*CCND1*), 3q26.1 (*PIK3CA*), 1p12 (*NOTCH2*), and 19q13.2 (*AKT2*) were prevalent at all three sites. Although high ITH was found in NPC regardless of the site, the basic genomic characteristics were similar among primary, regional lymph node and distant metastatic tumours.

### Identification and characterization of metastatic driver events

**Fig. 2:**
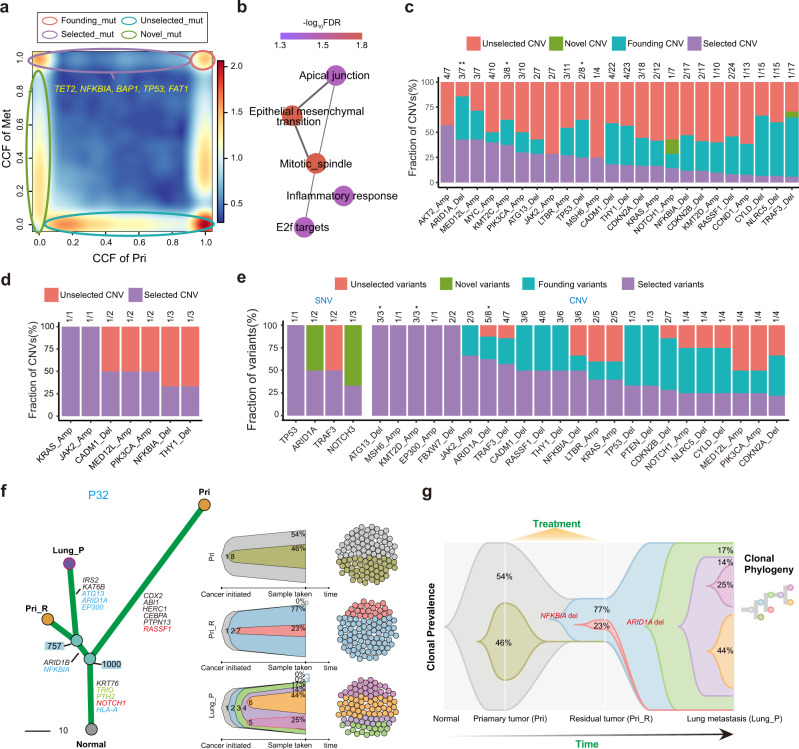
Driver events during NPC metastasis. **a** Scatter plot indicating different variant groups during metastasis. Each variant is measured by CCFs in the primary tumour and distant metastasis from the same patient. The colour range reflects the mutation density. Red circles, founding variants that are clonal both before and after metastasis; purple circles, selected variants that are undetectable or subclonal before metastasis but clonal after metastasis; blue circles, unselected variants that are clonal or subclonal before metastasis and undetectable after metastasis; and green circles, novel variants that are subclonal after metastasis but undetectable before metastasis. Predicted driver genes harbouring SSNVs are marked. **b** Reactome pathway enrichment of mutated genes in the “selected” group. **c** Distribution of variant groups during metastasis according to key NPC CNVs. Only selected CNVs are shown, and the number of selected CNVs was annotated. **d** Distribution of variant groups during progression according to the key CNVs between paired primary tumours and residual tumour after treatment. The number of selected variants is annotated. **e** Distribution of variant groups during progression according to key variants, including SSNVs and CNVs, between paired primary tumour samples and posttreatment progressed metastatic samples. **f** The evolutionary landscape of P32. The left panel shows the phylogenetic tree with the key variants highlighted. The right panel shows the subclone-based evolution architecture of each sample and their sampling transect, which reflects the proportion of each subclone at the time of sampling. **g** Dynamic subclone-based evolution architecture of P32 plotted using TimeScape, with the proportion of each cluster at the time of sampling annotated. Source data are provided as a Source Data file.

Tumour metastasis is recognised as a clonal evolution process, during which a clone equipped with metastatic capability is selected for dissemination to metastatic sites^35^. To investigate the driver events selected during NPC metastases, we systematically characterised the clonality of each variant and the tumour subclonal architectures of metastatic NPC based on the CCFs of variants (Supplementary Fig. 11; Methods). In total, 11,148 variants were categorised into four groups during metastasis according to the dynamic change in clonality between primary and metastatic tumours: 1) selected variants (“selected”, *n* = 1193 variants, defined as those that were clonal in metastatic tumours but subclonal or absent in primary tumours); 2) novel variants (“novel”, *n* = 3603 variants, defined as those that were subclonal in metastatic tumours but absent in primary tumours; 3) founding variants (“founding”, *n* = 718 variants, defined as those that were clonal in both metastatic and primary tumours; and 4) unselected variants (“unselected”, *n* = 5634 variants, defined as those that were not found in metastatic tumours but were present in primary tumours regardless of clonality) (Fig. 2a).

We found that clones harbouring somatic mutations in driver genes such as *NFKBIA*, *TET2*, *BAP1*, *TP53 and FAT1* were evidently selected in metastasis (Fig. 2a). Furthermore, we found that the somatic mutations in the selected group were significantly enriched in the EMT, mitotic spindle, apical junction, inflammatory response and E2F target pathways (Fig. 2b). These pathways have been widely reported as potential drivers of cancer metastasis^36–39^. Similarly, we found that clones harbouring CNV events, such as *KMT2C amplification*, *ARID1A deletion*, *and TP53 deletion*, were evidently selected in metastasis (Fig. 2c).

It has been reported that approximately 20%–30% of NPC patients experience distant metastasis after standard chemoradiotherapy^40,41^. Benefiting from the precious paired tumour samples collected after treatment, we sought to further investigate the influence of treatment on the mutation selection and tumour evolution of NPC metastasis. Obviously, intense treatments impose selective pressure on variants; thus, clonal variants persistent in post-treatment metastatic tumours might be relevant to not only treatment resistance but also tumour metastasis. First, we found that some events, such as *KRAS amplification*, *JAK2 amplification*, *CADM1 deletion*, and *NFKBIA deletion*, were clonal in residual primary tumours but subclonal or undetectable in pretreatment primary tumours, suggesting that these events might be associated with treatment resistance and further metastatic progression (Fig. 2d). In line with our findings, *KRAS* is known to confer chemoresistance in various cancer types^42–44^. *NFKBIA*, a well-known negative regulator of the NF-κB pathway, was reported to enhance chemoresistance in NPC^45–47^. Second, by comparing the mutation CCF between the paired pretreatment primary and posttreatment progressed metastasis samples, we found that SNVs in genes such as *TP53*, *ARID1A* and *TRAF3* and CNVs such as *ATG13 deletion*, *KMT2D amplification* and *ARID1A deletion* were evidently selected in posttreatment progressed metastatic tumours (Fig. 2e). Most selected CNVs in posttreatment metastatic tumours were also found to be selected in pretreatment metastatic samples, except *EP300 amplification*, *FBXW7 deletion* and *PTEN deletion* (Fig. 2c and e). *EP300*, an oncogene found in oesophageal squamous carcinoma^48^, could promote tumour progression in diffuse large B-cell lymphoma by altering tumour-associated macrophage polarization via downregulation of *FBXW7*^49^, a critical tumour suppressor deleted in more than 30% of all human cancers^50^. *EP300 amplification* and *FBXW7 deletion* might exert a synergistic effect on the progression of metastasis in NPC. In addition, all selected variants found in residual primary tumours were also observed to be selected in both pretreatment and posttreatment metastatic samples, which proved that such variants were not brought about by treatment and probably played a vital role in initiating distant metastasis.

Since the collection of posttreatment primary residual samples was scarce but valuable in our study, we further inspected the topology of phylogenetic trees of patients with posttreatment primary residual samples (P27, P31 and P32). According to the phylogenetic trees of P27 and P31 (Supplementary Fig. 11), we found that the progressed metastasis sample and the primary sample before treatment were clustered into the same clade, suggesting that the posttreatment progressed metastatic lesions were related to the primary lesions before treatment and might have originated from the clone of the primary lesions before treatment. In contrast, the progressed metastasis sample and the posttreatment primary residual sample were clustered into the same clade in P32, indicating that the progressed metastasis might have originated from the clone of the posttreatment primary residual lesions (Fig. 2f). According to the phylogenetic tree of P32, we found that clonal *NFKBIA deletion* was present in both the progressed metastasis sample and the posttreatment residual primary sample but absent in the pretreatment primary tumour (Fig. 2g), suggesting that *NKFBIA deletion* might have conferred treatment resistance and further triggered distant metastasis in P32. Moreover, we found that clonal *ARID1A deletion* was detected in only the progressed metastasis (Fig. 2g), suggesting that *ARID1A deletion* was important for the metastatic progression of P32. All these inferences need further validation and exploration in large cohorts in the future.

### Two distinct routes of metastasis evolution revealed by the genomic data of matched NPC primary, regional lymph node and metastatic tumours

**Fig. 3:**
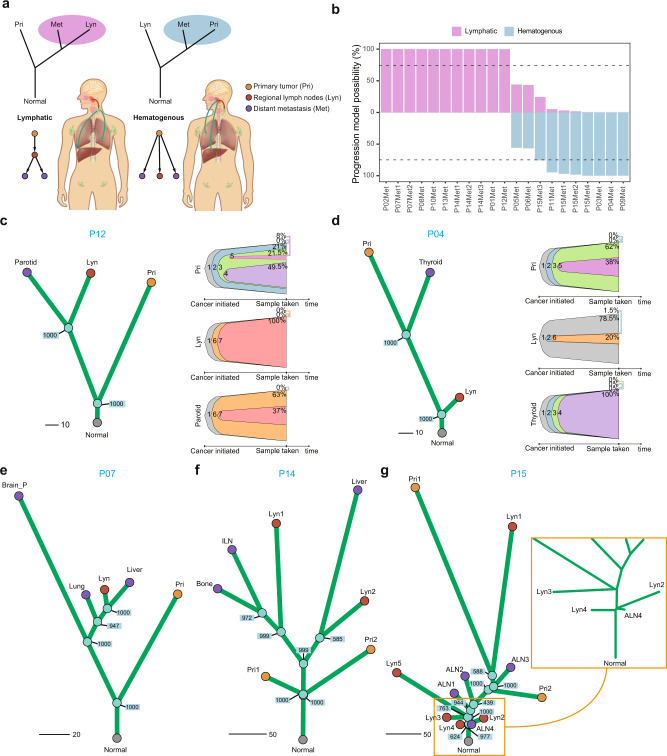
“Lymphatic” and “hematogenous” evolution pattern of NPC metastases indicated from genome sequencing data. **a** Schematic diagram illustrating the classification of distant metastases into lymphatic or hematogenous evolution patterns. **b** Bootstrap values reflecting the evolutionary pattern confidence for each distant metastasis in 15 patients with matched primary, regional lymph node metastasis and distant metastasis samples before treatment. Samples with both probabilities less than 75% (P05Met, P06Met), as indicated by the dashed lines, were too ambiguous for classification and were removed in downstream analysis. **c**, **d** Typical examples of lymphatic (**c**) and hematogenous (**d**) models. Left: Phylogenetic tree with bootstrap values for each divergence node. Right: Subclone-based evolution analysis revealed the same pattern as the phylogenetic tree. Each colour of the bell plot represents a specific subclone. For the lymphatic pattern, one or more subclones found in the distant metastasis could also be found in the regional lymph node metastasis instead of the primary tumour. Otherwise, this kind of cluster is not found in the hematogenous evolution pattern. The CCF value of each subclone in each sample at the time of sampling is marked in the bell plot. **e**–**g** Distant metastases show consistent evolutionary patterns revealed by patients with multiregion or multiorgan distant metastases. All distant metastases from P07 and P14 are classified as the lymphatic route (**e**, **f**), and all distant metastases from P15 are classified as the hematogenous route (**g**). Source data are provided as a Source Data file.

To elucidate the evolutionary routes of NPC metastases, we examined all the phylogenetic trees of the 15 patients with complete matched WES data of primary, regional lymph node and metastatic tumours (Supplementary Fig. 11; Methods). According to the topology of the phylogenetic tree, we differentiated two evolutionary routes of metastases: (1) lymphatic route: distant metastases were seeded from regional lymph node lesions, where regional lymph node tumour and distant metastatic tumour were clustered into the same clade without a primary tumour; and (2) hematogenous route: distant metastases were directly seeded from primary tumours, where primary tumour and distant metastatic tumour were clustered into the same clade bypassing regional lymph node tumours (Fig. 3a). We employed a bootstrapping strategy to assess the probability of lymphatic and hematogenous origination of each metastasis (Methods). Filtering two metastatic tumours from two patients (P05 and P06) that did not meet the cut-off (0.75) of probability, we observed that 11 metastatic tumours from eight patients (P01, P02, P07, P08, P10, P12, P13 and P14; 8/13, 61.5%) were seeded via the lymphatic route, and eight metastatic tumours from five patients (P03, P04, P09, P11 and P15; 5/13, 38.5%) were most likely seeded via the hematogenous route (Fig. 3b; Supplementary Fig. 11).

Mutation CCF-based subclonal evolution analysis further confirmed our findings (Methods). Metastatic tumours taking the lymphatic route would share at least one private subclone with regional lymph node tumours, while metastatic tumours taking the hematogenous route would not share any private subclone with regional lymph node tumours (Supplementary Fig. 11). For instance, the parotid metastatic tumour of P12 originated from the regional lymph node tumour according to the mutation-based phylogenetic tree. This was confirmed by the subclonal architecture derived from the CCF of mutations, showing that the parotid metastasis and the regional lymph node of P12 shared the private subclones “6” and “7” that were not found in the primary tumour (Fig. 3c). In contrast, the evolutionary model of P04’s metastatic tumour (thyroid) hematogenously originated from the primary tumour. The distant metastatic and regional lymph node tumours of P04 were not clustered into the same clade of the phylogenetic tree and did not share any private subclones (Fig. 3d). Overall, the evolutionary routes of 89.47% of distant metastatic lesions were confirmed by subclone-based evolutionary analysis, except P14-Met3 (subclones that uniquely shared by Lyn and Met are undetectable) and P01-Met (subclone-based evolution architecture unavailable) (Supplementary Fig. 11).

Interestingly, we found that different metastatic tumours from the same patient tended to obey the same evolutionary route. In other words, the lymphatic route and hematogenous route might be exclusive. The liver and lung metastases in P07 evolved from regional lymph nodes, and all metastatic tumours (liver, bone and inguinal lymph node) of P14 also evolved from regional lymph node tumours (Fig. 3e, f; Supplementary Fig. 11). Similarly, all distant metastatic tumours (multiple axillary lymph nodes) of P15 hematogenously originated from the primary tumour (Fig. 3g; Supplementary Fig. 11). As the classification of metastatic routes is mainly based on the phylogenetic tree, omission of tumour subclones due to single-region biopsy might give rise to false classification. However, since surgery is not recommended for NPC patients, it is difficult to exhaust tumour cells. Thus, comprehensively evaluating metastases through 18F-fluorodeoxyglucose positron emission tomography-computed tomography (18F-FDG PET-CT) and other imaging examinations, we safely obtained as much tumour tissue as possible via biopsy and obtained multi-region samples of large tumours in available patients (e.g., P10, P14 and P15; Supplementary Fig. 11). We found that different regions from the same primary tumour or the same regional lymph node tumour had consistent evolutionary routes (e.g. P10, P14, P15; Supplementary Fig. 11). To determine the influence of the number of samples biopsied on the categorisation of the metastatic routes, we removed one sample at a time, reconstructed the phylogenetic tree and reclassified the patients according to the two metastatic routes. No change in metastatic routes was found in P14 and P10 when one sample was randomly removed, while the classification of the metastatic routes of P15 was changed only when Lyn1 was removed. All these results suggested that biopsy of samples had limited influence on the determination of the evolutionary route of metastasis based on the phylogenetic tree and underlined the importance of multiregion sampling in such studies.

**Fig. 4:**
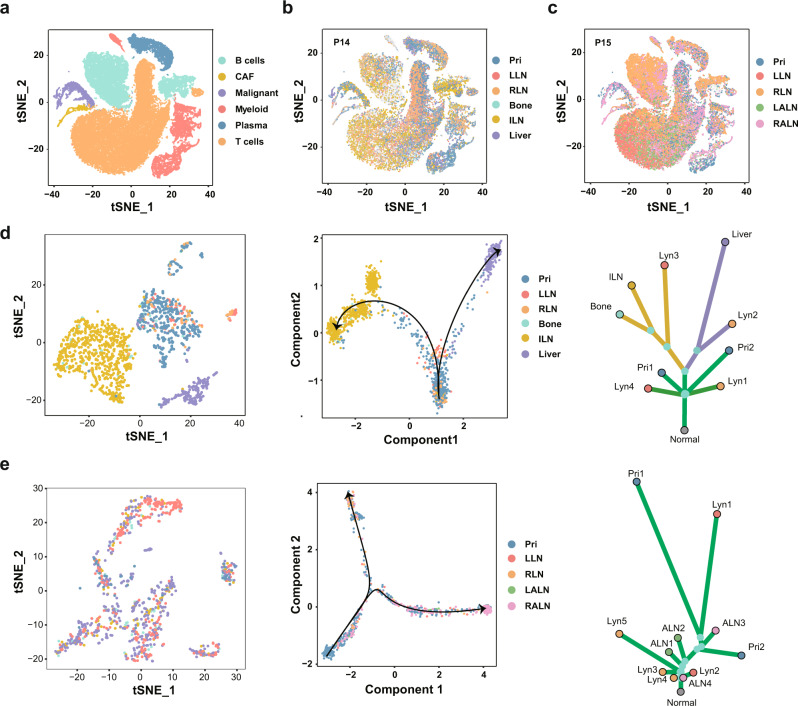
Lymphatic and hematogenous evolution models revealed by scRNA-seq. **a** The t-SNE plot showing the unsupervised clustering results with cell types annotated. **b** The t-SNE plot showing the sample locations of single cells from P14. **c** The t-SNE plot showing the sample locations of single cells from P15. **d**, **e** Pseudotime trajectory analysis of tumour cells from P14 (**d**) and P15 (**e**). The left panel shows the t-SNE plot of tumour cells coloured according to the sample locations, the middle panel shows the evolutionary trajectory of tumour cells, and the right panel shows the corresponding phylogenetic tree. Source data are provided as a Source Data file. Pri, LLN, RLN, ILN, LALN, and RALN: primary tumour, left regional lymph nodes, right regional lymph nodes, inguinal lymph nodes, left axillary lymph nodes and right axillary lymph nodes, respectively.

scRNA-seq could dissect tumour heterogeneity at the cellular level and subsequently add additional resolution to tumour evolution^51^, thus we further performed scRNA-seq in matched primary, regional lymph node and distant metastatic tumours from lymphatic (P14) and hematogenous (P15) metastatic patients (Fig. 1b; Supplementary Table 2). After quality control and batch effect removal, 53,913 cells from 11 samples were detected (Fig. 4a–c; Supplementary Table 2). Malignant cells were identified using epithelial markers such as *EPCAM* and *KRT18*, and nonmalignant cells were annotated as myeloid immune cells, B cells, plasma cells, T cells and cancer-associated fibroblasts according to canonical markers (Fig. 4a–c; Supplementary Fig. 4a). Unsupervised clustering of malignant cells from P14 and P15 revealed nine and eight distinct clusters, respectively (Supplementary Fig. 4b, c). Mapping cells with sample origination, a consecutive tumour evolutionary trajectory was observed via Monocle2^52^ (Fig. 4d–e; Supplementary Fig. 4b, c). Intriguingly, primary tumour cells first migrated to regional lymph nodes and then subsequently disseminated to bone and inguinal lymph nodes or the liver following distinct routes, which was closely consistent with the lymphatic evolutionary route revealed by the phylogenetic tree built using the genomic data (Fig. 4d; Supplementary Fig. 4b). In contrast, malignant cells from primary and metastatic sites were blended and no clear evolutionary route related to sample originations was found in P15, probably due to the direct dissemination of tumour cells from the primary tumour to regional lymph nodes or distant metastasis revealed by the phylogenetic tree (Fig. 4e; Supplementary Fig. 4c). Overall, the scRNA-seq data not only confirmed the evolutionary route derived from the WES data but also provided a finer resolution to clarify NPC metastases.

### Molecular differences between lymphatic and hematogenous routes of NPC metastases

**Fig. 5:**
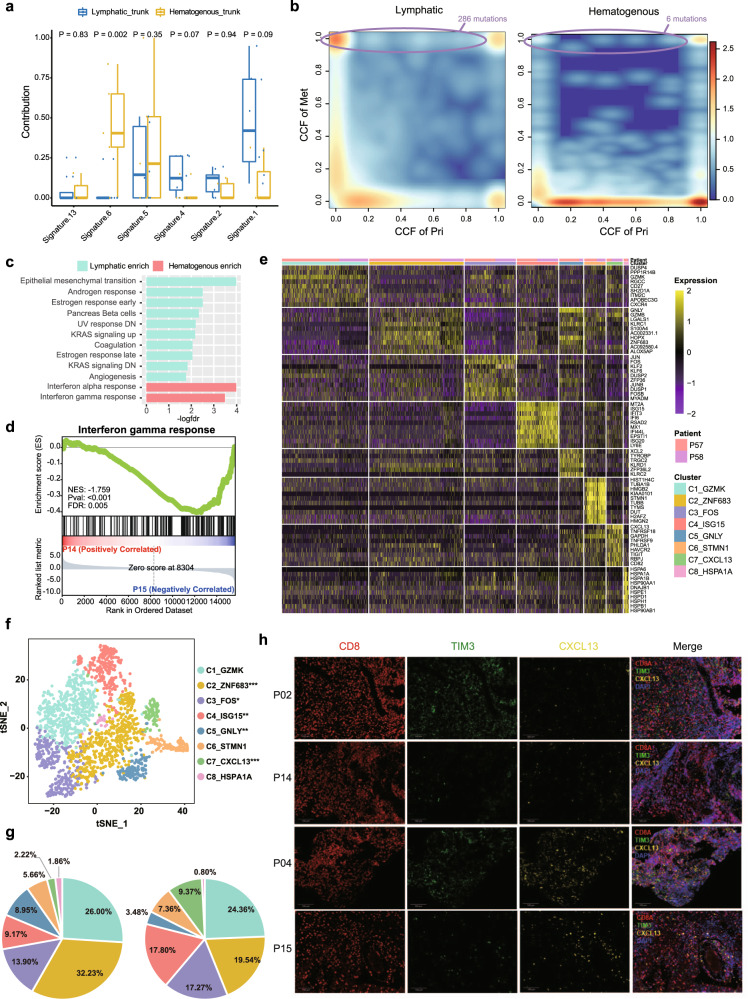
Molecular characteristics of the lymphatic and hematogenous evolutionary modes. **a** Mutation signature profile of trunk mutations in samples with the lymphatic pattern (*n* = 8) and the hematogenous pattern (*n* = 5). The y-axis shows the contribution of each signature. In each box plot, the centre line represents the median, the bounds represent the first and third quartiles, and whiskers extend from the hinge to the largest value no further than 1.5 × interquartile range (IQR) from the hinge. After adjusting for age, gender and tumour stage by using covariance analysis model, the Wilcoxon signed-rank test (two-sided) was used to calculate the *P* values. **b** Scatter plots showing the selection pattern of all mutations during metastasis in the lymphatic (left) and hematogenous (right) routes according to the comparison of the CCF for each variant between primary (x-axis) and metastatic (y-axis) tumours. The colour range reflects the mutation density in the scatter plot. The circle box indicates the selected mutations with the numbers of selected mutations marked. **c** Hallmark GSEA of lymphatic vs. hematogenous primary tumour using bulk RNA-seq data. **d** Hallmark GSEA of P14 primary tumour cells vs. P15 primary tumour cells using scRNA-seq data. An empirical phenotype-based permutation test (two-sided) was used to calculate the P value and false discovery rate (FDR). **e** Heatmap showing the differentially expressed genes among different CD8^+^ T-cell subclusters from primary tumours. Information on the clusters and patient-of-origin is shown at the top. **f** The t-SNE projection of CD8^+^ T cells from primary tumour, with cells coloured based on the unsupervised clustering results. The statistical significance of the difference in the proportion of each cell type between P14 and P15 was measured using Fisher’s exact test (two-sided) and is marked in the figure legend (“*”, *P* < 0.05; “**”, *P* < 0.01; “***”, *P* < 0.001). **g** Pie charts showing fractions of CD8^+^ T-cell subclusters for P14 (left) and P15 (right). **h** Infiltration of exhausted CD8^+^ T cells enriched in the microenvironment of the hematogenous route. Independent experiments were conducted in patients with clear metastatic route classification (*n* = 13). Representative multiplex immunohistochemistry (IHC) staining images of CXCL13^+^ TIM3^+^ CD8^+^ T cells in the lymphatic route (P02 & P14) and hematogenous route (P04 & P15). Scale bar, 100 μm. Source data are provided as a Source Data file.

Next, we investigated whether the two routes have distinct genomic features. We found that the trunk mutations in patients with metastases emerging via the lymphatic route displayed a significantly higher fraction of the “C > A” transition than those in patients with metastases emerging via the hematogenous route (*P* = 0.030, Wilcoxon signed-rank test) (Supplementary Fig. 5). Moreover, mutation signature 6 (DNA mismatch repair-related) was significantly enriched in the hematogenous route (*P* = 0.002, Wilcoxon signed-rank test; Fig. 5a). Interestingly, we found that metastatic tumours emerging via the lymphatic route had dramatically more selected mutations than those emerging via the hematogenous route (286 mutations vs. 6 mutations, *P* < 0.001, Fisher’s exact test), and mutations in *NFKBIA*, *TP53*, genes involved in EMT, such as *CTNNB1*, vinculin (*VCL*), and Rho GTPase activators (*ARHGAP35* and *VAV3*), were evidently selected during metastasis via the lymphatic route but not during metastasis via the hematogenous route (Fig. 5b).

Furthermore, the bulk RNA-seq data showed that primary tumours of the lymphatic route were significantly enriched in pathways such as EMT (NES = 2.1, FDR < 0.001), UV response (NES = 1.7, FDR = 0.006) and angiogenesis (NES = 1.5, FDR = 0.001), while primary tumours of the hematogenous route were significantly enriched in the interferon-alpha (IFN-α) (NES = 2.4, FDR < 0.001) and interferon-gamma (IFN-γ) (NES = 1.9, FDR < 0.001) response pathways (Fig. 5c). The scRNA-seq data also showed that the IFN-α and IFN-γ response pathways were significantly enriched in primary tumour cells of the hematogenous route (Fig. 5d; Supplementary Fig. 4d). Previous studies have shown that IFN-γ upregulates the checkpoint inhibitor PD-L1 and cooperates with PD-1 to exhaust T cells, thus suppressing the antitumour immune response^53,54^. Concordantly, we observed that the proportion of PD-L1^+^ primary tumour cells was significantly higher in P15 (hematogenous) than P14 (lymphatic) (Supplementary Fig. 6a; chi-square test, *P* < 0.001). Based on the bulk RNA-seq data, we also found that the expression level of *PD-L1* tended to be higher in the hematogenous group than in the lymphatic group, although the difference was not statistically significant, probably due to the small sample size (Supplementary Fig. 6b, Wilcoxon rank test, *P* = 0.43). We hypothesise that the immune microenvironment of primary tumour of the hematogenous route should be present with more exhausted T cells, which is convenient for tumour cell dissemination. Thus, we examined and reclustered the immune cells derived from the primary tumour of P14 and P15. According to specific genes of different immune cell types (Supplementary Fig. 6c), we observed distinct clusters for B cells, CD4^+^ T cells, CD8^+^ T cells, macrophages and dendritic cells (Supplementary Fig. 6d). We found that the tumour immune microenvironments of P14 (lymphatic route) and P15 (hematogenous route) were significantly different. The fraction of CD8^+^ T cells, the main defender against tumour cells, was significantly higher in P14 (lymphatic route) (*P* < 0.001, Fisher’s exact test; Supplementary Fig. 6d, e). CD8^+^ T cells were further clustered into eight subclusters, and markers of each subcluster were extracted (Fig. 5e, f). Indeed, we observed that C7_CXCL13 with reduced cytotoxicity highly expressed exhausted markers such as *ENTPD1*, *TNFRSF9* and *TNFRSF18* and was significantly abundant in P15 (hematogenous route) (*P* < 0.001, Fisher’s exact test; Fig. 5g; Supplementary Fig. 6f, g). We further validated the enrichment of C7_CXCL13 cells in CD8^+^ T cells of hematogenous route samples via multiplex immunohistochemistry (IHC) staining of markers (CXCL13, TIM3 and CD8), which showed a consistent result with our single-cell data, although the results failed to reach statistical significance, probably due to the small sample size (Fig. 5h; Supplementary Fig. 6h). As immune checkpoint inhibitors (ICIs) could reinvigorate exhausted T cells, it is rationale to suppose that the enrichment of C7_CXCL13 might indicate a good response to immunotherapy. To determine whether the enrichment of exhausted CD8^+^ T cells with high expression of *CXCL13* is correlated with better efficacy of immune checkpoint blockade (ICB), we collected two public scRNA-seq datasets with ICB response information^55,56^ and found that exhausted CD8^+^ T cells were more prevalent in patients sensitive to ICB than in those resistant to ICB in bladder cell carcinoma (BCC) and clear cell renal cell carcinoma (ccRCC) (Supplementary Fig. 7). These clues suggest that NPC patients with metastases emerging via the hematogenous route might achieve a good response to immunotherapy.

### Imaging data discriminated the two metastatic routes

**Fig. 6:**
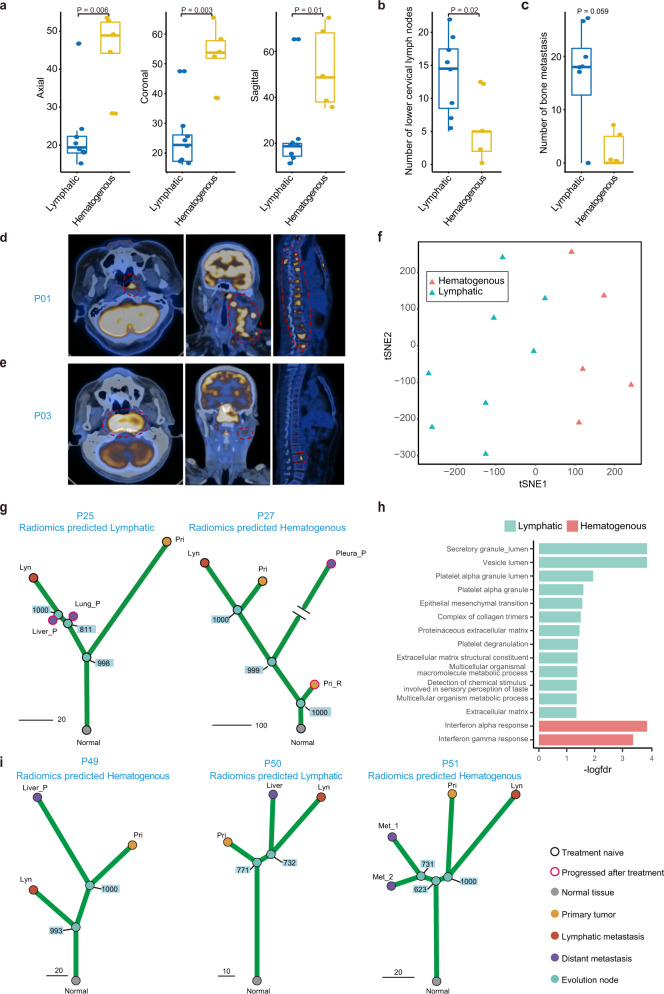
Imaging characteristics distinguished the lymphatic metastatic pattern from the hematogenous metastatic pattern. **a** Box plot shows the longest diameter of the primary tumour in the axial (left), coronal (middle) and sagittal views (right) in the lymphatic (*n* = 8) and the hematogenous (*n* = 5) models. In each box plot, the centre line represents the median, the bounds represent the first and third quartiles and whiskers extend from the hinge to the largest value no further than 1.5 × interquartile range (IQR) from the hinge. The Wilcoxon signed-rank test (two-sided) was used to calculate the P values; “*”*P* < 0.05. **b**, **c** Box plot shows the number of lower cervical lymph nodes (**b**) and bone metastasis lesions (**c**) in the lymphatic (*n* = 8) and hematogenous (*n* = 5) models. In each box plot, the centre line represents the median, the bounds represent the first and third quartiles and whiskers extend from the hinge to the largest value no further than 1.5 * interquartile range (IQR) from the hinge. The Wilcoxon signed-rank test (two-sided) was used to calculate the *P* values. “*”*p* < 0.05. **d**, **e** Positron emission tomography-computed tomography (PET-CT) images of the primary tumour (left), regional lymph node metastasis (middle) and bone metastasis (right) in the lymphatic (**d**) and hematogenous (**e**) models. The red dashed circle indicates tumour location. **f** The t-SNE plot shows the unsupervised clustering results of radiomics features of patients in the lymphatic and hematogenous models. **g** Phylogenetic tree with the bootstrap value on each divergence node showed the evolutionary route of patients without complete matched primary, regional lymph node metastasis and distant metastasis samples before treatment. The radiomics prediction model classifies patients with metastatic tumour after treatment into the lymphatic (left) and hematogenous (right) groups. **h** GSEA of radiomics model-predicted lymphatic vs. hematogenous primary tumour using bulk RNA-seq data. **i** Radiomics prediction model results and phylogenetic trees for newly collected patients to further validate the radiomics prediction model. *P: progressed samples after treatment. Source data are provided as a Source Data file.

Imaging data have been recently utilised to help clinicians diagnose how body systems work together at the organ-tissue level^57^. We found that there were distinct radiomics features between NPC patients with metastases emerging via the lymphatic route and those with metastases emerging via the hematogenous route. Patients with metastases emerging via the hematogenous route tended to have larger primary lesions but less involvement of lower regions of regional lymph nodes and less metastatic lesions in bone (Fig. 6a–c, Supplementary Table 3), as illustrated in P01 with the lymphatic route (Fig. 6d) and P03 with the hematogenous route (Fig. 6e). Since performing genomic sequencing and analysis on matched primary, regional lymph node and metastases tumours to classify metastatic routes is complicated, high-cost and time consuming, we wondered whether radiomics features could be a proxy to identify different metastatic routes. Thus, we extracted the image features of primary tumours and then built a machine learning-based prediction model to identify the lymphatic route from the hematogenous route using the radiomics data of the 13 patients with genomic evidence as the training dataset; this model, obtained a high accuracy rate of 1.0 and a high area under the curve (AUC) of 1.0 (Fig. 6f; Supplementary Fig. 8; Supplementary Fig. 9a–c; Supplementary Table 1; Supplementary Data 6; Methods). Moreover, we applied the radiomics prediction model to patients who did not have complete paired primary, regional lymph node and metastatic tumour samples before treatment and thus, the metastatic route might not be able to be identified based on the available genomic data. For P25, the radiomics prediction model indicated that metastases might emerge via the lymphatic route based on the imaging data obtained before treatment. As expected, the two posttreatment metastatic samples (liver and lung metastases) did emerge via the lymphatic route according to the genomic-based phylogenetic tree (Fig. 6g). Similarly, the radiomics model and genomic evidence both indicated that P27, whose pretreatment metastatic tumour was unavailable but progressed pleural metastasis sample was obtained, had metastases via the hematogenous route (Fig. 6g). Moreover, the primary tumours of the hematogenous route predicted via the radiomics model had higher IFN-α (NES = 2.4, FDR < 0.001) and IFN-γ (NES = 2.2, FDR < 0.001) response pathway activities than those of the lymphatic route (Fig. 6h), which was consistent with previous results based on samples with genomic evidence.

It is hard to collect complete matched primary, regional lymph node and metastases tumour samples as most metastatic sites are deep seated and near pivotal structures like heart and important vessels, which makes biopsy difficult and unsafe. To validate the accuracy of the radiomics prediction model, we strived to obtain additional matched primary, regional lymph node and metastases tumours (P49-P53) and then constructed genomic phylogenetic trees and predicted the metastatic route using the radiomics prediction model synchronously, which showed that the phylogenetic trees based on genomic data were concordant with the results of the radiomics prediction (Fig. 6i; Supplementary Fig. 9d; Supplementary Fig. 10a). In addition, we observed that P19 and P23 developed metastases after curative chemoradiotherapy; thus, we collected the posttreatment metastatic sites and reconstructed the phylogenetic trees. Consistent with the results of the radiomics prediction model, P19 had metastases via the hematogenous route while P23 had metastases via the lymphatic route according to the reconstructed phylogenetic trees (Supplementary Fig. 10a). Then, we applied our radiomics prediction model in larger clinical cohorts, including the de novo metastatic NPC cohort (*n* = 104, NCT02111460; Supplementary Tables 4 and 5) and immunotherapy cohort (*n* = 66; Supplementary Table 7). We found that the imaging characteristics of the lymphatic and hematogenous routes in the clinical cohorts were concordant with those in the training dataset (Supplementary Table 3). Since most patients diagnosed in the clinic are in stage M0 and might eventually develop metastasis, it would be of great help to predict the metastatic route as early as possible and impose specific treatment modalities to prevent the occurrence of distant metastasis. Thus, we also applied the prediction model to an M0-stage NPC cohort (*n* = 201) and found that the size of the primary tumour was larger and the number of lower cervical lymph nodes was lower in the hematogenous group than in the lymphatic group (Supplementary Table 3; Supplementary Table 6). These results suggested that the radiomics prediction model could classify the lymphatic and hematogenous routes.

### Clinical differences between the lymphatic and hematogenous metastatic routes

**Fig. 7:**
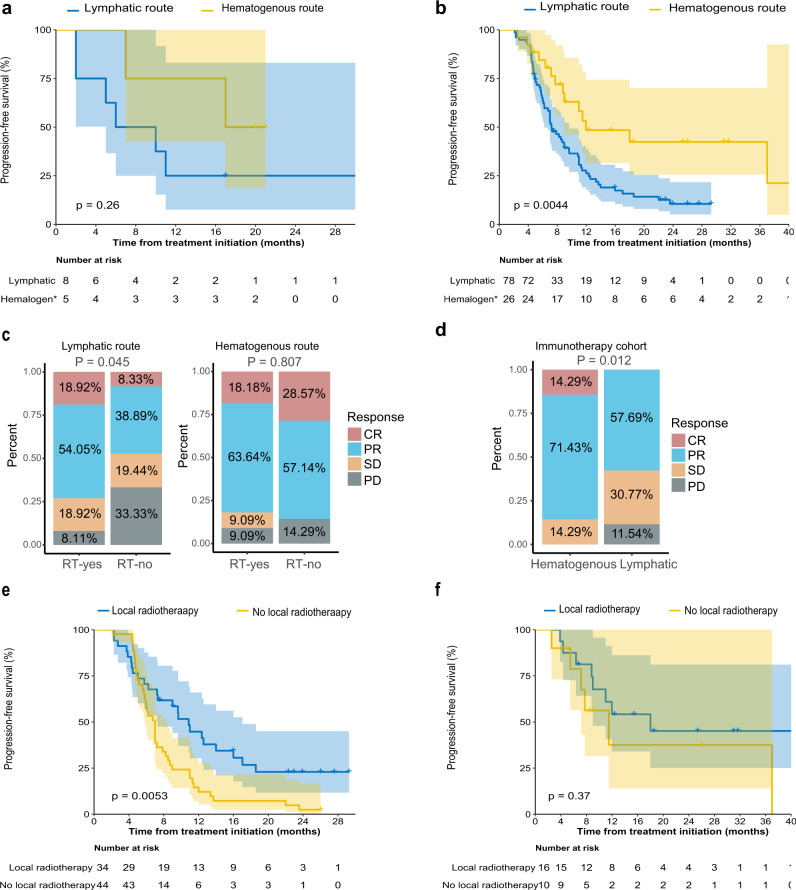
Different clinical outcomes between the “lymphatic” and “hematogenous” metastatic routes. **a** Kaplan‒Meier curves of progression free survival (PFS) in the 13 NPC with identified metastatic models via genomic phylogenetic tree. The data are shown along with 95% confidence intervals. The log-rank test was used to calculate the P value (two-sided). **b** Kaplan‒Meier curves of PFS comparing the radiomics-predicted lymphatic and hematogenous metastatic models in the de novo metastatic NPC cohort. The data are shown along with 95% confidence intervals. The log-rank test was used to calculate the *P* value (two-sided). **c** Bar plot showing the different treatment response rates between patients who received locoregional radiation therapy and those who did not in the primary diagnosed metastatic NPC cohort in the lymphatic (left) and hematogenous (right) models. RT: radiation therapy. **d** Bar plot showing the different response rates to combination immunotherapy between the lymphatic and hematogenous groups in the immunotherapy NPC cohort. **e**, **f** Kaplan‒Meier curves of PFS between metastatic patients who received local radiotherapy and those who did not receive local radiotherapy in patients with metastases emerging via the lymphatic route (**e**) or the hematogenous route (**f**). The data are shown along with 95% confidence intervals. The log-rank test was used to calculate the P value (two-sided).

Among the 13 metastatic NPC patients with genomic evidence, five patients with the hematogenous metastatic route had longer progression-free survival (PFS) times than eight patients with the lymphatic metastatic route (median PFS, 17 months vs. 6 months, *P* = 0.26; Fig. 7a). Consistently, in the 104 de novo metastatic NPC cohort recruited in our randomised, phase 3 trial (NCT02111460; Supplementary Table 4), which was aimed at examining the benefits of locoregional radiotherapy in de novo metastatic NPC, 26 patients with metastases emerging via the hematogenous metastatic route had significantly longer 2-year PFS time than 78 patients with metastases emerging via the lymphatic route (2-y PFS, 42.4% vs. 10.5%, *P* = 0.0044; Fig. 7b). Multivariable analysis further confirmed that patients with metastases emerging via the hematogenous route had better PFS than those with metastases emerging via the lymphatic route (HR = 0.397, 95% CI = 0.191-0.825, *P* = 0.013; Supplementary Table 8).

In the cohort of 104 metastatic NPC patients, locoregional radiotherapy was associated with a significantly higher objective response rate (ORR) than no locoregional radiotherapy in patients with the lymphatic metastatic route when evaluating metastatic lesions at the end of treatment (ORR, 73.0% vs. 47.2%, *P* = 0.045). In contrast, locoregional treatment was not associated with significantly higher ORR than no locoregional radiotherapy in patients with metastases emerging via the hematogenous route (ORR, 81.8% vs. 85.7%, *P* = 0.807; Fig. 7c). Moreover, we found that adding locoregional radiotherapy to the treatment of patients with the lymphatic metastatic route resulted in improved disease control (2-y PFS, 22.9% vs. 2.4%, *P* = 0.005), while patients with the hematogenous metastatic route did not significantly benefit from combined locoregional radiotherapy (2-y PFS, 45.1% vs. 37.5%, *P* = 0.366; Fig. 7e, f). For M0-stage NPC, patients in the hematogenous group achieved better survival outcomes than those in the lymphatic group, which indicated that intense treatment modalities such as aggressive chemoradiotherapy might be needed for the lymphatic group (Supplementary Fig. 10b).

Additionally, in the immunotherapy cohort containing 66 patients who received combination immunotherapy consisting of toripalimab, apatinib and gemcitabine, 14 patients in the hematogenous group achieved a significantly higher ORR than 52 patients in the lymphatic group (ORR, 85.7% vs. 57.7%, *P* = 0.012; Fig. 7d). These findings suggested that the lymphatic and hematogenous metastatic routes might be effective in stratifying patients who are at a high risk of disease progression and might be potentially used to choose specific patients for locoregional radiotherapy or immunotherapy.

## Discussion

Our study provided broad insights into the evolutionary trajectory and characteristics of NPC metastasis. We portrayed a comprehensive genomic landscape of NPC primary, regional lymph node and metastatic tumours. According to the phylogenetic analysis and scRNA-seq analysis, two distinct dissemination routes of distant metastases were observed, including lymphatic dissemination from regional lymph node metastases and hematogenous dissemination from primary tumours. Primary tumours via the lymphatic route were significantly enriched in pathways such as EMT, UV response and angiogenesis, while primary tumours via the hematogenous route were significantly enriched in the IFN-α and IFN-γ response pathways. We successfully utilised radiomics data to categorize NPC metastatic routes into lymphatic metastasis and hematogenous metastasis instead of genomic characteristics. Finally, we observed that adding locoregional radiotherapy to the treatment of patients with the lymphatic metastatic route resulted in improved survival outcomes, while patients with the hematogenous route benefited more from immunotherapy.

Whether metastasis seeding initiates via blood or lymphatic vessels may rest largely on the structural restrictions imposed on invasive tumours. Lymphatic vessels lack the tight junctions and surrounding layers of basement membranes typically seen in blood vessels, which makes lymphatics “leakier” than blood vessels, thus lowering the barriers for tumour cell spreading. Whether tumour cells subsequently disseminate to lymph nodes and distant sites remains elusive. According to previous genomic studies with small sample sizes, the dissemination of tumours cells from primary tumours to distant sites independent of regional lymph nodes appears to be the dominant metastatic route in colorectal cancer^58,59^, lung cancer^60^, breast cancer^61,62^, melanoma^63^ and oesophageal adenocarcinoma^64^. However, ccRCC seems to be a possible exception, as lymph nodes and distant metastases were always found to originate from common subclones^65^. Notably, we observed that the lymphatic route was more prevalent in NPC than the hematogenous route, which is compatible with the clinical observations that 80% of patients with NPC have regional lymph node metastases at diagnosis^66^. Instead of merely describing the phenomenon of different dissemination routes, we confirmed our findings using single-cell sequencing data, further explored the molecular features of different metastatic routes and built a radiomics-based prediction model to conveniently identify the lymphatic and hematogenous routes. In addition to structure-dependent selection, studies have also recently proposed that the choice of hematogenous or lymphatic dissemination might be attributed to different molecular mechanisms driving tumour cells to specific types of metastatic dissemination. The data from in vivo and 3D cultures showed that EMT cells prefer to migrate towards lymphatic vessels rather than blood vessels^67^. Consistently, our data indicate that patients with metastases emerging via the lymphatic route have higher activation of EMT signalling.

Moreover, our data showed that patients with metastases emerging via the hematogenous route had higher expression of IFN-γ response-related genes. IFN-γ upregulates several checkpoint inhibitors, such as PD-L1 and PD-L2, on the surface of tumour cells and cooperates with PD-1 to induce T cell exhaustion, thus suppressing the antitumour immune response^67–69^. Indeed, we observed a significant enrichment of exhausted CD8^+^ T cells in the tumour microenvironment of patients with metastases via the hematogenous route based on scRNA-seq and multi- IHC. The recruitment of immunosuppressive cells to the primary tumour site protects cancer cells from being killed by cytotoxic cells and makes the blood vessels “leaky”, similar to lymphatic vessels, thereby increasing the probability of hematogenous dissemination^70^. Therefore, we hypothesise that for patients with metastases emerging via the hematogenous route, the barriers of blood vessels for tumour cell spreading are lowered due to the impaired antitumour immune response that is caused by IFN-γ response signalling, and immunotherapy should be effective in these patients. As a result, we found that patients with metastases emerging via the hematogenous route had a significantly better response to immunotherapy than patients with metastases emerging via the lymphatic route.

Previous retrospective studies, including ours, have demonstrated that systemic chemotherapy combined with radical locoregional radiotherapy might be beneficial for de novo metastatic NPC patients. However, the survival benefits of locoregional radiotherapy for de novo metastatic NPC patients have not yet been demonstrated in a prospective randomised trial. Recently, we conducted an open-label, phase 3, randomised controlled trial (NCT02111460), which demonstrated that chemotherapy plus radiotherapy significantly improved OS in chemotherapy-sensitive de novo metastatic NPC patients with acceptable toxicity and tolerability^4^. However, the specific population of metastatic NPC patients who could benefit from locoregional radiotherapy remains elusive. Here, we further showed that patients with the lymphatic metastatic route achieved a better response to locoregional radiotherapy than those with the hematogenous metastatic route. We suspect that the locoregional radiotherapy probably blocks the metastatic route of tumour cells that spread to distant sites via regional lymph nodes, but the underlying mechanism still needs further investigation.

Radiomics is an emerging field that converts imaging data into a high dimensional mineable feature space using a large number of automatically extracted data-characterization algorithms^71,72^. A previous study revealed that radiomics data contained strong prognostic information in both lung and head and neck cancer patients and were associated with the underlying gene expression patterns^73^. Therefore, we hypothesise that these imaging features capture the distinct phenotypic differences of tumours and can be used to discriminate the two metastatic routes. Indeed, we found a larger primary tumour size and a smaller number of lower cervical lymph nodes in patients with metastases emerging via the hematogenous route than in those via the lymphatic route. Intriguingly, this was concordant with the results of a big data intelligence platform-based study that showed an ascending type with advanced local disease but early-stage cervical lymph node involvement and a descending type with advanced lymph node disease but early-stage local invasion^74^. Compared to patients with ascending tumours, those with descending tumours had an increased likelihood of distant metastasis, regional recurrence, disease recurrence, and death^74^. The hematogenous route resembled the ascending type, while the lymphatic route resembled the descending type. In addition, we found that survival outcomes were inferior in the lymphatic group compared to the hematogenous group. Then, we built a prediction model and showed that the radiomics model could distinguish the hematogenous and lymphatic routes. The gene expression pattern was similar between the genomic-based route and the radiomic-based route. Moreover, both the genomic-based route and radiomic-based route showed consistent prognostic patterns, indicating that patients with metastases emerging via the hematogenous route have better PFS than those with metastases emerging via the lymphatic route. These findings suggested that the radiomics model was credible and could be used as a noninvasive method to predict the evolutionary routes of NPC metastasis, with potential clinical utility in guiding treatment decision-making.

According to our WES analysis of multiregion tumours, NPC tumours have substantial ITH, which may lead to false discoveries in the construction of evolution routes if only considering single-region sampling. We collected multiregion samples from available patients, and observed that the biopsy of samples might exert limited influence on the classification of metastatic routes, which underlines the importance of multiregion sampling in such studies. Moreover, in the present study, the number of patients was still limited due to the difficulty in obtaining samples from distant metastatic sites, especially in the construction and validation of the radiomics-based prediction model. Although we applied our prediction model in three additional clinical cohorts that lacked a clear label of the metastatic route and observed consistent radiomics features and survival outcomes with the training cohort, the risk that the classification might be incorrect could not be completely avoided. Thus, a large cohort of patients with a multiregion samples is warranted to further validate our conclusions. Circulating tumour cells (CTCs) have recently been widely used to explore the mechanisms of tumour cell dissemination from primary tumours to distant sites. CTCs are an ideal alternative to distant metastasis sampling for constructing a phylogenetic tree to determine the metastasis evolution route.

In conclusion, our study provides important insights into the evolutionary history of distant metastasis in NPC. We provide comprehensive genomic evidence that distant metastases originate from regional lymph node metastases or directly from primary tumours. The two different metastatic routes identified have distinct genomic and clinical characteristics and therapeutic responses. Our study provides strategies for the treatment decision-making of NPC patients with distant metastasis, which might ultimately further improve the survival outcomes of NPC.

## Materials and methods

### Sample and data collection

Patients at Sun Yat-sen University Cancer Center (SYSUCC) (Guangzhou, China) were recruited for this study between June 1, 2012, and May 1, 2016 following the approval of this study by the ethics committee of SYSUCC. For all patients recruited in the present study, a comprehensive pretreatment evaluation that included a complete medical history and physical examination, haematologic and biochemical analyses, nasopharyngoscopic findings, and magnetic resonance imaging (MRI) was conducted. ^18^F-Fluorodeoxyglucose positron emission tomography-computed tomography (^18^F-FDG PET-CT), which can confidently and sensitively detect small tumours^75^, was also mandatory for distant metastasis staging. After a comprehensive evaluation, patients would receive standard treatments provided by the clinicians. Almost all patients received cisplatin-based chemotherapy (97.7%, 43/44), and a total of 24 (54.5%) patients underwent intensity-modulated radiotherapy (IMRT). All the samples taken from these patients were histologically confirmed as nasopharyngeal carcinoma (NPC) (WHO I, II, or III). The quality of the tumour samples was examined by tissue sectioning and haematoxylin & eosin (H&E) staining to estimate the tumour content. Only the highest quality samples with ≥30% tumour content were selected for subsequent study. The full clinical characteristics of the sequenced patients are provided in Supplementary Table 1.

These 104 de novo metastatic NPC patients were all from our clinical trial “Chemotherapy plus radical local-regional radiotherapy compared with chemotherapy alone in primary metastatic nasopharyngeal carcinoma: A randomised, open-label, phase 3 trial” (NCT02111460; Supplementary Table 4). To reduce the batch effect, only patients present in SYSUCC were included. In addition, patients without high-quality MRI image data (3 T MRI) were excluded from the subsequent radiomics analysis. A total of 54 patients were treated with six cycles of cisplatin and 5-fluorouracil chemotherapy plus locoregional radiotherapy, and a total of 50 patients were treated with six cycles of cisplatin and 5-fluorouracil chemotherapy alone. Tumour response at the end of treatment was based on the Response Evaluation Criteria in Solid Tumours (RECIST) v1.1 and assessed by nasopharyngoscopy and MRI for the primary site and ^18^F-FDG PET-CT, CT or MRI for distant lesions. The patients were followed up every two to three months until death to evaluate the efficacy and safety of the treatment.

A total of 201 nonmetastatic primary NPC patients were recruited for this study between January 1, 2010, and January 1, 2013 (Supplementary Table 6). These patients had not previously received chemotherapy or radiotherapy when diagnosed. All nonmetastatic NPC patients received standard treatments including IMRT with a radical dose, and almost all patients (183/201, 91.04%) were treated with cisplatin-based chemotherapy combined with radiotherapy.

The immunotherapy cohort contained 66 patients confirmed to have progressive disease (PD) during follow-up (Supplementary Table 7). In other words, these patients were refractory to at least one line of systemic therapy. All these patients were clinically treated at SYSUCC from January 2019 to July 2019 and had not been enrolled in any clinical trials. Among them, 55 patients experienced metastatic lesion relapse, and 11 patients experienced only locoregional lesion relapse. Since the survival outcomes were generally inferior for these patients and there was no standard treatment, we organised the consultation of doctors in our department to determine the treatment modality for each patient. After carefully weighing the advantages and disadvantages of different treatment modalities, all doctors in our department approved the application of the combination of gemcitabine, apatinib and toripalimab as the salvage treatment modality (off-label). All patients then received apatinib (anti-VEGFR) via oral administration, 250 mg, once a day; gemcitabine (chemotherapy) 1000 mg/m^2^ (Day 1 and Day 8); and toripalimab (anti-PD-1), 200 mg/kg dose (Day 1) every 21 days for at most 6 cycles, followed by toripalimab 200 mg every three weeks (Q3W) and apatinib once a day maintenance for the remainder of the study or until documented PD. All patients who received the combination treatment provided written informed consent to receive this therapy.

This study was approved by the ethics committee of SYSUCC (B2022-413-01). All patients provided written informed consent to participate in the study.

### Nucleic acid extraction

A section was cut from frozen blocks and stained with H&E. An expert NPC pathologist reviewed the slides to determine and circle the area with the highest tumour content. Guided by the H&E-stained slides, the region with the highest tumour content was cut from the frozen blocks, pulverised using CryoPrep (Covaris, Woburn, MA) and homogenized in lysis buffer from the AllPrep RNA/DNA/Protein Mini Kit (Qiagen, Valencia, CA). DNA, RNA and protein were isolated from each sample using the respective kits (Qiagen, Valencia, CA) following the manufacturer’s protocol.

### Whole-genome/whole-exome sequencing

For whole-genome sequencing (WGS), a total of 0.8 μg of genomic DNA per sample for patients with high molecular weight (>20 kb single band) was used for DNA library preparation. A sequencing library was generated using the TruSeq Nano DNA HT Sample Prep Kit (Illumina, USA) following the manufacturer’s recommendations, and index codes were added to each sample. In brief, the genomic DNA sample was fragmented to a size of ~350 bp by a Covaris sonication system. Then, DNA fragments were end-polished, A-tailed, and ligated with the full-length adapter for Illumina sequencing, followed by further PCR amplification. After PCR products were purified (AMPure XP system), libraries were analysed for size distribution by the Agilent 2100 Bioanalyzer and quantified by real-time PCR (3 nmol/L). The clustering of the index-coded samples was performed on a cBot Cluster Generation System using the HiSeq X PE Cluster Kit v2.5 (Illumina) according to the manufacturer’s instructions. After cluster generation, the DNA libraries were sequenced on the Illumina HiSeq X platform, and 150 bp paired-end reads were generated.

For whole-exome sequencing (WES), qualified genomic DNA from tumours and matched peripheral blood was fragmented by Covaris technology with resultant library fragments of 180–280 bp, and then adaptors were ligated to both ends of the fragments. The extracted DNA was then amplified by ligation-mediated PCR (LM-PCR), purified, and hybridized to the Agilent SureSelect Human Exome V6 for enrichment, and nonhybridized fragments were then washed out. Both uncaptured and captured LM-PCR products were subjected to real-time PCR to estimate the magnitude of enrichment. Each captured library was then loaded onto the Illumina HiSeq X platform, and we performed high-throughput sequencing for each captured library independently to ensure that each sample met the desired average fold coverage.

### SSNV/InDel and SCNA calling from WGS/WES

We used a commercial variant detection pipeline named Sentieon, which improves upon BWA-^50^, GATK-^51^, and Mutect-^52^ based pipelines, to call SSNVs and short insertion/deletions (InDels). Based on this pipeline, the 2×150 bp paired-end reads were mapped into the human reference genome (UCSC hg38), and SSNVs and InDels were called after the BAM file was sorted and deduplicated.

To further reduce false-positive variant calls, additional filtering was performed. A single nucleotide variant (SNV) was considered a true positive if the supported read counts for this SNV were ≥5, and the *P* calculated by Fisher’s test between the mutant read count and the wild-type read count was <0.05. Variants in variant call format (VCF) were annotated using ANNOVAR^53^.

To detect significantly mutated genes, we first filtered mutations frequently detected (minor allele frequency (MAF) > 0.001) in normal databases, including the 1000 Genome (2015 Aug, http://www.internationalgenome.org/), ESP6500 (version esp6500siv2, https://esp.gs.washington.edu/drupal/) and ExAC (version ExAC03, http://exac.broadinstitute.org/) databases. Then MutSigCV^54^ was used to define significantly mutated genes in each sample group (primary, regional lymph node and distant metastatic tumours). To avoid the statistical bias induced by repeat sampling in each group, we merged all mutations of repeat samples in each patient of each sample group before MutSigCV analysis. A gene with a *P* less than 0.0001 was considered to be significantly mutated.

Somatic copy number variants (SCNVs) were called using Control-FREEC v11.1^55^. The GISTIC2 algorithm^34^ was used to infer recurrently amplified or deleted genomic regions in primary tumours, regional lymph nodes and distant metastases. To avoid the statistical bias induced by repeat sampling in each group, we randomly selected only one sample from each patient in each group to perform GISTIC2 analysis. G-scores were calculated for genomic and gene-coding regions based on the frequency and amplitude of amplifications or deletions affecting each gene. We obtained “amplification (AMP)” and “deletion (DEL)” alterations at the gene level based on the “high-level amplification (or deletion) thresholds of segment mean” provided by GISTIC2. The key genes with CNVs represented in this paper were selected from previous studies^6–8,76–78^.

### Variant call validation

To determine the accuracy of the somatic variant calls, we randomly selected all 32 non-silent mutation sites (29 SSNVs and 3 InDels of a total of 64 mutations across all selected samples) from the most recurrently mutated genes to perform Sanger sequencing validation. The location of the mutation site was used to retrieve the adjacent genomic sequence in the UCSC Genome Browser (https://genome.ucsc.edu/), and targeted primers were designed with Primer 3 software (http://primer3.ut.ee/) based on the genomic sequence obtained from UCSC. We used PCR with the designed primers to amplify the desired DNA template for the targeted region and then performed Sanger sequencing. All mutations were successfully validated except two mutations that failed in primer design and three mutations that failed in sequence amplification.

### Whole-exome imputation of SSNVs and InDels

Multisampling sequencing provides the opportunity to increase the sensitivity to detect low frequency mutations. By sharing the independently called mutations across the multiple regions and reassessing the reads at each position for each tumour region, it is possible to call more mutations and reduce the possibility of overrepresenting the mutational heterogeneity. SAMtools v1.3.1^79^ mpileup with the parameter “-p 20 -P 20” was used to extract read information across all tumour regions where a variant (SSNV or InDel) was detected in one or more regions in this patient. For somatic variants that were not called ubiquitously across tumour regions, the missing variants were fetched back if the mutant read count was ≥3 and the read depth was > 10. If the read depth was ≤ 10, we marked this site in the specific region as “NA”.

### Mutation signature analysis

We first identified de novo-derived mutational signatures for primary, regional lymph node and metastatic tumour samples separately using the signature analysis module in maftools v2.0.10^80^. As a result, we obtained four, five and five signatures for primary, regional lymph node and metastatic tumours, respectively. Cosine similarity was then calculated to map the de novo-derived signatures to the known signatures in the COSMIC database. A signature with a cosine similarity greater than 0.5 is considered an interpretable signature. The de novo-derived signatures in primary tumours were mapped to signatures 2, 4 and 6; the de novo-derived signatures in regional lymph node tumours were mapped to signatures 2, 5, 6 and 13; and the de novo-derived signatures in distant metastatic tumours were mapped to signatures 4, 6 and 13. We then applied the R package “Palimpsest v1.0.0”^81^ in all the samples to estimate the contribution of signatures 2, 4, 5, 6 and 13, as well as signature 1, which has been found in all cancer types and in most cancer samples. Palimpsest was also used to estimate the probability of each mutation being due to each process to predict the mechanisms at the origin of driver events, by which we estimated the contribution of each signature in each branch of the phylogenetic tree and determined the most dominant signature of each branch (branches with less than 15 mutations were ignored during this analysis).

### Bulk RNA sequencing

Total RNA was extracted from approximately 10^6^ freshly collected NPC cells following standard TRIzol RNA extraction protocols. RNA-seq libraries were prepared from 500 ng of total RNA using the Illumina TruSeq Stranded Total RNA Kit. Libraries were barcoded and pooled on the Illumina HiSeq X platform. We performed transcriptome sequencing (RNA-seq) on primary tumour samples from 28 patients (P02, P04-P05, P07-P08, P10-P12, P14-P16, P20-P23, P26, P28, P30, P33-P34, and P40-P48; Supplementary Data 2).

### Bulk RNA-seq analysis

The 150 bp paired-end reads from RNA sequencing (RNA-seq) were mapped to the human reference genome (UCSC hg38) using STAR v020201^82^. RSEM v1.3.0^83^ was then used to perform gene expression quantification. DESeq2 v1.20.0^84^ was used to perform differential expression analysis. The log_2_TPM normalised data were used in the clustering and correlation analysis.

### 10x Genomics single-cell RNA sequencing (scRNA-seq)

For experiments using the 10x Genomics platform, the Chromium Single Cell 3’ Library & Gel Bead Kit V2 (PN- 120237), the Chromium Single Cell 3’ Chip Kit V2 (PN-120236) and the Chromium i7 Multiplex Kit (PN-120262) were used according to the manufacturer’s instructions in the Chromium Single Cell 3’ Reagents Kits V2 User Guide. The single-cell suspension was washed twice with 1x PBS + 0.04% BSA.

The cell number and concentration were confirmed with a TC20™ Automated Cell Counter. Approximately 5000 cells were immediately injected into the 10x Genomics Chromium Controller machine for Gel Beads-in-Emulsion (GEMs) generation. mRNA was prepared using the 10x Genomics Chromium Single Cell 3’ Reagent Kit (V2 chemistry). During this step, cells were partitioned into GEMs along with gel beads coated with oligos. These oligos provided poly-dT sequences to capture mRNAs released after cell lysis inside the droplets, as well as cell-specific and transcript-specific barcodes (16 bp 10x barcode and 10 bp unique molecular identifier (UMI), respectively). After real-time PCR, cDNA was recovered, purified and amplified to generate sufficient quantities for library preparation. Library quality and concentration were assessed using an Agilent Bioanalyzer 2100. Libraries were run on the HiSeq X or NovaSeq platform for Illumina PE150 sequencing. In total, 11 samples of two patients (P14, P15) were subjected to scRNA-seq, including 2 primary tumour samples, 4 regional lymph node metastasis samples and five distant metastasis samples.

### 10x Genomics scRNA-seq data preprocessing

The CellRanger v2.1.1 (10x Genomics) analysis pipeline was used to perform original computational analysis of single-cell sequencing data. In general, raw sequencing data (bcl files) were converted to FASTQ format files with Illumina’s bcl2fastq tool (“cellranger mkfastq”); the FASTQ files were aligned to the human genome reference sequence (UCSC hg38), and the raw single-cell gene expression matrix was quantified (“cellranger count”). The outputs of each region from the same patient were aggregated for sequencing depth normalization (“cellranger aggr”), and then an experiment-wide gene-barcode matrix was generated for further analysis.

Expression matrix filtering and clustering algorithms for tumour-normal cell classification were implemented and performed using Seurat v3.0^85^. First, genes detected (UMI count >0) in less than 5 cells were removed. In brief, cells with small (detected genes <200) or large (detected genes > 5000) library sizes and those with a mitochondrial genome transcript ratio >10% were removed. The gene expression measurements for each cell were normalised by the total expression, multiplied by a scale factor (10,000) and log-transformed. Highly variable genes were calculated and used for principal component analysis (PCA) to reduce the number of dimensions representing each cell, and “significant” principal components (PCs) were manually determined by looking at a plot of the standard deviations of the PCs following Seurat’s suggestions. Then, a shared nearest neighbour graph-based clustering approach was performed, and clusters were visualised using t-distributed stochastic neighbour embedding of the PCs (spectral t-SNE) as implemented in Seurat.

Since NPC is a cancer that originates in the epithelium, we distinguished malignant cells from nonmalignant cells using known epithelial markers, such as *KRT14*, *KRT17*, and *EPCAM*. Then, we selected widely recognised markers of possible cell types in our samples and mapped their expression levels into cell clusters. The scores of functional modules for CD8^+^ T-cell clusters were calculated using the AddModuleScore function in Seurat at the single-cell level. The exhausted gene set included *HAVCR2*, *LAG3*, *TIGIT*, *CTLA4*, *PDCD1* and *LAYN*. The cytotoxicity gene set included *GZMA*, *GZMB*, *GZMM*, *NKG7*, *GNLY* and *PRF1*.

### Processing and analysis of public scRNA-seq data

Public scRNA-seq data (accession numbers: GSE123813 and PRJNA705464) of basal cell carcinoma and clear cell renal cell carcinoma samples from the initial publication were downloaded and reanalyzed for this manuscript. First, the dead or dying cells with more than 10% mitochondrial RNA content were removed, and the cells with too low of a number (less than 200) were also removed. Cell doublets were predicted using DoubletFinder with default parameters. Then, the filtered gene expression matrix for each sample was normalised using the “NormalizeData” function in Seurat, and only highly variable genes were retained using the “FindVariableFeatures” function in Seurat. Next, the “Runharmony” function in harmony were used to integrate the gene expression matrices of all samples, where batch effects between different samples were adjusted. Then, the “RunPCA” function was used to perform the PCA, and the “FindNeighbors” function was used to construct a K-nearest neighbour graph. Next, the most representative PCs selected based on PCA were used for clustering analysis with the “FindCluster” function to determine different cell types. Finally, UMAP was used to visualise the different cell types. We identified the cluster with high expression of *CD3G*, *CD3D* and *CD3E* as T cells. *CD4* and *CD8A* gene expression was used to differentiate CD4^+^ and CD8^+^ T cells. Subcluster of CD4^+^ T cells and CD8^+^ T cells were named by the top marker gene.

### Reconstruction of phylogenetic trees

To reconstruct phylogenetic trees based on SSNVs and InDels, we firstly constructed a mutation binary matrix based on mutations whose information was still available across all samples after imputation. Considering the influence of genomic losses on mutation detection, we filtered mutations that met the following conditions: 1) the mutation was not present in all the samples of the same patient; and 2) there was a loss of heterozygosity (LOH) detected by Control-FREEC in this region of the sample(s) without this mutation. We then used the neighbour-joining (NJ) method^86–88^ implemented in the R package “APE v5.0”^89^ to construct phylogenetic trees based on the mutation binary matrix. The NJ method takes an S×M binary matrix D as input, where D*ij* = 1 if the *jth* mutation is observed in the *ith* sample. To estimate the robustness of each phylogenetic tree, we performed bootstrapping for the internal nodes of each NJ tree using the Tree Bipartition and Bootstrapping Phylogenies (*boot.phyo*) function in the R package “APE”. Based on the “boot.phylo” function, we performed a put-back sampling of all mutations with the number of samplings equal to the total number of mutations. Thus, a new mutation binary matrix was constructed, and the new matrix was used to construct the phylogenetic tree. This analysis was carried out 1000 times, and finally the number of times that each branch node of the original tree reappears during the thousand reconstructions was counted. We used the bootstrap reproducibility of branch nodes as a reliability evaluation metric for distinguishing the two modes (progression model probability), and determined that a reliable model classification should have a reproducibility greater than 75%. The length of each branch was adjusted to reflect the number of shared mutations in that branch. Driver genes with non-silent mutations or CNVs (AMP/DEL) were marked on each branch based on the sharing across all samples.

### Classification of the origin of distant metastasis

The evolutionary route of distant metastasis was determined based on the phylogenetic tree that was reconstructed using the NJ tree method. The evolutionary route of the metastatic lesion was “lymphatic” if the node closest to the metastatic lesion was prior to the regional lymph node; otherwise, it was “hematogenous”. We quantified the branching confidence in the inferred evolutionary tree by bootstrapping with 1000 iterations.

### Cancer cell fraction (CCF) estimation of variants

The ABSOLUTE v1.0.6^90^ algorithm was used to estimate the tumour sample purity, ploidy, and CCF of each SSNV, InDel and CNV. In line with the recommended best practice, all ABSOLUTE solutions were reviewed by 3 researchers, with solutions selected based on majority vote. In this analysis, variants (SSNVs, InDels and CNVs) were classified as either clonal or subclonal based on the confidence interval of the CCF evaluated by ABSOLUTE. Mutations were defined as clonal if the 95% confidence interval overlapped by 1 and as subclonal otherwise.

### Variant clustering and subclone-based evolution analysis

All variants (SSNV, InDels and CNVs) were collected for clustering and subclone-based evolutionary analysis. For individual samples, we inferred the number of subclones and the fraction of cells within each subclone using an algorithm named “density-based spatial clustering of applications with noise (DBSCAN)”^91,92^ to cluster mutations according to their putative CCF values in all related samples. The DBSCAN algorithm was performed based on Euclidean distance, with the number of core points set as 1 and the support radius set as 0.05. A cluster with more than 10 mutations was considered a subclone that arose during tumour evolution (except the founding cluster, which means the CCF (see below) in all samples was more than 0.9).

The phylogenetic tree was constructed based on the CCF value and adjustable CCF interval of each subclone. The CCF value of a subclone in one sample was determined as the median value of the CCF of all mutations belonging to this subclone in this sample normalised by the founding clone’s CCF in this sample. The adjustable CCF interval of each subclone was calculated as follows:

1 $$H=F+k\sqrt{\frac{{\sum }_{i=1}^{n}({{h}_{i}-{f}_{i}})^{2}}{n}}$$

2 $$L=F-k\sqrt{\frac{{\sum }_{i=1}^{n}{(l_{i}-{f}_{i})}^{2}}{n}}$$

where *F* is the CCF value of the subclone, and *H* and *L* are the adjustable CCF upper and lower bounds of the subclone, respectively; *f*_*i*_ is the CCF value of the *i*_*th*_ mutation site in the subclone, and *h*_*i*_ *and l*_*i*_ are the upper and lower bounds of the confidence interval of *i*_*th*_ mutation site in the subclone, respectively. *k* is the expansion factor, which was defined as 3 in this study.

The evolutionary relationship between two subclones could be divided into two categories. The first category is called the “containment relationship”; that is, one subclone is the “parent” of another subclone during tumour evolution. The other relationship is called the “parallel relationship”, which means that these two subclones are not the parent of each other and belong to different cell lineages during evolution.

The first step of evolutionary relationship analysis is to construct the backbone of the phylogenetic tree based on the specific subclonal relationships:

$${L}_{i}+{L}_{j} \, > \, 1\to {subclone}\,i\,{{{{{\rm{is}}}}}}\,{{{{{\rm{subclone}}}}}}\,{j}^{{\prime} }{{{{{\rm{s}}}}}}\,{{{{{\rm{parent}}}}}}\,{{{{{\rm{or}}}}}}\,j\,{{{{{\rm{is}}}}}}\,{i}^{{\prime} }{{{{{\rm{s}}}}}}\,{{{{{\rm{parent;}}}}}}$$

$${L}_{i} \, > \, {H}_{j}\to {{{{{\rm{subclone}}}}}}\,j\,{{{{{\rm{is}}}}}}\,{{{{{\rm{not}}}}}}\,{{{{{\rm{subclone}}}}}}\,{i}^{{\prime} }{{{{{\rm{s}}}}}}\,{{{{{\rm{parent}}}}}}.$$

If the relationship between subclone *i* and subclone *j* is different from the above two relationships, there is no definite relationship between the two subclones, and further investigation is needed. After the relationship between two subclones is determined, the backbone of the phylogenetic tree is established according to the containment and noncontainment relationships.

The second step of evolutionary relationship analysis is to add the remaining subclones to their possible positions in the phylogenetic tree based on CCF values in order from large to small. The distant values that CCF needs to be adjusted for each possible situation are calculated, and the phylogenetic tree with the smallest adjustment value is chosen as the final evolution model. If a small subclone could be added to multiple places of the phylogenetic tree without CCF adjustment, these phylogenetic trees would be merged and only show one result. We used modified functions of the R package “ClonEvol”^93^ to visualise the results.

To assess the robustness of the above analysis, we used bootstrapping, subsampling 1000 times from the number of clustered mutations with replacement. Then, the phylogenetic tree was reconstructed according to the different CCF values and confidence intervals and compared with the original phylogenetic tree to determine whether the two results were consistent.

### Selection of events during metastasis

Based on the clonality across all sample regions of each variant, we determined the selected, novel, founding and unselected classes of variants during metastasis:

1. variants that are selected (“selected”, *n* = 1193 mutations, defined as variants that are clonal in metastatic tumours but subclonal or not found in seeding donor tumours);
2. variants that are novel (“novel”, *n* = 3603 mutations, defined as variants that are subclonal in metastatic tumours but not found in seeding donor tumours);
3. variants that are founding (“founding”, *n* = 718 mutations, defined as variants that are clonal in both metastatic tumours and seeding donor tumours);
4. variants that are unselected (“unselected”, *n* = 5634 mutations, defined as variants that are not found in metastasis but are clonal or subclonal in seeding donor tumours)

The relationship of metastasis with seeding donor tumour was identified by both the phylogenetic tree and tumour subclonal architecture. If one metastasis had an uncleared seeding donor, for example, primary or lymph node metastasis, or primary-1 or primary-2, we selected the larger mutation CCF as the seeding donor’s mutation clonality.

### Determination of the evolutionary route of malignant cells based on scRNA-seq

To characterize the potential evolutionary routes in the process of NPC metastasis, we performed pseudotime analysis for malignant cells, using Monocle2^52^ (version 2.8.0). The data of the indicated clusters calculated in Seurat were fed directly into Monocle2. Next, we carried out density peak clustering (Monocle2 dpFeature procedure) to order cells based on the genes with differential expression between clusters, using the differentialGeneTest function in Monocle2. The top 1000 significant genes (ordered by q value) were used for ordering in all instances. Then the evolutionary trajectory was inferred after dimension reduction and cell ordering with the default parameters of Monocle2.

### Multiplex immunohistochemistry (multi-IHC)

To validate the enrichment of exhausted CD8^+^ T cells in the microenvironment of patients with the hematogenous metastatic route, formalin-fixed paraffin-embedded (FFPE) slides from 13 NPC primary tumours with a complete genomic based phylogenetic tree were subjected to multi-IHC and multispectral imaging using the PANO 7-plex IHC Kit (cat 0004100100, Panovue, Beijing, China), to examine specific cell markers, including CD8A (Cell Signalling Technology, 70306), CXCL13 (Abcam, ab246518), and TIM3 (Cell Signalling Technology, 45208). Different primary antibodies were sequentially applied, followed by horseradish peroxidase-conjugated secondary antibody incubation and tyramide signal amplification. The slides were microwave heat-treated after each TSA operation. Nuclei were stained with 4′−6′-diamidino-2-phenylindole (DAPI, Sigma-Aldrich) after all the human antigens had been labelled.

To obtain multispectral images, the stained slides were scanned using the Mantra System (PerkinElmer, Waltham, Massachusetts, US), which captured the fluorescence excitation spectrum at 20-nm wavelength intervals (420–720 nm) within the same exposure time. Multiple scans were combined to build a single stack image. The spectrum of autofluorescence of the tissues and each fluorescein was extracted from the images of unstained and single-stained sections to establish a spectral library required for multispectral unmixing by InForm image analysis software (PerkinElmer, Waltham, Massachusetts, US). Using this spectral library, the reconstructed images of sections were obtained with the autofluorescence removed.

### Construction of the machine learning prediction model based on radiomics data

Contrast-enhanced T1-weighted (CE-T1W) MRI images were used to build the classification models. For each patient, all the slices with tumour were selected. Regions of interest (ROIs) were first manually drawn by three experienced radiologists manually using the software Analyze Pro (https://analyzedirect.com/analyzepro/). They were required to cautiously draw all discernible tumour regions along axial directions, in which the images had a high resolution of 0.43 mm × 0.43 mm. After that, we applied the classical active contour model to obtain the segmented ROI for some small or tiny tumours in MATLAB. The labelled boundary drawn by the radiologists was used to initialize the active contour. Any disagreements were resolved through negotiation until consensus was reached by the three experts. The raw image type we used was DICOM.

Within each ROI, we computed the radiomics features for each pixel centred by a sliding window with a size of 11 × 11. A total of 192 radiomics features were extracted for each sliding window. The radiomics features included three types of features, namely, statistical, texture and Gabor features^94–96^. (1) Statistical features: the grey value of the central point, momentum with order 1 to 5, was used. (2) Texture features: grey level co-occurrence matrices (GLCMs) with offsets of [−3, −1; −1, 0; 0, 1; 0, 3; 1, −1; 1, 3; and 2, −2] and angles of 0, 45, 90 and 135 were calculated. Twenty-two statistical features were extracted, including energy, entropy, dissimilarity, contrast, inverse difference, correlation, homogeneity, autocorrelation, cluster shade, cluster prominence, maximum probability, sum of squares, sum average, sum variance, sum entropy, difference variance, difference entropy, two kinds of information measures of correlation, maximal correlation coefficient, inverse difference normalised and inverse difference moment normalised. A total of 7 × 22 = 154 GLCM-related features were extracted from each sliding window. (3) Gabor features: Each ROI was filtered by 32 Gabor filters with wavelengths of 2.83, 5.66, 11.31, and 22.63 and eight orientations to obtain 32 filtered images. A total of 4 × 8 = 32 Gabor features were extracted from each sliding window. All the feature extraction methods were implemented based on built-in functions in MATLAB and the formulas below. After obtaining the radiomics features for each pixel, we computed the averaged value and used it to quantify the corresponding patient.

We then performed recursive feature elimination (RFE), to find the feature subset with the highest prognostic accuracy^97^. The identified feature subset consisted of ten features and achieved the highest accuracy of 0.8462. The accuracy was dramatically less than that using the whole feature set (Supplementary Fig. 9a); thus, we used all the features to build the prediction classifier. In the training cohort, we first built a K- nearest neighbour (KNN) classifier with correlation distance K = 1 to categorize each patient into one of the two groups. The input variables were radiomics features with the corresponding binary label, determined by the molecular subtypes as hematogenous metastasis or lymphatic metastasis. The leave-one-out cross-validation scheme, a popular method that is very suitable for small datasets, was employed to train the model to achieve optimal performance^98^. In practice, we first prepared Q candidate models (M1,…,MQ) and calculated the error E1,…, EQ of each learning result. We choose the model with the smallest error E1…, EQ as the final model. The constructed classifier then served as a baseline to evaluate the metastasis pattern for a new query patient. In the validation cohort, each patient was assigned one of the metastasis patterns based on his or her radiomics features. After the metastasis patterns were obtained for the validation cohort, the survival risks were estimated for the two groups to compute their significant differences.

### Functional enrichment analysis

Gene enrichment was performed using the R package “clusterProfiler v3.8.1”^99^. clusterProfiler implements a hypergeometric model to test for gene set overrepresentation relative to a given background gene set.

### Statistics

R 3.5.1 was used for all statistical analyses. Parameters such as sample size, number of replicates, number of independent experiments, and the measures of centre, dispersion, and precision (mean ± SD or SEM) and statistical significance are reported in the Figures and Figure Legends. The results were considered statistically significant when *P* < 0.05 or a lower threshold when indicated by the appropriate test (analysis of variance (ANOVA), *t* test, or Pearson correlation). Student’s *t* test, permutation test, and hypergeometric test were used for comparisons in experiments with two sample groups. In experiments with more than two sample groups, ANOVA was performed followed by Bonferroni’s post hoc test. Survival analysis was performed using the Kaplan-Meier (KM) method. The log-rank test was used to evaluate the significance of the difference between different KM curves. The hazard ratio was determined using a Cox proportional hazards model.

### Reporting summary

Further information on research design is available in the Nature Portfolio Reporting Summary linked to this article.

## Supplementary information

Supplementary Information

Description to Additional Supplementary Files

Supplementary Data 1

Supplementary Data 2

Supplementary Data 3

Supplementary Data 4

Supplementary Data 5

Supplementary Data 6

Supplementary Data 7

Reporting Summary

## Data Availability

The raw sequence data generated in this paper have been deposited in the Genome Sequence Archive (GSA, Genomics, Proteomics & Bioinformatics 2017) in the BIG Data Center (Nucleic Acids Res 2018), Beijing Institute of Genomics (BIG), Chinese Academy of Sciences [http://bigd.big.ac.cn/gsa-human/]. The genomic sequencing data is available under accession number HRA000034, the transcriptomic sequencing data is available under accession number HRA000035, and the single-cell sequencing data is available under accession number HRA000036. The publicly available scRNA-seq data of basal cell carcinoma used in this study are available in the Gene Expression Omnibus (GEO) database under accession number GSE123813. The publicly available scRNA-seq data of clear cell renal cell carcinoma used in this study are available at https://www.ncbi.nlm.nih.gov/sra/PRJNA705464. The remaining data are available within the Article, Supplementary Information or Source Data file. Source data are provided with this paper.

## Source data

Source Data

## Code availability

The codes used for subclone-based evolution analysis are available in GitHub (https://github.com/sys2019/cloneTree). Other custom codes are provided in Supplementary Data 7.
