# Supplementary material for: Evolutionary route of nasopharyngeal carcinoma metastasis and its clinical significance
Source: Nat Commun. 2023 Feb 4;14:610. doi: 10.1038/s41467-023-35995-2 (PMC9899247; doi:10.1038/s41467-023-35995-2)
Supplement: Supplementary file 1 — Supplementary Information [file 41467_2023_35995_MOESM1_ESM.pdf]

SUPPLEMENTARY FIGURES

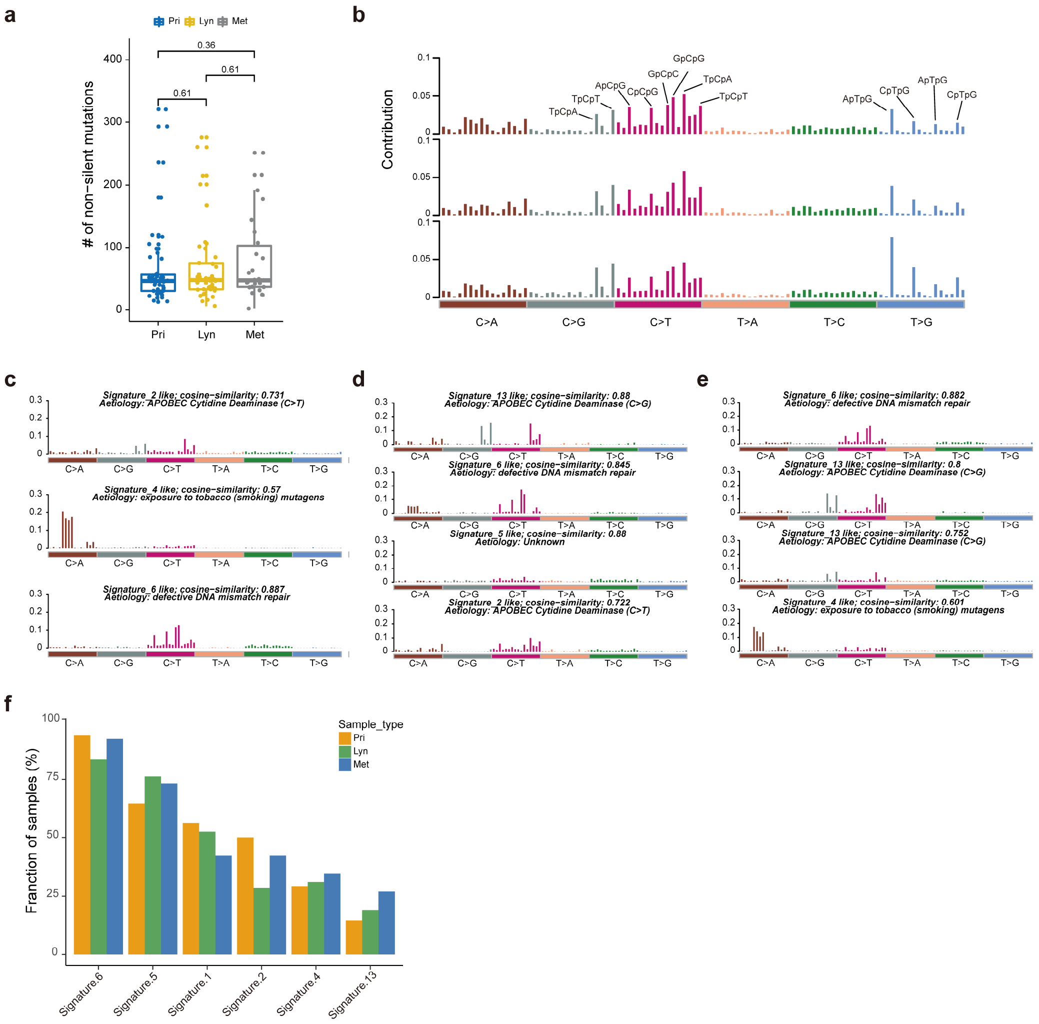

Supplementary Fig. 1, related to Fig. 1. Mutation comparison among primary tumours, and regional lymph node and distant metastases. (a) The numbers of detected nonsilent mutations were similar among Pri (n = 48), Lyn (n = 42) and Met (n = 26). In each box plot, the centre line represents the median, the bounds represent the first and third quartiles and whiskers extend from the hinge to the largest value no further than 1.5 * interquartile range (IQR) from the hinge. A two-sided Wilcoxon signed-rank test was used to calculate P values; “ns”: $P \geqslant 0.05$. (b) Similar mutation context (defined by the proportion of A, T, C and G nucleotides within ±2 bp of the variant site) for six mutation categories among Pri (top), Lyn (middle) and Met (bottom). Remarkable mutation contexts are highlighted. (c-e) Signatures decomposed by nonnegative matrix factorization of Pri (c), Lyn (d) and Met (e). Each signature

was compared against known signatures derived from the COSMIC database (https://cancer.sanger.ac.uk/cosmic/signatures), and cosine similarity was calculated to identify the best match. Signatures with a cosine similarity of less than 0.3 were filtered out. (f) Fraction of samples that contributed to the specific signature among Pri, Lyn and Met. Source data are provided as a Source Data file.

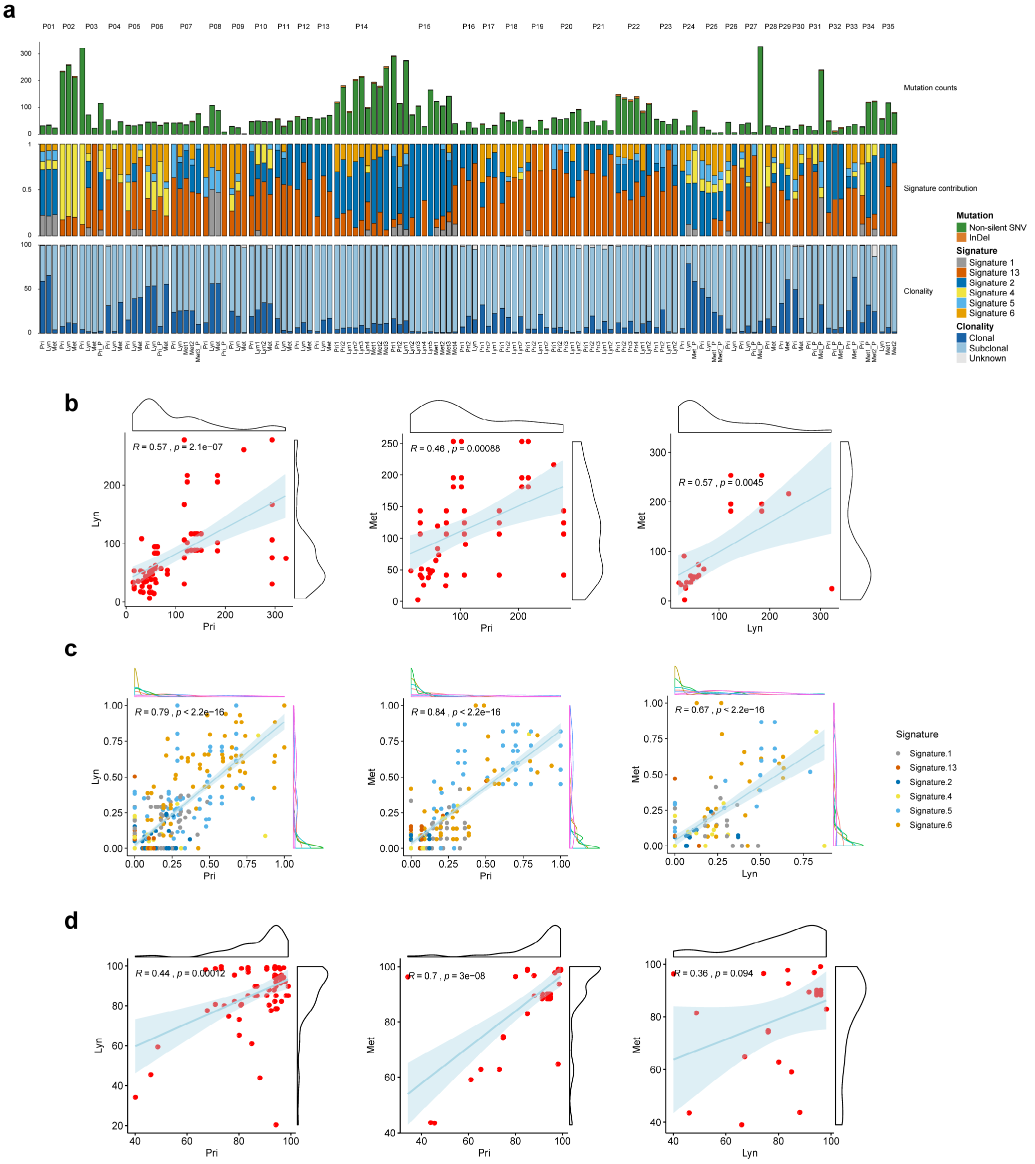

Supplementary Fig. 2, related to Fig. 1. Longitudinal analysis of mutation signatures for each sample. (a) Bar plot showing the number of mutations (top), composition of mutation signatures (meddle) and proportion of clonal mutations (bottom) for each sample. (b) Scatter plot showing the correlation relationship of the number of mutations in primary vs. regional lymph node tumours (left), primary vs. distant metastasis tumours (mediate) and regional lymph node vs. distant metastasis tumour (right). The data are shown along with 95% confidence intervals. Pearson correlation analysis was used to calculate P values (two-sided). (c) Scatter plot showing the correlation of the contribution of different mutation signatures in primary vs. regional lymph node tumours (left), primary vs. distant metastasis tumours (middle) and regional lymph node vs. distant metastasis tumours (right). The data are shown along with 95%

confidence intervals. Pearson correlation analysis was used to calculate P values (two-sided). (d) Scatter plot showing the correlation of the proportion of subclone mutations in primary vs. regional lymph node tumours (left), primary vs. distant metastasis tumours (middle) and regional lymph node vs. distant metastasis tumours (right). The data are shown along with 95% confidence intervals. Pearson correlation analysis was used to calculate P values (two-sided). Pri: primary tumour; Lyn: regional lymph node tumour; Met: distant metastasis tumour. Source data are provided as a Source Data file.

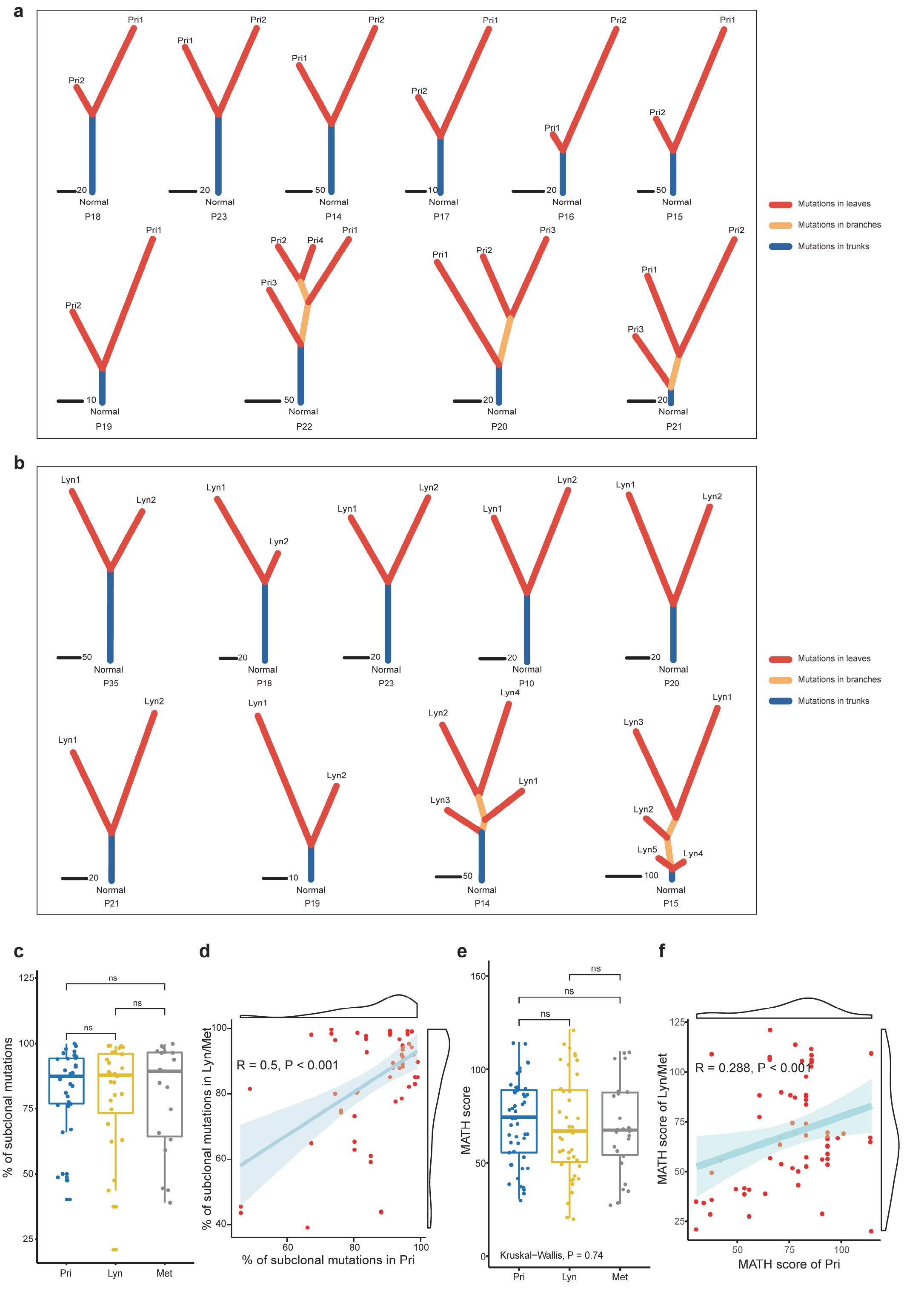

Supplementary Fig. 3, related to Fig. 1. Heterogeneity analysis of primary tumours, regional lymph node metastases and distant metastases. (a) Phylogenetic trees showing intratumour heterogeneity (ITH) based on multiregion samples of primary tumours. The length of the branch reflects the number of mutations and short trunks indicate high level mutation

heterogeneity. Blue, the trunk of the tree indicates mutations shared by all regions; yellow, branches shared by at least two regions; red, the leaves where mutations were detected only in individual regions. (b) Phylogenetic trees showing ITH based on multiregion samples of NPC regional lymph nodes. (c) No significant difference in the percentage of subclonal mutations was detected among Pri (n = 48), Lyn (n = 42) and Met (n = 26). In each box plot, the centre line represents the median, the bounds represent the first and third quartiles and whiskers extend from the hinge to the largest value no further than 1.5 * interquartile range (IQR) from the hinge. A two-sided Wilcoxon signed-rank test was used to calculate P values; "ns" : $P \geqslant 0.05$. (d) Scatter plot showing the correlation of the proportion of subclone mutations between paired Pri and Lyn/Met samples. The data are shown along with 95% confidence intervals. Pearson correlation analysis was used to calculate P values (two-sided). (e) No significant difference in the MATH score was detected among Pri (n = 48), Lyn (n = 42) and Met (n = 26). In each box plot, the centre line represents the median, the bounds represent the first and third quartiles, and whiskers extend from the hinge to the largest value no further than 1.5 * interquartile range (IQR) from the hinge. A two-sided Wilcoxon signed-rank test was used to calculate P values. (f) Scatter plot showing the correlation of the MATH score between paired Pri and Lyn/Met samples. The data are shown along with 95% confidence intervals. Pearson correlation analysis was used to calculate P values (two-sided). Source data are provided as a Source Data file.

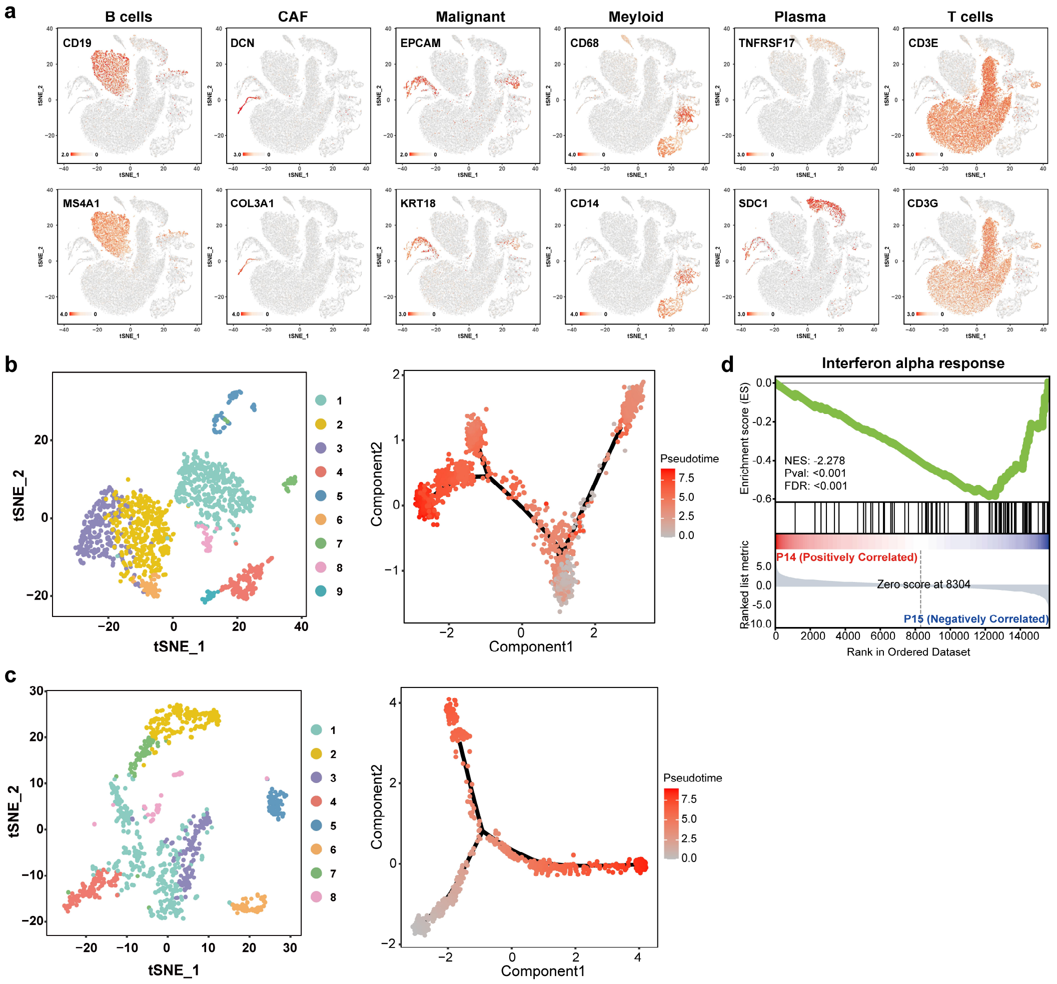

Supplementary Fig. 4, related to Fig. 4. Cell clusters of single cells from the lymphatic and hematogenous routes.

(a) t-SNE projection of single cells from P14 and P15, with cells coloured based on the average expression of sets of marker genes for B cells, cancer-associated fibroblasts, malignant cells, myeloid cells, plasma cells and T cells. (b & c) Pseudotime trajectory analysis of tumour cells from P14 (b) and P15 (c). The left panel shows the subclusters of tumour cells, and the right panel shows the evolutionary trajectory predicted by Monocle2, which is coloured according to the pseudotime score. (d) Hallmark GSEA of P14 primary tumour cells vs. P15 primary tumour cells using scRNA-seq data. An empirical phenotype-based permutation test (two-sided) was used to calculate the P value and false discovery rate (FDR).

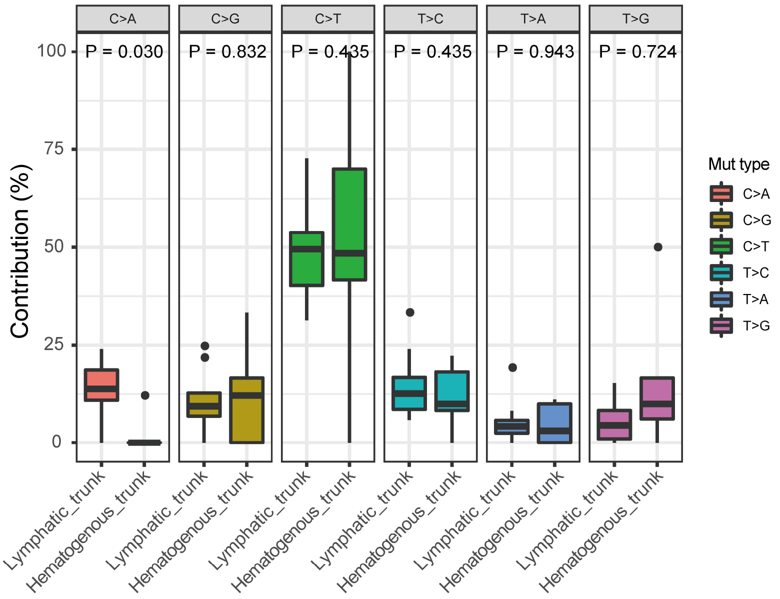

Supplementary Fig. 5, related to Fig. 5. Trunk mutation type comparison of the lymphatic (n = 8) and hematogenous (n = 5) evolutionary models. In each box plot, the centre line represents the median, the bounds represent the first and third quartiles and whiskers extend from the hinge to the largest value no further than 1.5 * interquartile range (IQR) from the hinge. After adjusting for age, gender and tumor stage by using covariance analysis model, two-sided Wilcoxon signed-rank test was used to calculate the adjusted P values. Source data are provided as a Source Data file.

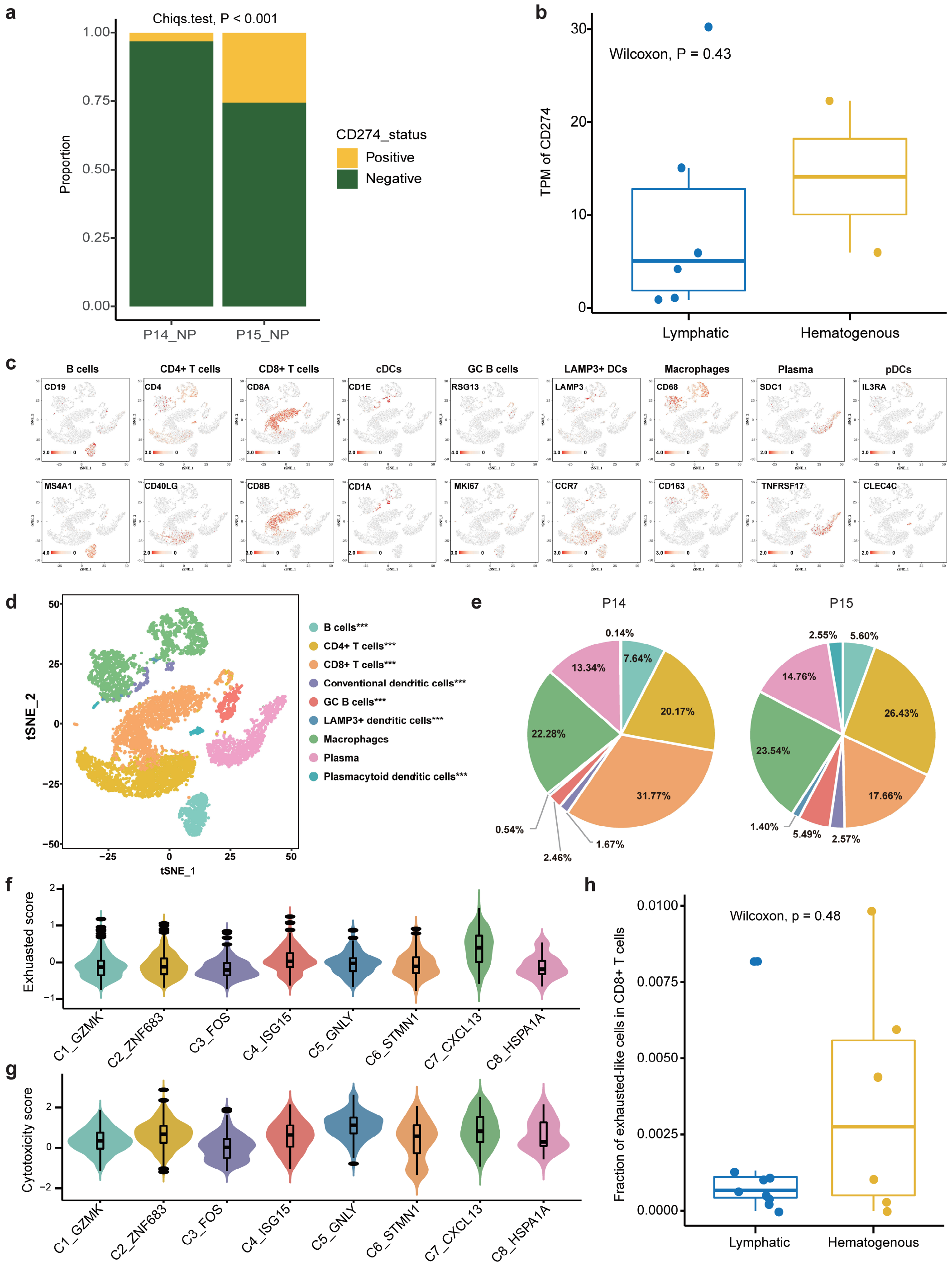

Supplementary Fig. 6, related to Fig. 6. Microenvironment of P14 and P15 revealed by scRNA-seq. (a) Bar plot showing the proportion of PD-L1^{+} tumour cells in the primary tumour of P14 and P15. A two-sided chi-square test was used to calculate the P value. (b) Box plot comparing the expression of *PD-L1* between patients with metastases emerging via the hematogenous route (n = 2) and those with metastases emerging via the lymphatic route (n =

6). In each box plot, the centre line represents the median, the bounds represent the first and third quartiles, and whiskers extend from the hinge to the largest value no further than 1.5 * interquartile range (IQR) from the hinge. A two-sided Wilcoxon signed-rank test was used to calculate P values. (c) t-SNE projection of immune cells from the primary sites of P14 and P15, with cells coloured based on the average expression of sets of marker genes for B cells, $CD4^+$ T cells, $CD8^+$ T cells, classical dendritic cells (cDCs), germinal centre B cells, $LAMP3^+$ DCs, macrophages, plasma cells and plasmatic DCs. (d) t-SNE plot of immune cells from the primary sites of P14 and P15. Clusters are assigned to inferred cell types based on canonical marker expression. The statistical significance of the difference in the proportion of each cell type between P14 and P15 was measured using a two-sided Fisher exact test ("***", $P < 0.001$). (e) Pie charts show the fraction of each immune cell type from primary tumours, which are coloured according to the cell type shown in (b). (f & g) Violin plots comparing the exhaustion score (f) and cytotoxicity score (g) among different $CD8^+$ T-cell subclusters. (h) Box plot showing the ratio of $CXCL13^+TIM3^+CD8^+$ T cells/$CD8^+$ T cells in primary tumour between the lymphatic (n = 8) and hematogenous (n = 5) routes via multiplex IHC staining. In each box plot, the centre line represents the median, the bounds represent the first and third quartiles, and whiskers extend from the hinge to the largest value no further than 1.5 * interquartile range (IQR) from the hinge. A two-sided Wilcoxon signed-rank test was used to calculate P values. Source data are provided as a Source Data file.

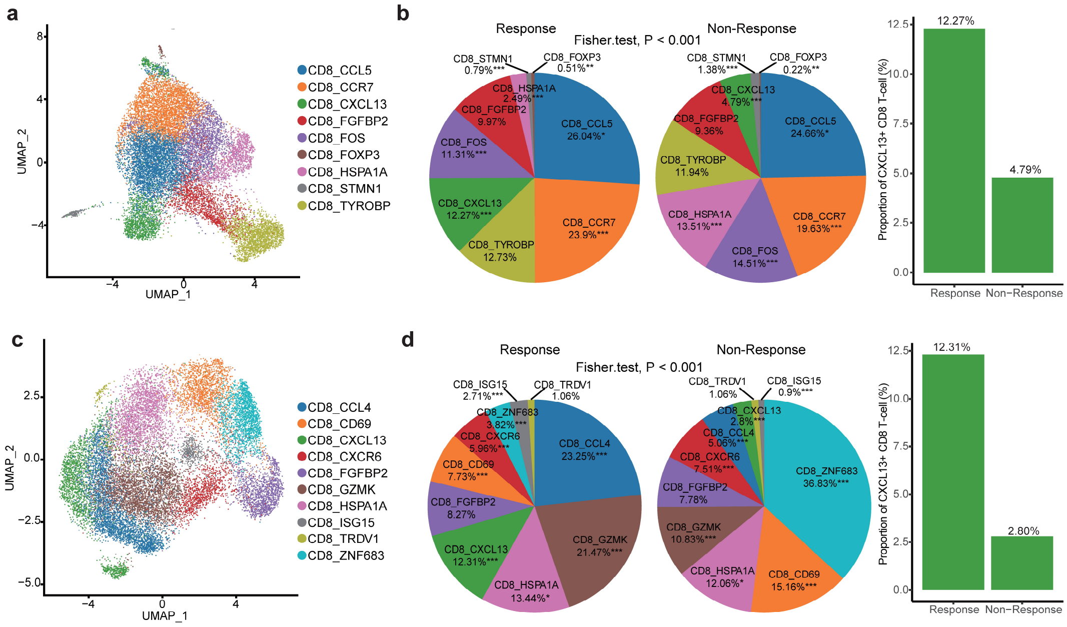

Supplementary Fig. 7, related to Fig 6. CXCL13^{+} CD8^{+} T cells were enriched in patients sensitive to immunotherapy. (a) t-SNE plot showing the clusters of CD8^{+} T cells from BCC patients receiving PD-1 blockade (GSE123813). (b) Pie plot showing that CXCL13^{+} CD8^{+} T cells were significantly enriched in BCC patients who responded to PD-1 blockade, and column plot (right) showing the different proportion of CXCL13+ CD8+ T cells between responders and non-responders (*, P < 0.05; **, P < 0.01, ***, P < 0.001). (c) t-SNE plot showing the clusters of CD8^{+} T cells from ccRCC patients receiving immune checkpoint blockade (PRJNA705464). (d) Pie plot showing that CXCL13^{+} CD8^{+} T cells were significantly enriched in ccRCC patients who responded to immune checkpoint blockade, and column plot (right) showing the different proportion of CXCL13+ CD8+ T cell between responders and non-responders (*, P < 0.05; **, P < 0.01, ***, P < 0.001).

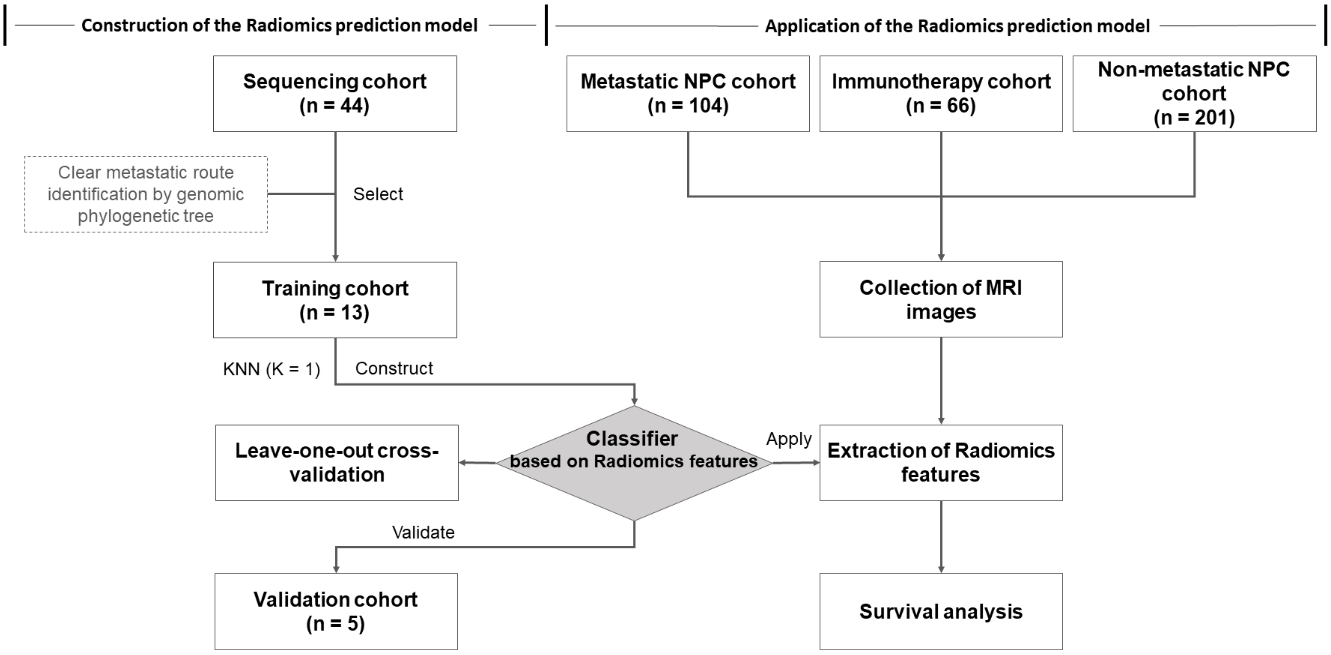

Supplementary Fig. 8, related to Fig. 6. Flow chart of building the prediction model based on radiomics data.

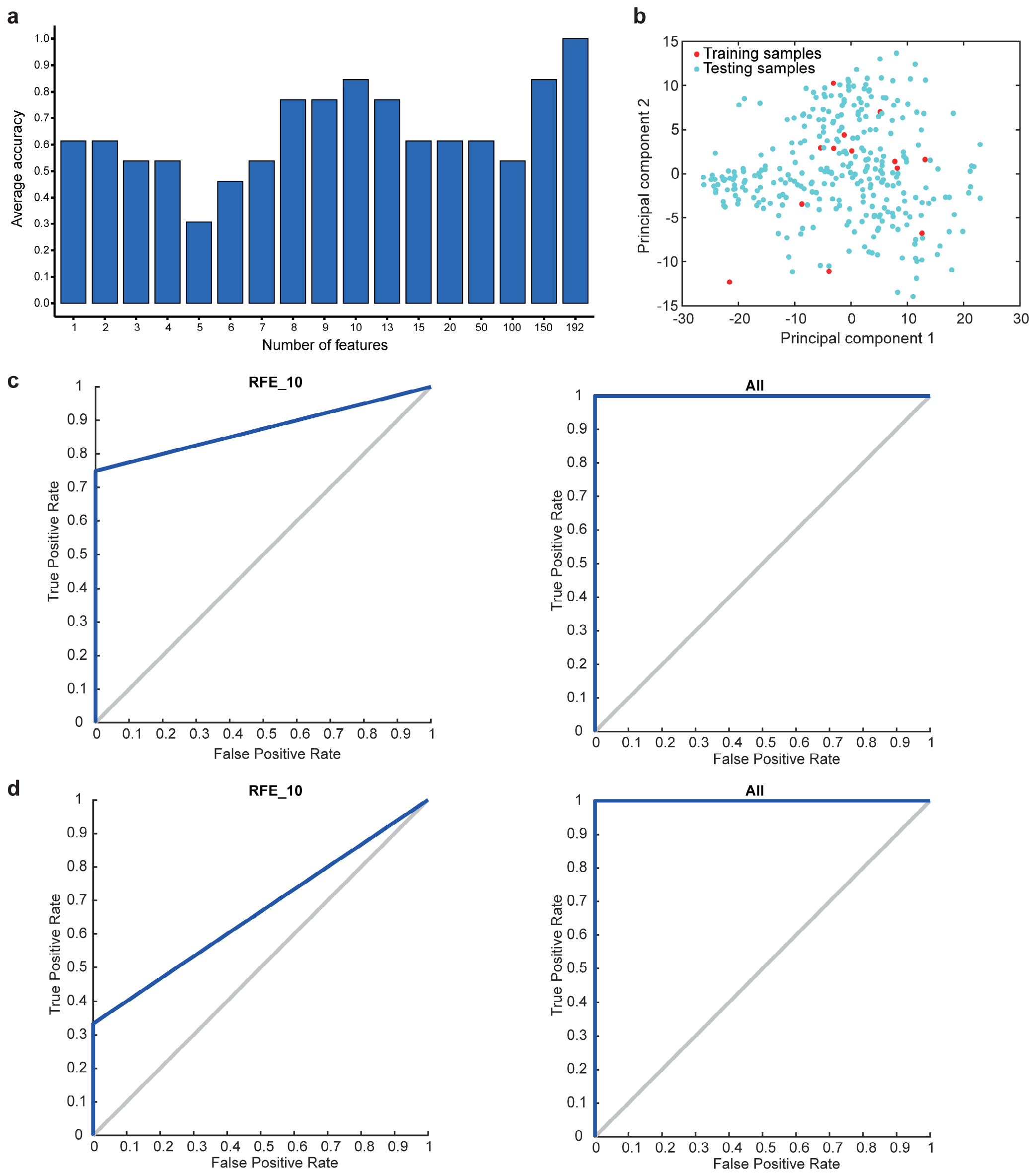

Supplementary Fig. 9, related to Fig. 6. Construction of the radiomics prediction model. (a) Accuracy of different features selection using the recursive feature elimination (REF) algorithm. (b) PCA result from the training and testing samples based on the 192 dimensional features. (c) ROC curve of the training samples using the top ten features selected by the RFE method (left) and whole feature set (right). (d) ROC curve of the classifier on additional validation patients (P49-P53), using the top ten features selected by the RFE method (left), and the whole feature set (right). Source data are provided as a Source Data file.

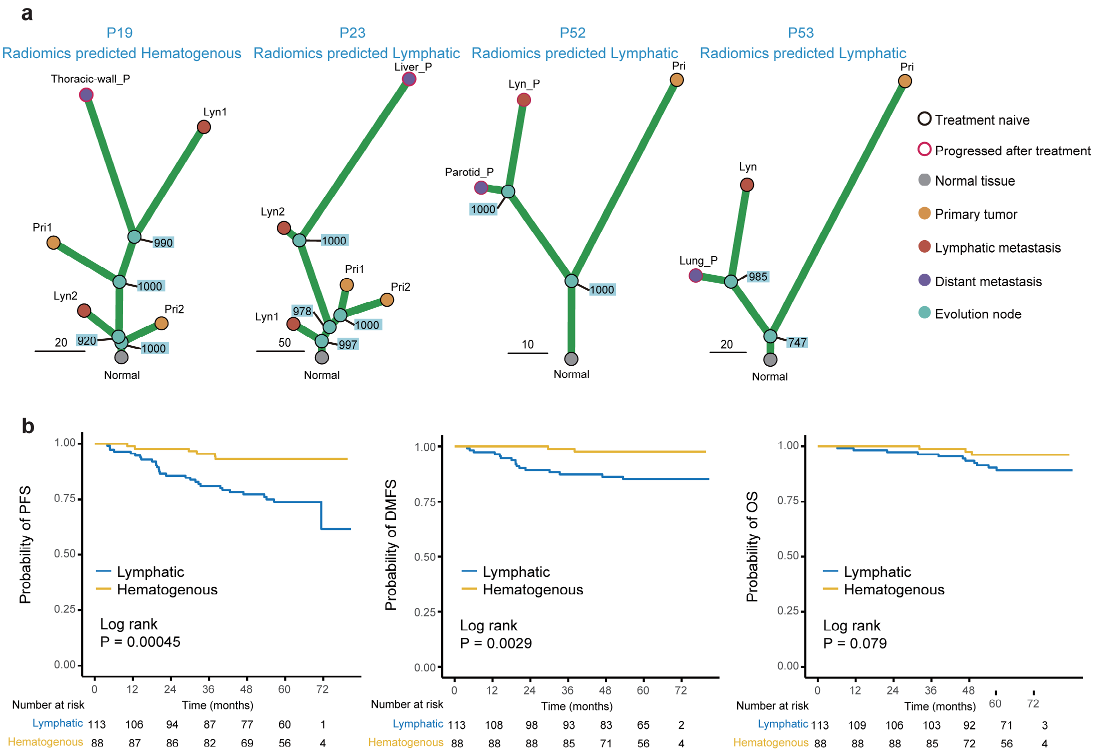

Supplementary Fig. 10, related to Fig. 6. Validation of the radiomics prediction model. (a) Radiomics prediction model results and phylogenetic trees for patients who developed progression during follow-up (P19 and P23) or patients newly collected (P52, P53) to further validate the radiomics prediction model. (b) Kaplan–Meier curves of PFS (left), DMFS (middle) and OS (right) in the M0-stage NPC cohort (n = 201).

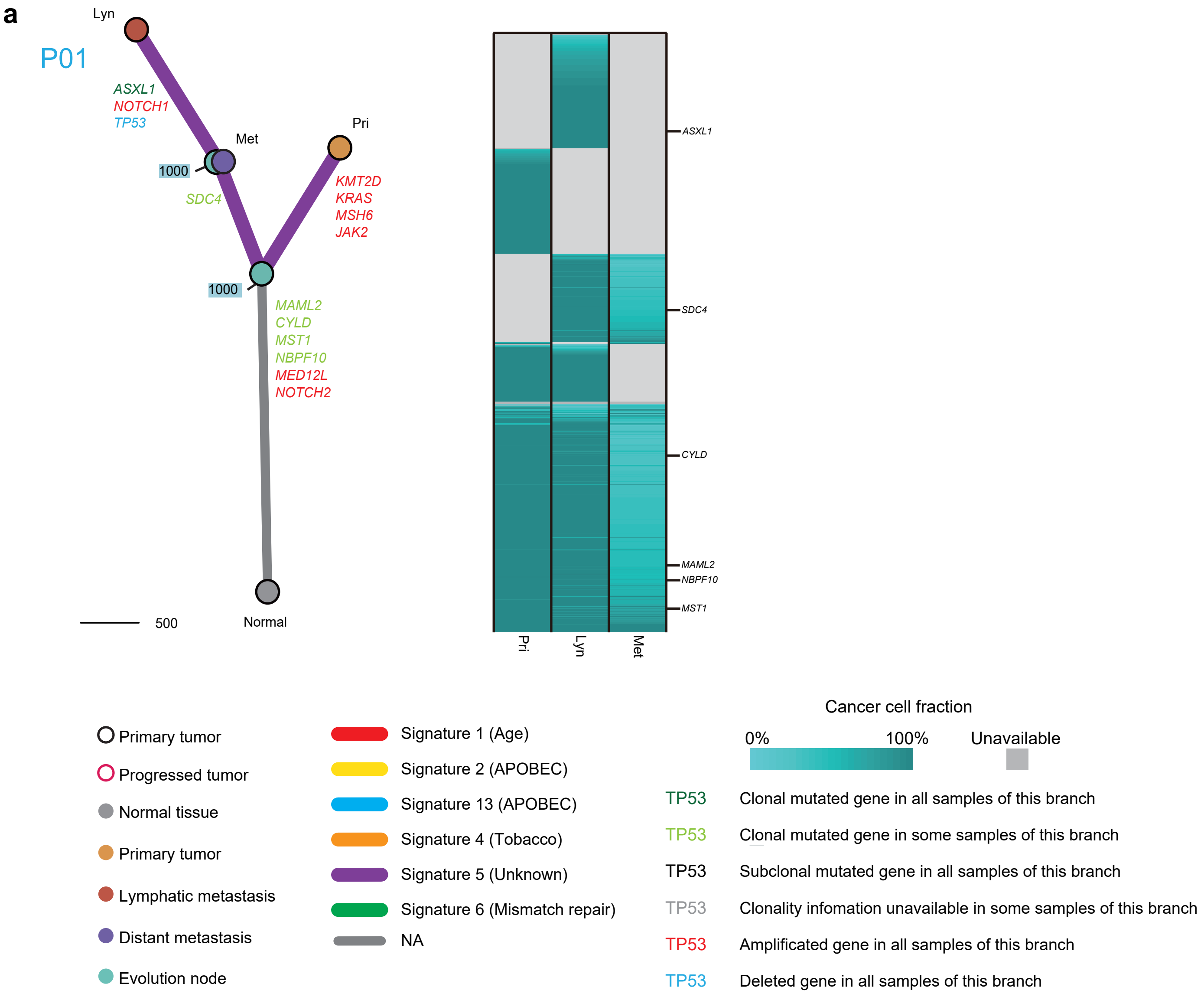
a
P01
Lyn
ASXL1
NOTCH1
TP53
Met
Pri
1000
SDC4
KMT2D
KRAS
MSH6
JAK2
1000
MAML2
CYLD
MST1
NBPF10
MED12L
NOTCH2
500
Normal
ASXL1
SDC4
CYLD
MAML2
NBPF10
MST1
Pri
Lyn
Met
Cancer cell fraction
0%
100%
Unavailable
Primary tumor
Progressed tumor
Normal tissue
Primary tumor
Lymphatic metastasis
Distant metastasis
Evolution node
Signature 1 (Age)
Signature 2 (APOBEC)
Signature 13 (APOBEC)
Signature 4 (Tobacco)
Signature 5 (Unknown)
Signature 6 (Mismatch repair)
NA
TP53 Clonal mutated gene in all samples of this branch
TP53 Clonal mutated gene in some samples of this branch
TP53 Subclonal mutated gene in all samples of this branch
TP53 Clonality infomation unavailable in some samples of this branch
TP53 Amplificated gene in all samples of this branch
TP53 Deleted gene in all samples of this branch

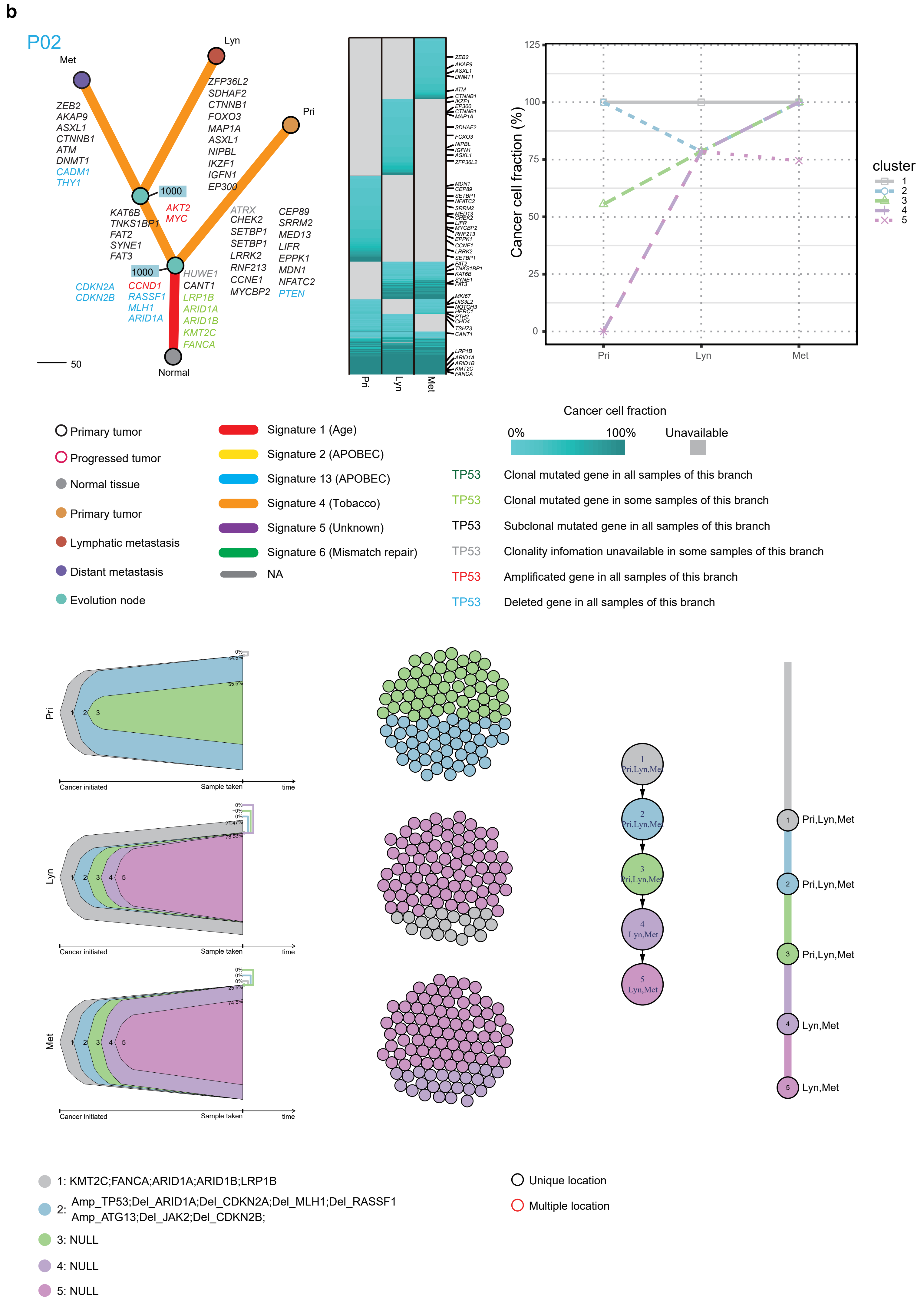
b
P02
Met
Lyn
Pri
Normal
ZEB2
AKAP9
ASXL1
CTNNB1
ATM
DNMT1
CADM1
THY1
ZFP36L2
SDHAF2
CTNNB1
FOXO3
MAP1A
ASXL1
NIPBL
IKZF1
IGFN1
EP300
1000
KAT6B
TNKS1BP1
FAT2
SYNE1
FAT3
AKT2
MYC
ATRX
CHEK2
SETBP1
SETBP1
LRRK2
RNF213
CCNE1
MYCBP2
CEP89
SRRM2
MED13
LIFR
EPPK1
MDN1
NFATC2
PTEN
1000
CDKN2A
CDKN2B
CCND1
RASSF1
MLH1
ARID1A
HUWE1
CANT1
LRP1B
ARID1A
ARID1B
KMT2C
FANCA
50
Pri
Lyn
Met
Cancer cell fraction (%)
125
100
75
50
25
0
cluster
1
2
3
4
5
Cancer cell fraction
0%
100%
Unavailable
Primary tumor
Progressed tumor
Normal tissue
Primary tumor
Lymphatic metastasis
Distant metastasis
Evolution node
Signature 1 (Age)
Signature 2 (APOBEC)
Signature 13 (APOBEC)
Signature 4 (Tobacco)
Signature 5 (Unknown)
Signature 6 (Mismatch repair)
NA
TP53 Clonal mutated gene in all samples of this branch
TP53 Clonal mutated gene in some samples of this branch
TP53 Subclonal mutated gene in all samples of this branch
TP53 Clonality infomation unavailable in some samples of this branch
TP53 Amplificated gene in all samples of this branch
TP53 Deleted gene in all samples of this branch
Pri
Lyn
Met
Cancer initiated
Sample taken
time
1 Pri,Lyn,Met
2 Pri,Lyn,Met
3 Pri,Lyn,Met
4 Lyn,Met
5 Lyn,Met
1: KMT2C;FANCA;ARID1A;ARID1B;LRP1B
2: Amp_TP53;Del_ARID1A;Del_CDKN2A;Del_MLH1;Del_RASSF1
Amp_ATG13;Del_JAK2;Del_CDKN2B;
3: NULL
4: NULL
5: NULL
Unique location
Multiple location

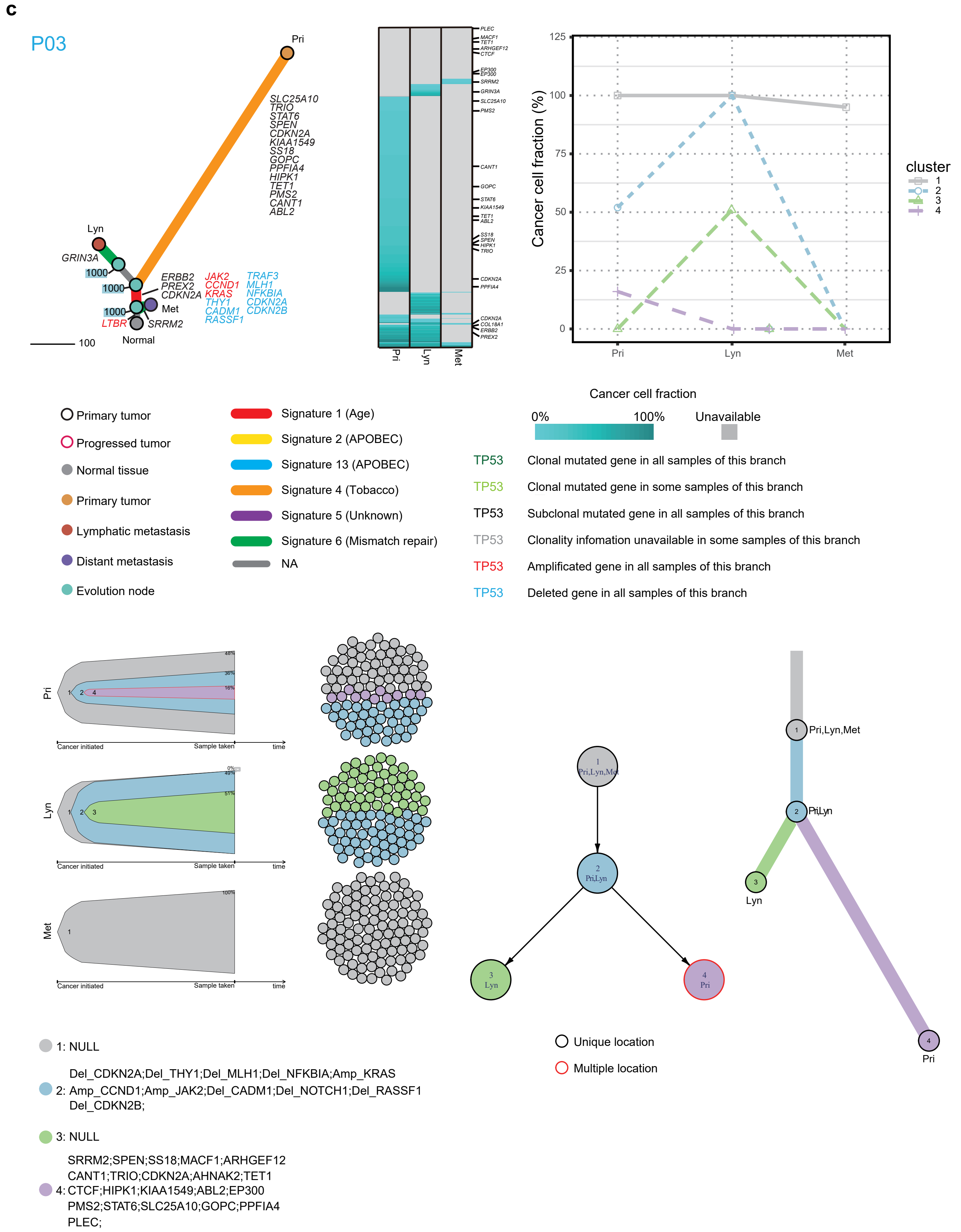

c
P03
Pri
Lyn
Met
Normal
SLC25A10
TRIO
STAT6
SPEN
CDKN2A
KIAA1549
SS18
GOPC
PPFIA4
HIPK1
TET1
PMS2
CANT1
ABL2
GRIN3A
1000
ERBB2
PREX2
CDKN2A
JAK2
CCND1
KRAS
THY1
CADM1
RASSF1
TRAF3
MLH1
NFKBIA
CDKN2A
CDKN2B
LTBR
SRRM2
100
Cancer cell fraction (%)
125
100
75
50
25
0
cluster
1
2
3
4
Cancer cell fraction
0%
100%
Unavailable
Primary tumor
Progressed tumor
Normal tissue
Primary tumor
Lymphatic metastasis
Distant metastasis
Evolution node
Signature 1 (Age)
Signature 2 (APOBEC)
Signature 13 (APOBEC)
Signature 4 (Tobacco)
Signature 5 (Unknown)
Signature 6 (Mismatch repair)
NA
TP53 Clonal mutated gene in all samples of this branch
TP53 Clonal mutated gene in some samples of this branch
TP53 Subclonal mutated gene in all samples of this branch
TP53 Clonality infomation unavailable in some samples of this branch
TP53 Amplificated gene in all samples of this branch
TP53 Deleted gene in all samples of this branch
Cancer initiated
Sample taken
time
48%
36%
16%
0%
49%
51%
100%
1 Pri,Lyn,Met
2 Pri,Lyn
3 Lyn
4 Pri
Unique location
Multiple location
1: NULL
2: Del_CDKN2A;Del_THY1;Del_MLH1;Del_NFKBIA;Amp_KRAS
Amp_CCND1;Amp_JAK2;Del_CADM1;Del_NOTCH1;Del_RASSF1
Del_CDKN2B;
3: NULL
4: SRRM2;SPEN;SS18;MACF1;ARHGEF12
CANT1;TRIO;CDKN2A;AHNAK2;TET1
CTCF;HIPK1;KIAA1549;ABL2;EP300
PMS2;STAT6;SLC25A10;GOPC;PPFIA4
PLEC;

d

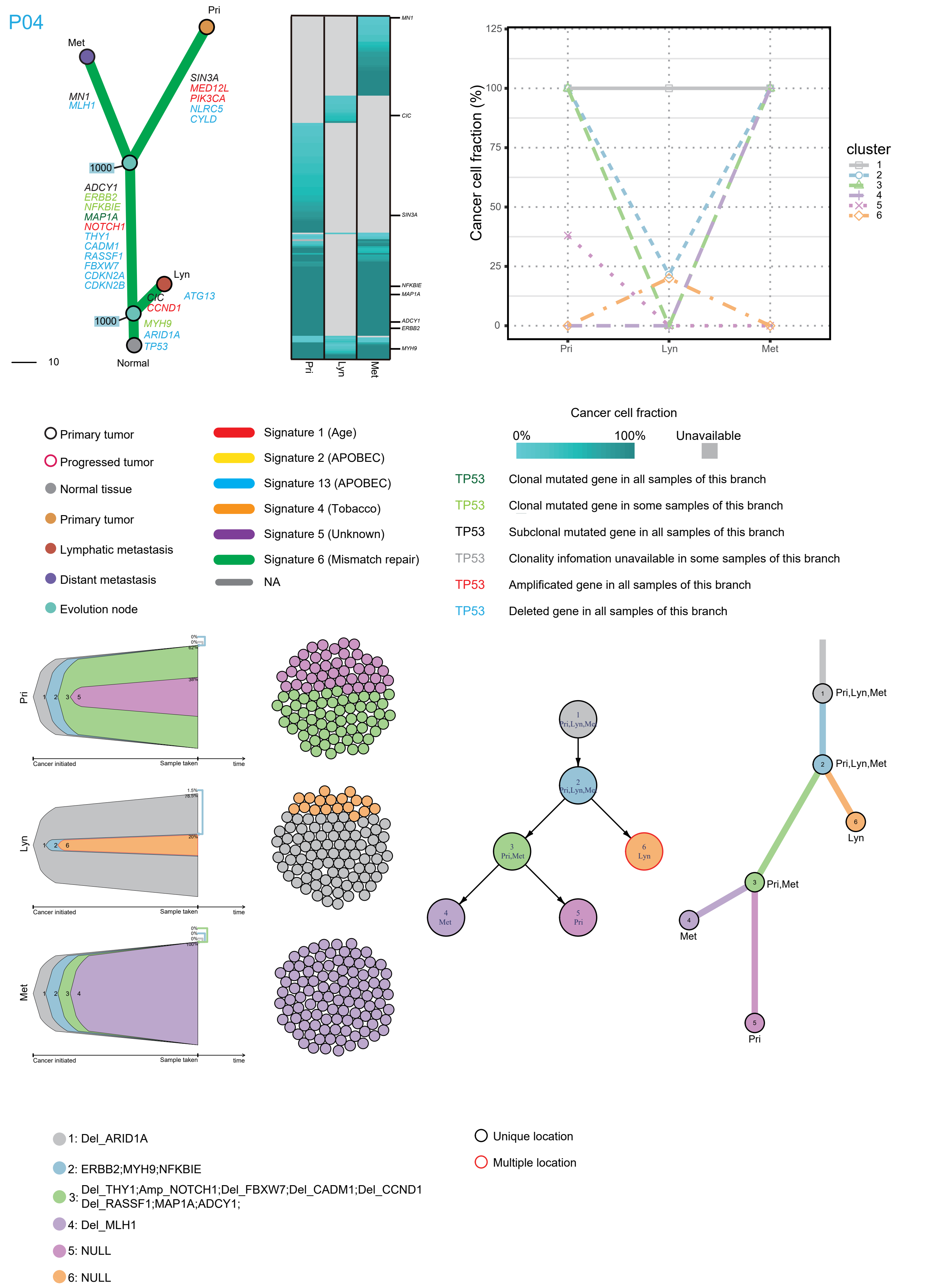

P04
Pri
Met
MN1
MLH1
SIN3A
MED12L
PIK3CA
NLRC5
CYLD
1000
ADCY1
ERBB2
NFKBIE
MAP1A
NOTCH1
THY1
CADM1
RASSF1
FBXW7
CDKN2A
CDKN2B
Lyn
CIC
CCND1
ATG13
1000
MYH9
ARID1A
TP53
10
Normal
MN1
CIC
SIN3A
NFKBIE
MAP1A
ADCY1
ERBB2
MYH9
Pri
Lyn
Met
Cancer cell fraction (%)
125
100
75
50
25
0
Pri
Lyn
Met
cluster
1
2
3
4
5
6
Primary tumor
Progressed tumor
Normal tissue
Primary tumor
Lymphatic metastasis
Distant metastasis
Evolution node
Signature 1 (Age)
Signature 2 (APOBEC)
Signature 13 (APOBEC)
Signature 4 (Tobacco)
Signature 5 (Unknown)
Signature 6 (Mismatch repair)
NA
Cancer cell fraction
0%
100%
Unavailable
TP53 Clonal mutated gene in all samples of this branch
TP53 Clonal mutated gene in some samples of this branch
TP53 Subclonal mutated gene in all samples of this branch
TP53 Clonality infomation unavailable in some samples of this branch
TP53 Amplificated gene in all samples of this branch
TP53 Deleted gene in all samples of this branch
Pri
Cancer initiated
Sample taken
time
Lyn
Cancer initiated
Sample taken
time
Met
Cancer initiated
Sample taken
time
1 Pri,Lyn,Met
2 Pri,Lyn,Met
3 Pri,Met
6 Lyn
4 Met
5 Pri
1 Pri,Lyn,Met
2 Pri,Lyn,Met
6 Lyn
3 Pri,Met
4 Met
5 Pri
1: Del_ARID1A
2: ERBB2;MYH9;NFKBIE
3: Del_THY1;Amp_NOTCH1;Del_FBXW7;Del_CADM1;Del_CCND1
Del_RASSF1;MAP1A;ADCY1;
4: Del_MLH1
5: NULL
6: NULL
Unique location
Multiple location

e

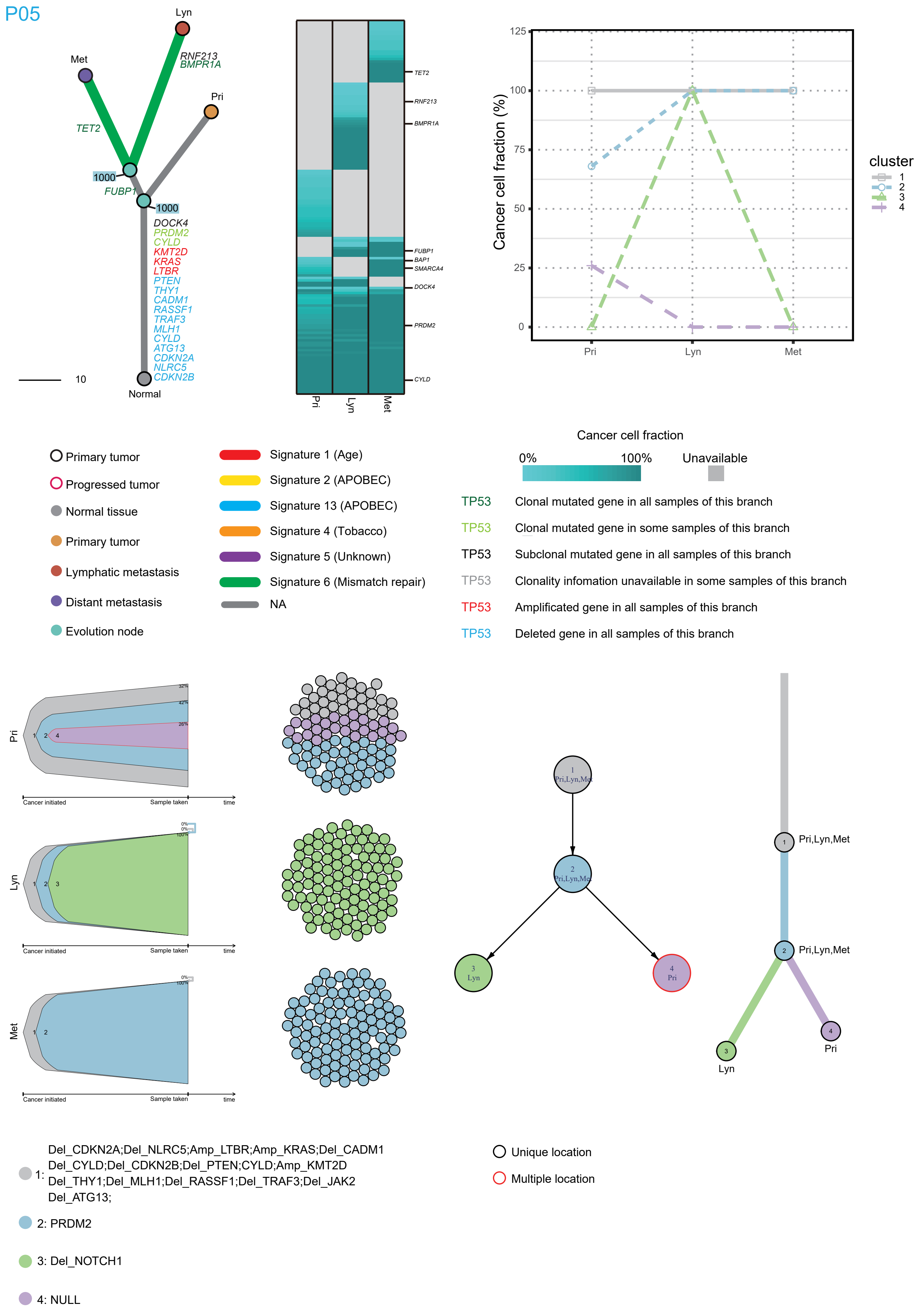
P05
Lyn
Met
Pri
RNF213
BMPR1A
TET2
1000
FUBP1
1000
DOCK4
PRDM2
CYLD
KMT2D
KRAS
LTBR
PTEN
THY1
CADM1
RASSF1
TRAF3
MLH1
CYLD
ATG13
CDKN2A
NLRC5
CDKN2B
10
Normal
TET2
RNF213
BMPR1A
FUBP1
BAP1
SMARCA4
DOCK4
PRDM2
CYLD
Pri
Lyn
Met
125
100
75
50
25
0
Cancer cell fraction (%)
Pri
Lyn
Met
cluster
1
2
3
4
Primary tumor
Progressed tumor
Normal tissue
Primary tumor
Lymphatic metastasis
Distant metastasis
Evolution node
Signature 1 (Age)
Signature 2 (APOBEC)
Signature 13 (APOBEC)
Signature 4 (Tobacco)
Signature 5 (Unknown)
Signature 6 (Mismatch repair)
NA
Cancer cell fraction
0%
100%
Unavailable
TP53 Clonal mutated gene in all samples of this branch
TP53 Clonal mutated gene in some samples of this branch
TP53 Subclonal mutated gene in all samples of this branch
TP53 Clonality infomation unavailable in some samples of this branch
TP53 Amplificated gene in all samples of this branch
TP53 Deleted gene in all samples of this branch
Pri
Cancer initiated
Sample taken
time
Lyn
Cancer initiated
Sample taken
time
Met
Cancer initiated
Sample taken
time
1
Pri,Lyn,Met
2
Pri,Lyn,Met
3
Lyn
4
Pri
Pri,Lyn,Met
Pri,Lyn,Met
Lyn
Pri
1: Del_CDKN2A;Del_NLRC5;Amp_LTBR;Amp_KRAS;Del_CADM1
Del_CYLD;Del_CDKN2B;Del_PTEN;CYLD;Amp_KMT2D
Del_THY1;Del_MLH1;Del_RASSF1;Del_TRAF3;Del_JAK2
Del_ATG13;
2: PRDM2
3: Del_NOTCH1
4: NULL
Unique location
Multiple location

f

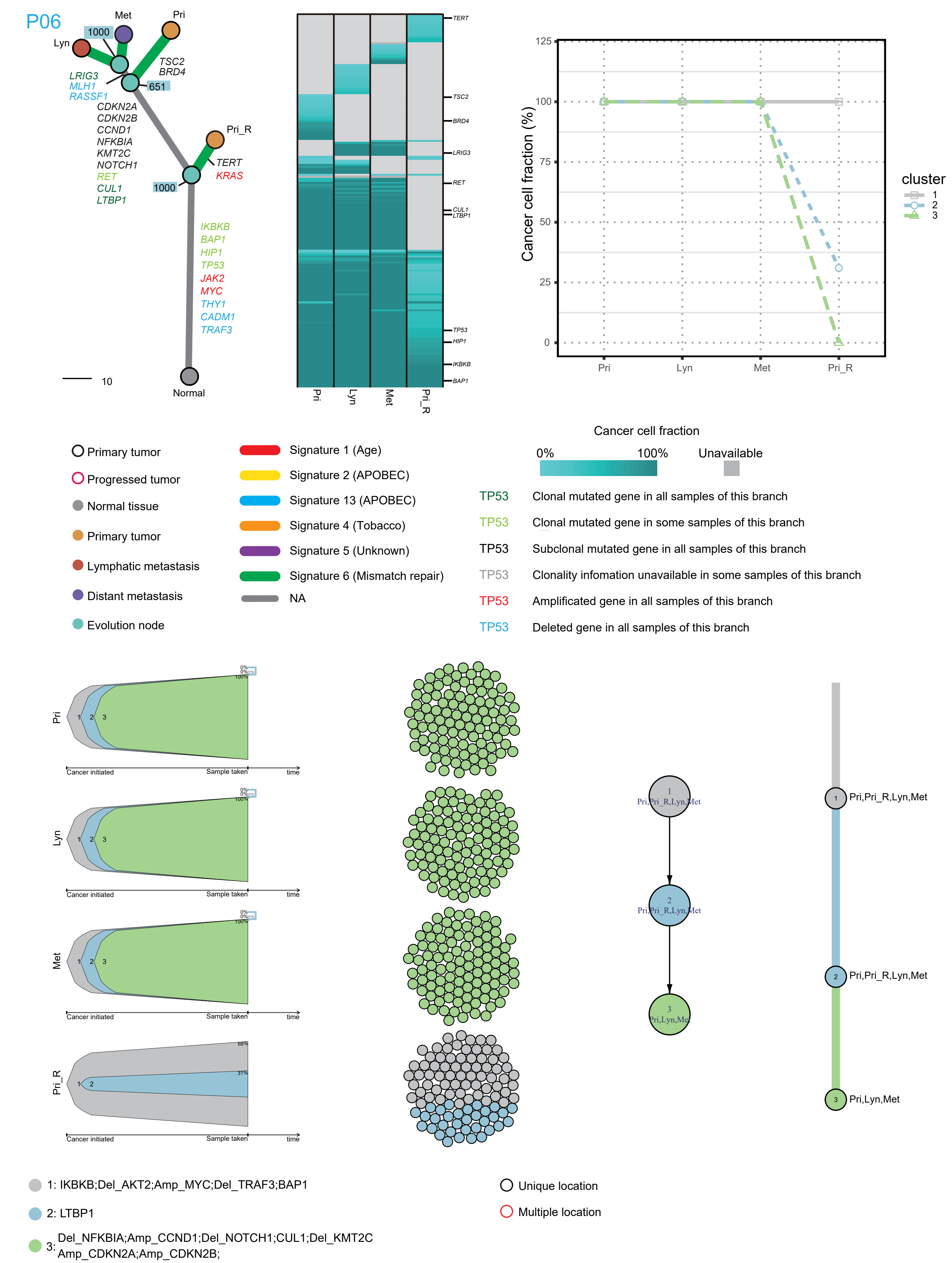
P06
Met
Pri
Lyn
1000
651
TSC2
BRD4
LRIG3
MLH1
RASSF1
CDKN2A
CDKN2B
CCND1
NFKBIA
KMT2C
NOTCH1
RET
CUL1
LTBP1
Pri_R
TERT
KRAS
1000
IKBKB
BAP1
HIP1
TP53
JAK2
MYC
THY1
CADM1
TRAF3
10
Normal
TERT
TSC2
BRD4
LRIG3
RET
CUL1
LTBP1
TP53
HIP1
IKBKB
BAP1
Pri
Lyn
Met
Pri_R
Cancer cell fraction (%)
125
100
75
50
25
0
Pri
Lyn
Met
Pri_R
cluster
1
2
3
Primary tumor
Progressed tumor
Normal tissue
Primary tumor
Lymphatic metastasis
Distant metastasis
Evolution node
Signature 1 (Age)
Signature 2 (APOBEC)
Signature 13 (APOBEC)
Signature 4 (Tobacco)
Signature 5 (Unknown)
Signature 6 (Mismatch repair)
NA
Cancer cell fraction
0%
100%
Unavailable
TP53 Clonal mutated gene in all samples of this branch
TP53 Clonal mutated gene in some samples of this branch
TP53 Subclonal mutated gene in all samples of this branch
TP53 Clonality infomation unavailable in some samples of this branch
TP53 Amplificated gene in all samples of this branch
TP53 Deleted gene in all samples of this branch
Pri
Lyn
Met
Pri_R
Cancer initiated
Sample taken
time
1
2
3
69%
31%
1
Pri,Pri_R,Lyn,Met
2
Pri,Pri_R,Lyn,Met
3
Pri,Lyn,Met
1 Pri,Pri_R,Lyn,Met
2 Pri,Pri_R,Lyn,Met
3 Pri,Lyn,Met
1: IKBKB;Del_AKT2;Amp_MYC;Del_TRAF3;BAP1
2: LTBP1
3: Del_NFKBIA;Amp_CCND1;Del_NOTCH1;CUL1;Del_KMT2C
Amp_CDKN2A;Amp_CDKN2B;
Unique location
Multiple location

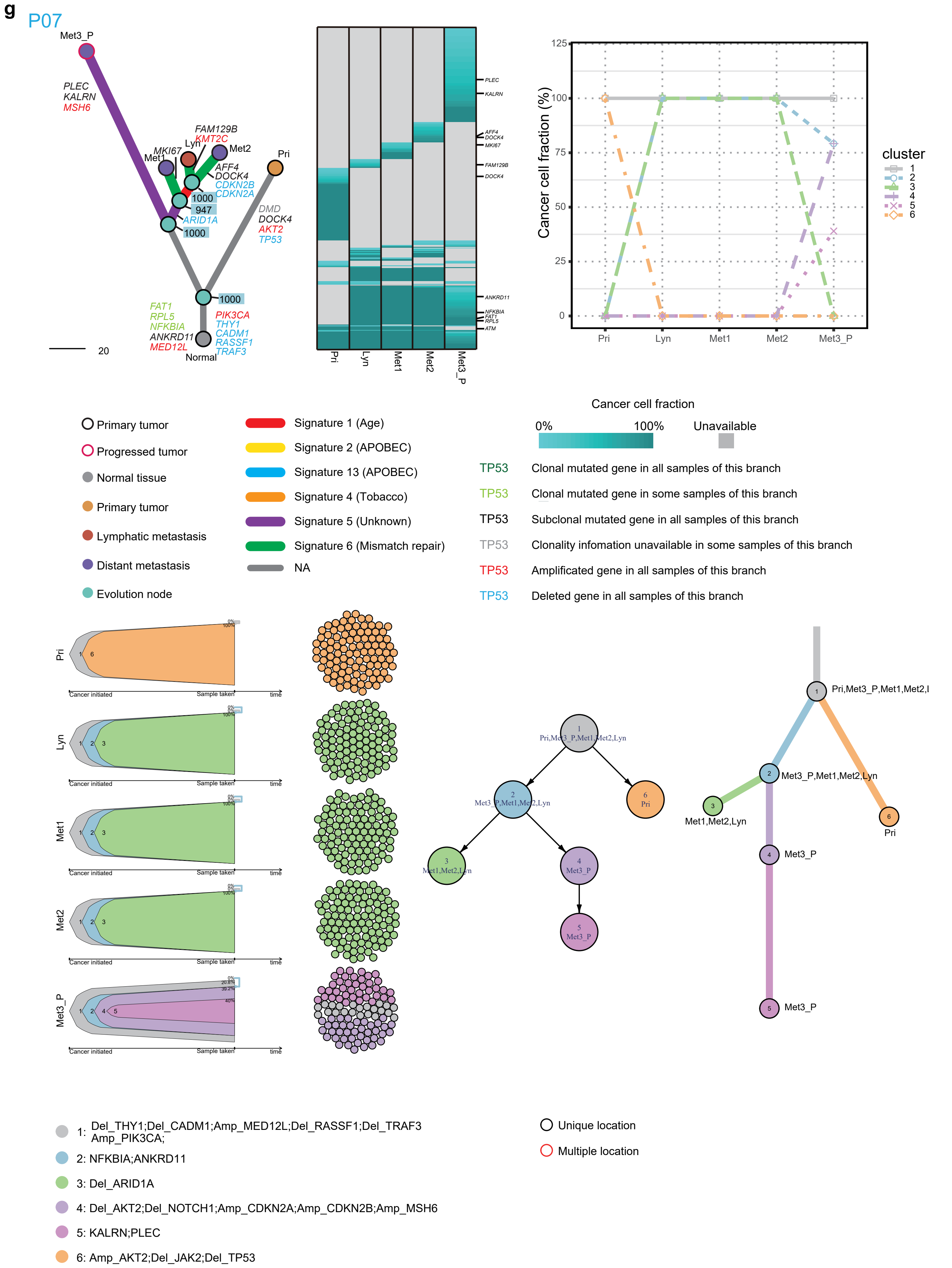
g
P07
Met3_P
PLEC
KALRN
MSH6
FAM129B
KMT2C
Lyn
MKI67
Met2
Met1
AFF4
DOCK4
CDKN2B
CDKN2A
1000
947
ARID1A
1000
Pri
DMD
DOCK4
AKT2
TP53
1000
FAT1
RPL5
NFKBIA
ANKRD11
MED12L
PIK3CA
THY1
CADM1
RASSF1
TRAF3
Normal
20
Pri
Lyn
Met1
Met2
Met3_P
PLEC
KALRN
AFF4
DOCK4
MKI67
FAM129B
DOCK4
ANKRD11
NFKBIA
FAT1
RPL5
ATM
Cancer cell fraction (%)
125
100
75
50
25
0
cluster
1
2
3
4
5
6
Primary tumor
Progressed tumor
Normal tissue
Primary tumor
Lymphatic metastasis
Distant metastasis
Evolution node
Signature 1 (Age)
Signature 2 (APOBEC)
Signature 13 (APOBEC)
Signature 4 (Tobacco)
Signature 5 (Unknown)
Signature 6 (Mismatch repair)
NA
Cancer cell fraction
0%
100%
Unavailable
TP53 Clonal mutated gene in all samples of this branch
TP53 Clonal mutated gene in some samples of this branch
TP53 Subclonal mutated gene in all samples of this branch
TP53 Clonality infomation unavailable in some samples of this branch
TP53 Amplificated gene in all samples of this branch
TP53 Deleted gene in all samples of this branch
Cancer initiated
Sample taken
time
Pri,Met3_P,Met1,Met2,Lyn
Met3_P,Met1,Met2,Lyn
Met1,Met2,Lyn
Met3_P
Pri
1: Del_THY1;Del_CADM1;Amp_MED12L;Del_RASSF1;Del_TRAF3
Amp_PIK3CA;
2: NFKBIA;ANKRD11
3: Del_ARID1A
4: Del_AKT2;Del_NOTCH1;Amp_CDKN2A;Amp_CDKN2B;Amp_MSH6
5: KALRN;PLEC
6: Amp_AKT2;Del_JAK2;Del_TP53
Unique location
Multiple location

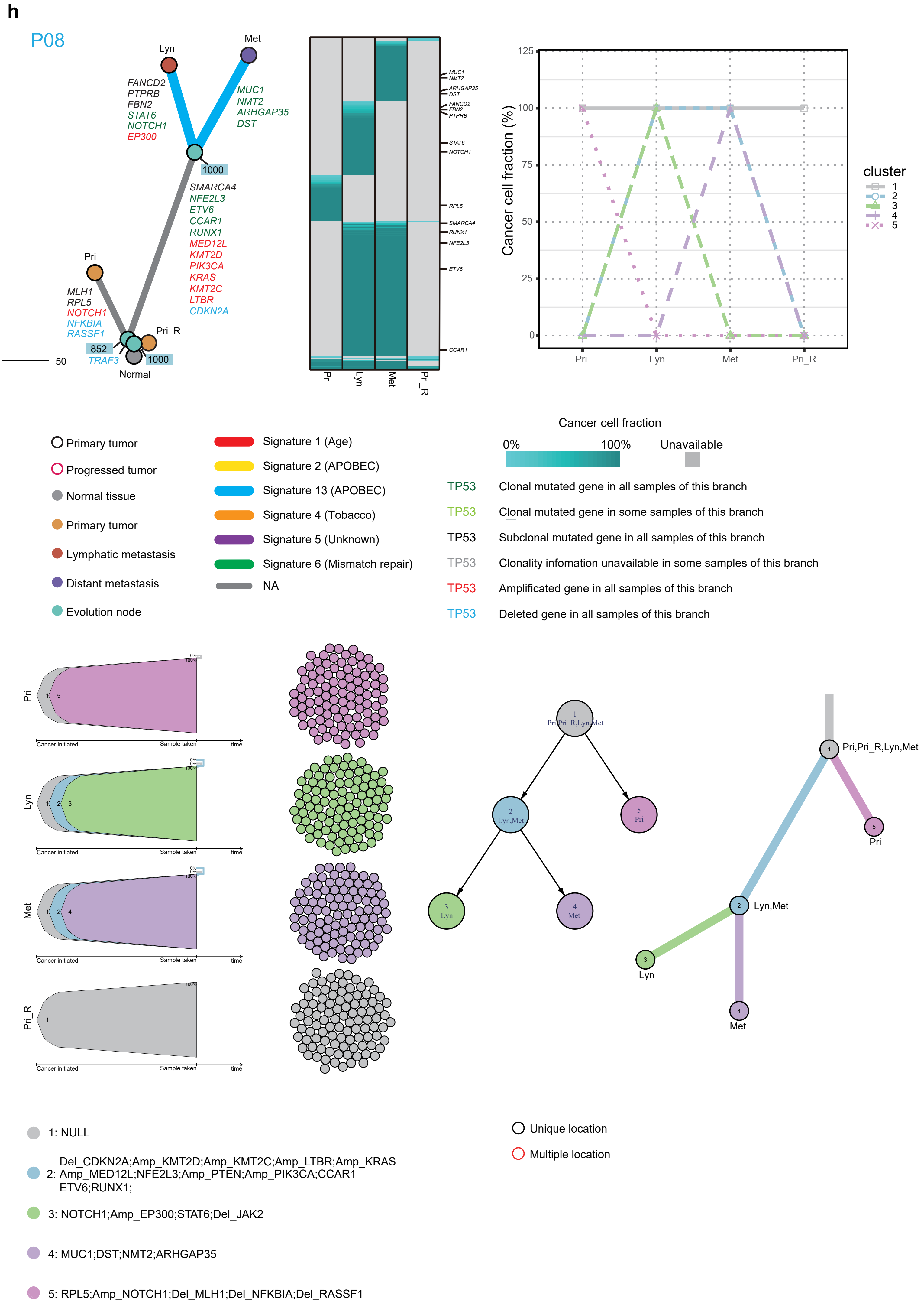

h
P08
Lyn
Met
FANCD2
PTPRB
FBN2
STAT6
NOTCH1
EP300
MUC1
NMT2
ARHGAP35
DST
1000
SMARCA4
NFE2L3
ETV6
CCAR1
RUNX1
MED12L
KMT2D
PIK3CA
KRAS
KMT2C
LTBR
CDKN2A
Pri
MLH1
RPL5
NOTCH1
NFKBIA
RASSF1
852
TRAF3
1000
Pri_R
Normal
50
Pri
Lyn
Met
Pri_R
Cancer cell fraction (%)
cluster
1
2
3
4
5
Primary tumor
Progressed tumor
Normal tissue
Primary tumor
Lymphatic metastasis
Distant metastasis
Evolution node
Signature 1 (Age)
Signature 2 (APOBEC)
Signature 13 (APOBEC)
Signature 4 (Tobacco)
Signature 5 (Unknown)
Signature 6 (Mismatch repair)
NA
Cancer cell fraction
0%
100%
Unavailable
TP53 Clonal mutated gene in all samples of this branch
TP53 Clonal mutated gene in some samples of this branch
TP53 Subclonal mutated gene in all samples of this branch
TP53 Clonality infomation unavailable in some samples of this branch
TP53 Amplificated gene in all samples of this branch
TP53 Deleted gene in all samples of this branch
Cancer initiated
Sample taken
time
Pri,Pri_R,Lyn,Met
Lyn,Met
Pri
Lyn
Met
1: NULL
2: Del_CDKN2A;Amp_KMT2D;Amp_KMT2C;Amp_LTBR;Amp_KRAS Amp_MED12L;NFE2L3;Amp_PTEN;Amp_PIK3CA;CCAR1 ETV6;RUNX1;
3: NOTCH1;Amp_EP300;STAT6;Del_JAK2
4: MUC1;DST;NMT2;ARHGAP35
5: RPL5;Amp_NOTCH1;Del_MLH1;Del_NFKBIA;Del_RASSF1
Unique location
Multiple location

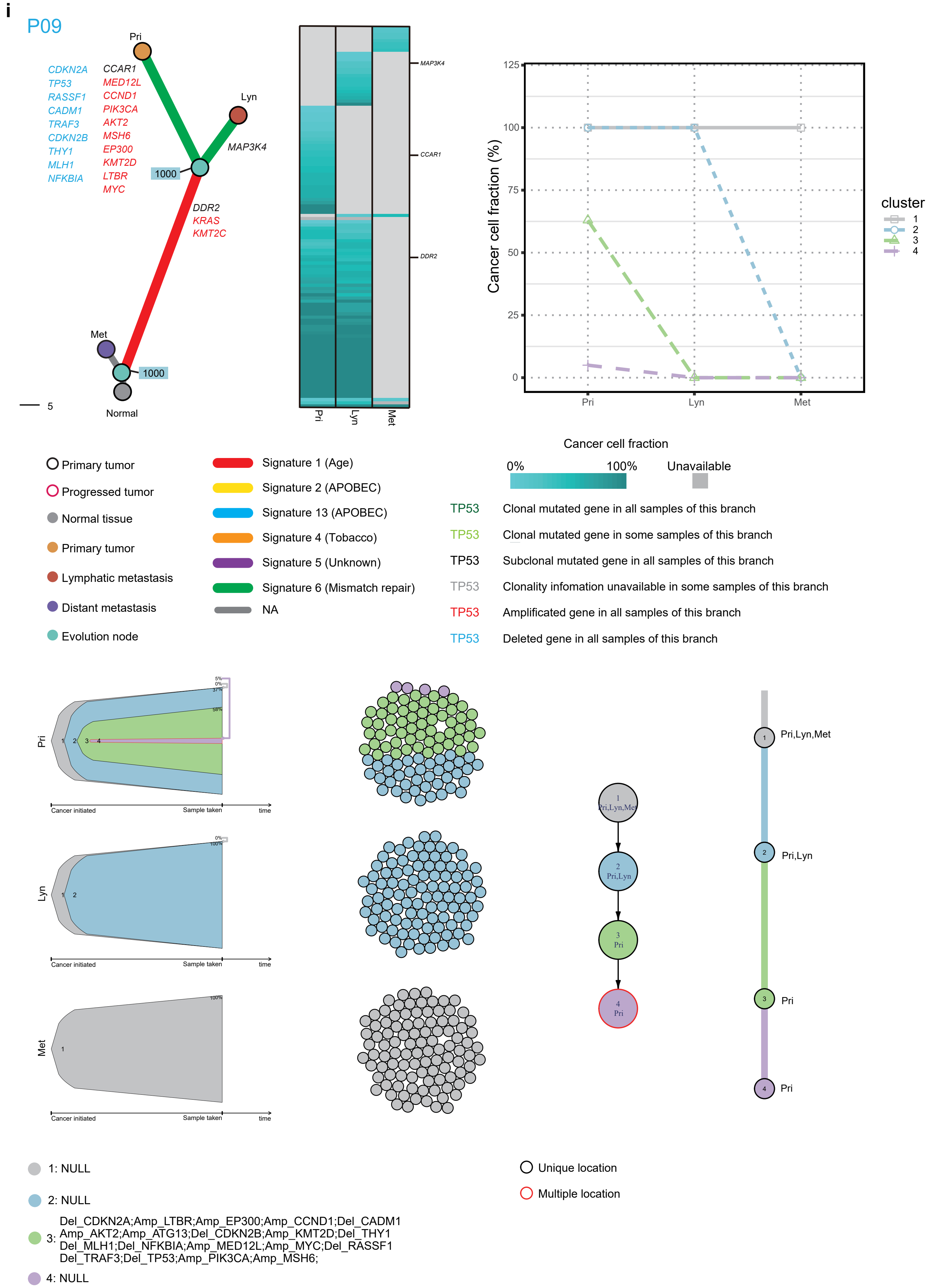

i
P09
Pri
Lyn
Met
Normal
CDKN2A
TP53
RASSF1
CADM1
TRAF3
CDKN2B
THY1
MLH1
NFKBIA
CCAR1
MED12L
CCND1
PIK3CA
AKT2
MSH6
EP300
KMT2D
LTBR
MYC
MAP3K4
1000
DDR2
KRAS
KMT2C
5
MAP3K4
CCAR1
DDR2
Cancer cell fraction (%)
cluster
1
2
3
4
Cancer cell fraction
0%
100%
Unavailable
Primary tumor
Progressed tumor
Normal tissue
Primary tumor
Lymphatic metastasis
Distant metastasis
Evolution node
Signature 1 (Age)
Signature 2 (APOBEC)
Signature 13 (APOBEC)
Signature 4 (Tobacco)
Signature 5 (Unknown)
Signature 6 (Mismatch repair)
NA
TP53 Clonal mutated gene in all samples of this branch
TP53 Clonal mutated gene in some samples of this branch
TP53 Subclonal mutated gene in all samples of this branch
TP53 Clonality infomation unavailable in some samples of this branch
TP53 Amplificated gene in all samples of this branch
TP53 Deleted gene in all samples of this branch
Cancer initiated
Sample taken
time
1 Pri,Lyn,Met
2 Pri,Lyn
3 Pri
4 Pri
1: NULL
2: NULL
3: Del_CDKN2A;Amp_LTBR;Amp_EP300;Amp_CCND1;Del_CADM1 Amp_AKT2;Amp_ATG13;Del_CDKN2B;Amp_KMT2D;Del_THY1 Del_MLH1;Del_NFKBIA;Amp_MED12L;Amp_MYC;Del_RASSF1 Del_TRAF3;Del_TP53;Amp_PIK3CA;Amp_MSH6;
4: NULL
Unique location
Multiple location

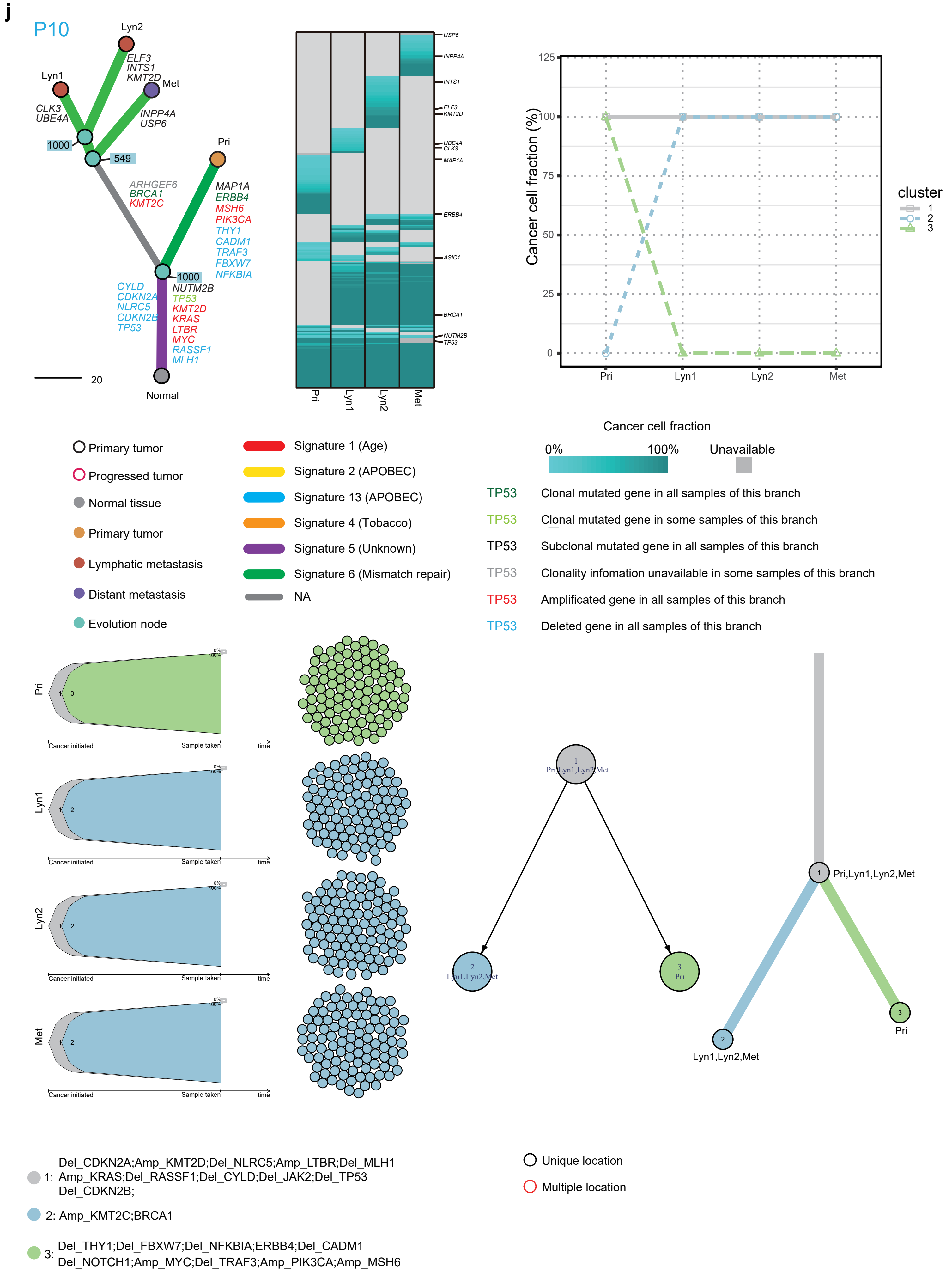
j
P10
Lyn2
Lyn1
Met
Pri
Normal
ELF3
INTS1
KMT2D
INPP4A
USP6
CLK3
UBE4A
1000
549
ARHGEF6
BRCA1
KMT2C
MAP1A
ERBB4
MSH6
PIK3CA
THY1
CADM1
TRAF3
FBXW7
NFKBIA
1000
CYLD
CDKN2A
NLRC5
CDKN2B
TP53
NUTM2B
TP53
KMT2D
KRAS
LTBR
MYC
RASSF1
MLH1
20
USP6
INPP4A
INTS1
ELF3
KMT2D
UBE4A
CLK3
MAP1A
ERBB4
ASIC1
BRCA1
NUTM2B
TP53
Pri
Lyn1
Lyn2
Met
Cancer cell fraction (%)
125
100
75
50
25
0
Pri
Lyn1
Lyn2
Met
cluster
1
2
3
Cancer cell fraction
0%
100%
Unavailable
Primary tumor
Progressed tumor
Normal tissue
Primary tumor
Lymphatic metastasis
Distant metastasis
Evolution node
Signature 1 (Age)
Signature 2 (APOBEC)
Signature 13 (APOBEC)
Signature 4 (Tobacco)
Signature 5 (Unknown)
Signature 6 (Mismatch repair)
NA
TP53 Clonal mutated gene in all samples of this branch
TP53 Clonal mutated gene in some samples of this branch
TP53 Subclonal mutated gene in all samples of this branch
TP53 Clonality infomation unavailable in some samples of this branch
TP53 Amplificated gene in all samples of this branch
TP53 Deleted gene in all samples of this branch
Pri
Lyn1
Lyn2
Met
Cancer initiated
Sample taken
time
1
Pri,Lyn1,Lyn2,Met
2
Lyn1,Lyn2,Met
3
Pri
Pri,Lyn1,Lyn2,Met
Lyn1,Lyn2,Met
Pri
1: Del_CDKN2A;Amp_KMT2D;Del_NLRC5;Amp_LTBR;Del_MLH1
Amp_KRAS;Del_RASSF1;Del_CYLD;Del_JAK2;Del_TP53
Del_CDKN2B;
2: Amp_KMT2C;BRCA1
3: Del_THY1;Del_FBXW7;Del_NFKBIA;ERBB4;Del_CADM1
Del_NOTCH1;Amp_MYC;Del_TRAF3;Amp_PIK3CA;Amp_MSH6
Unique location
Multiple location

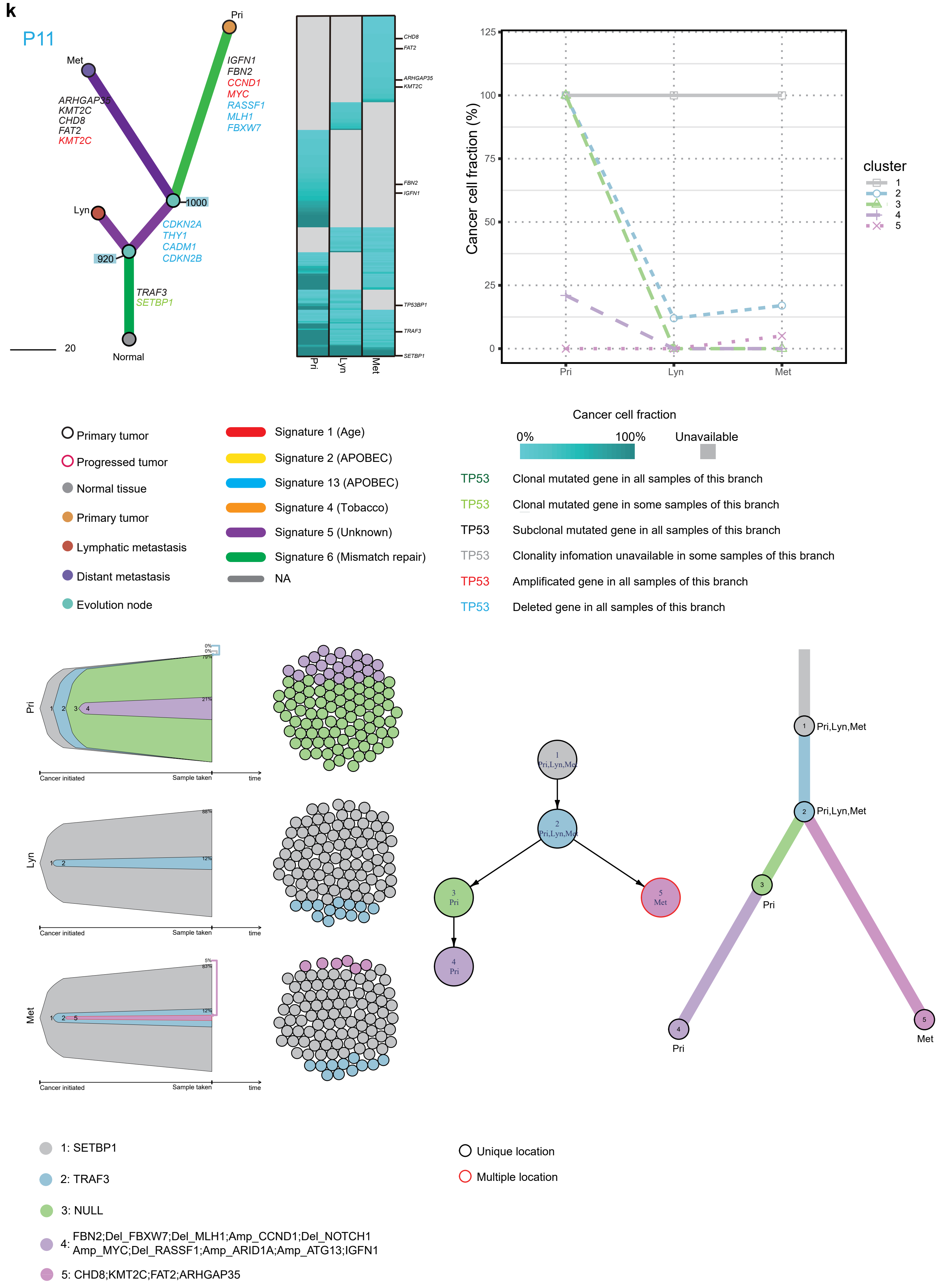
k
P11
Pri
Met
Lyn
Normal
IGFN1
FBN2
CCND1
MYC
RASSF1
MLH1
FBXW7
ARHGAP35
KMT2C
CHD8
FAT2
KMT2C
1000
920
CDKN2A
THY1
CADM1
CDKN2B
TRAF3
SETBP1
20
CHD8
FAT2
ARHGAP35
KMT2C
FBN2
IGFN1
TP53BP1
TRAF3
SETBP1
Pri
Lyn
Met
Cancer cell fraction (%)
125
100
75
50
25
0
cluster
1
2
3
4
5
Primary tumor
Progressed tumor
Normal tissue
Primary tumor
Lymphatic metastasis
Distant metastasis
Evolution node
Signature 1 (Age)
Signature 2 (APOBEC)
Signature 13 (APOBEC)
Signature 4 (Tobacco)
Signature 5 (Unknown)
Signature 6 (Mismatch repair)
NA
Cancer cell fraction
0%
100%
Unavailable
TP53 Clonal mutated gene in all samples of this branch
TP53 Clonal mutated gene in some samples of this branch
TP53 Subclonal mutated gene in all samples of this branch
TP53 Clonality infomation unavailable in some samples of this branch
TP53 Amplificated gene in all samples of this branch
TP53 Deleted gene in all samples of this branch
Cancer initiated
Sample taken
time
Pri,Lyn,Met
Unique location
Multiple location
1: SETBP1
2: TRAF3
3: NULL
4: FBN2;Del_FBXW7;Del_MLH1;Amp_CCND1;Del_NOTCH1
Amp_MYC;Del_RASSF1;Amp_ARID1A;Amp_ATG13;IGFN1
5: CHD8;KMT2C;FAT2;ARHGAP35

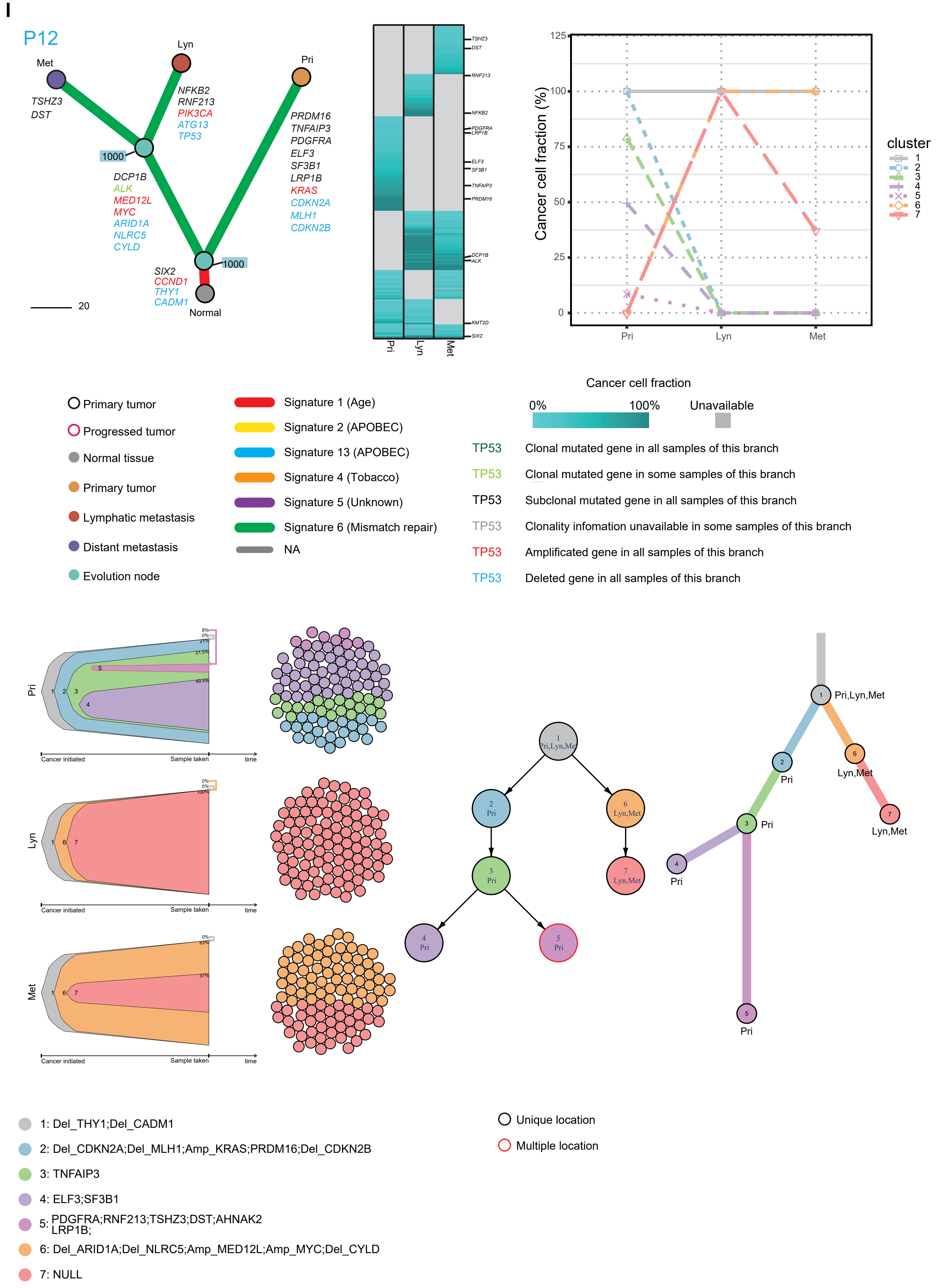
I
P12
Met
Lyn
Pri
TSHZ3
DST
NFKB2
RNF213
PIK3CA
ATG13
TP53
1000
DCP1B
ALK
MED12L
MYC
ARID1A
NLRC5
CYLD
PRDM16
TNFAIP3
PDGFRA
ELF3
SF3B1
LRP1B
KRAS
CDKN2A
MLH1
CDKN2B
SIX2
CCND1
THY1
CADM1
1000
Normal
20
Pri
Lyn
Met
Cancer cell fraction (%)
cluster
Cancer cell fraction
0%
100%
Unavailable
Primary tumor
Progressed tumor
Normal tissue
Primary tumor
Lymphatic metastasis
Distant metastasis
Evolution node
Signature 1 (Age)
Signature 2 (APOBEC)
Signature 13 (APOBEC)
Signature 4 (Tobacco)
Signature 5 (Unknown)
Signature 6 (Mismatch repair)
NA
TP53 Clonal mutated gene in all samples of this branch
TP53 Clonal mutated gene in some samples of this branch
TP53 Subclonal mutated gene in all samples of this branch
TP53 Clonality infomation unavailable in some samples of this branch
TP53 Amplificated gene in all samples of this branch
TP53 Deleted gene in all samples of this branch
Cancer initiated
Sample taken
time
Pri,Lyn,Met
Lyn,Met
Unique location
Multiple location
1: Del_THY1;Del_CADM1
2: Del_CDKN2A;Del_MLH1;Amp_KRAS;PRDM16;Del_CDKN2B
3: TNFAIP3
4: ELF3;SF3B1
5: PDGFRA;RNF213;TSHZ3;DST;AHNAK2 LRP1B;
6: Del_ARID1A;Del_NLRC5;Amp_MED12L;Amp_MYC;Del_CYLD
7: NULL

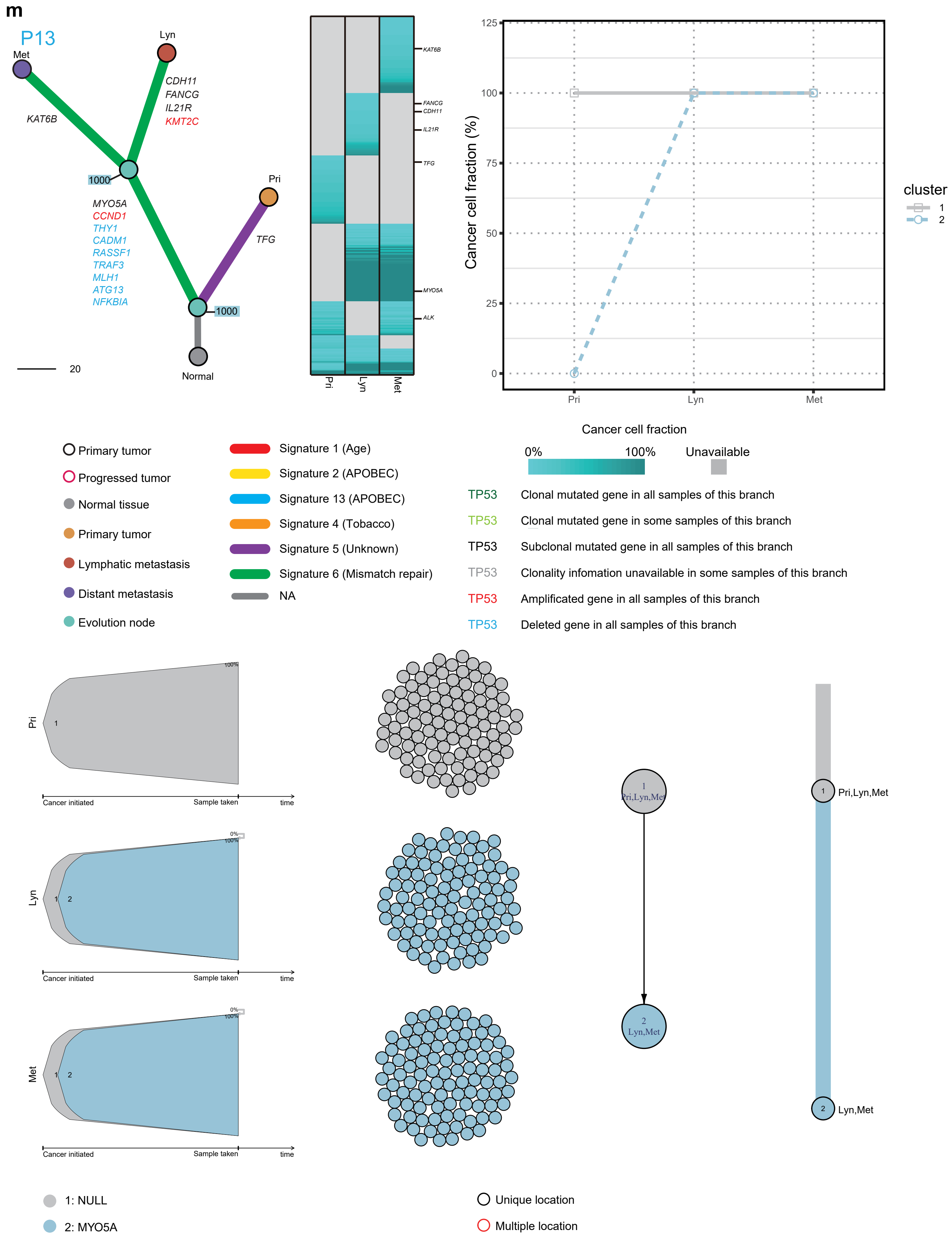
m
P13
Met
Lyn
Pri
Normal
KAT6B
CDH11
FANCG
IL21R
KMT2C
MYO5A
CCND1
THY1
CADM1
RASSF1
TRAF3
MLH1
ATG13
NFKBIA
TFG
1000
1000
20
KAT6B
FANCG
CDH11
IL21R
TFG
MYO5A
ALK
Pri
Lyn
Met
Cancer cell fraction (%)
125
100
75
50
25
0
cluster
1
2
Cancer cell fraction
0%
100%
Unavailable
Primary tumor
Progressed tumor
Normal tissue
Primary tumor
Lymphatic metastasis
Distant metastasis
Evolution node
Signature 1 (Age)
Signature 2 (APOBEC)
Signature 13 (APOBEC)
Signature 4 (Tobacco)
Signature 5 (Unknown)
Signature 6 (Mismatch repair)
NA
TP53 Clonal mutated gene in all samples of this branch
TP53 Clonal mutated gene in some samples of this branch
TP53 Subclonal mutated gene in all samples of this branch
TP53 Clonality infomation unavailable in some samples of this branch
TP53 Amplificated gene in all samples of this branch
TP53 Deleted gene in all samples of this branch
Cancer initiated
Sample taken
time
1
2
1
Pri,Lyn,Met
2
Lyn,Met
Pri,Lyn,Met
Lyn,Met
1: NULL
2: MYO5A
Unique location
Multiple location

n

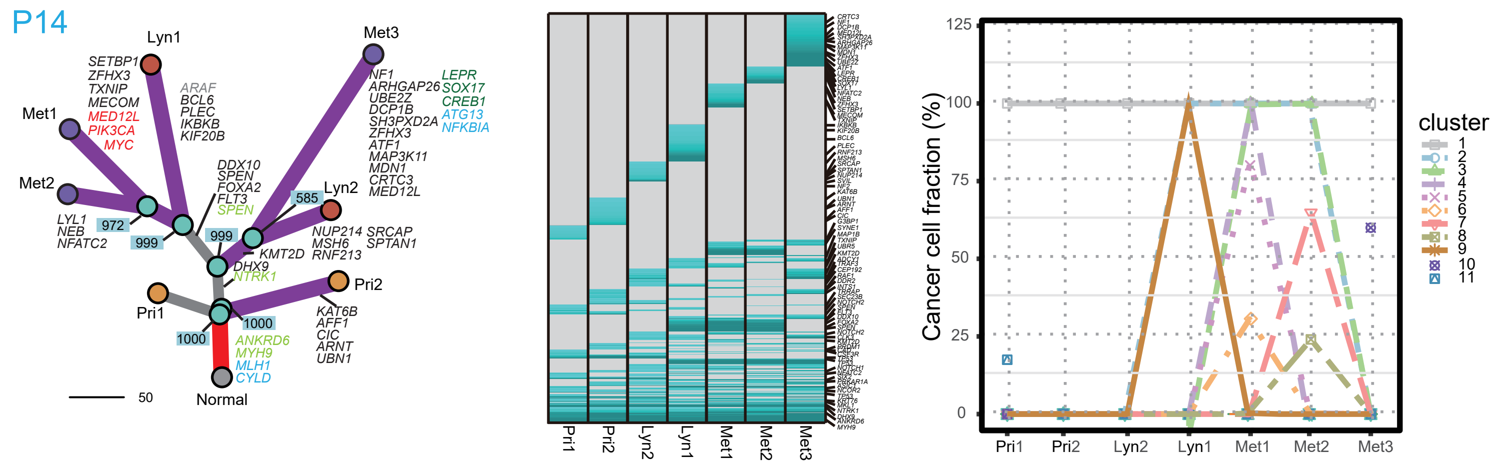

Cancer cell fraction

0% 100% Unavailable

Primary tumor

Progressed tumor

Normal tissue

Primary tumor

Lymphatic metastasis

Distant metastasis

Evolution node

Signature 1 (Age)

Signature 2 (APOBEC)

Signature 13 (APOBEC)

Signature 4 (Tobacco)

Signature 5 (Unknown)

Signature 6 (Mismatch repair)

NA

TP53 Clonal mutated gene in all samples of this branch

TP53 Clonal mutated gene in some samples of this branch

TP53 Subclonal mutated gene in all samples of this branch

TP53 Clonality infomation unavailable in some samples of this branch

TP53 Amplificated gene in all samples of this branch

TP53 Deleted gene in all samples of this branch

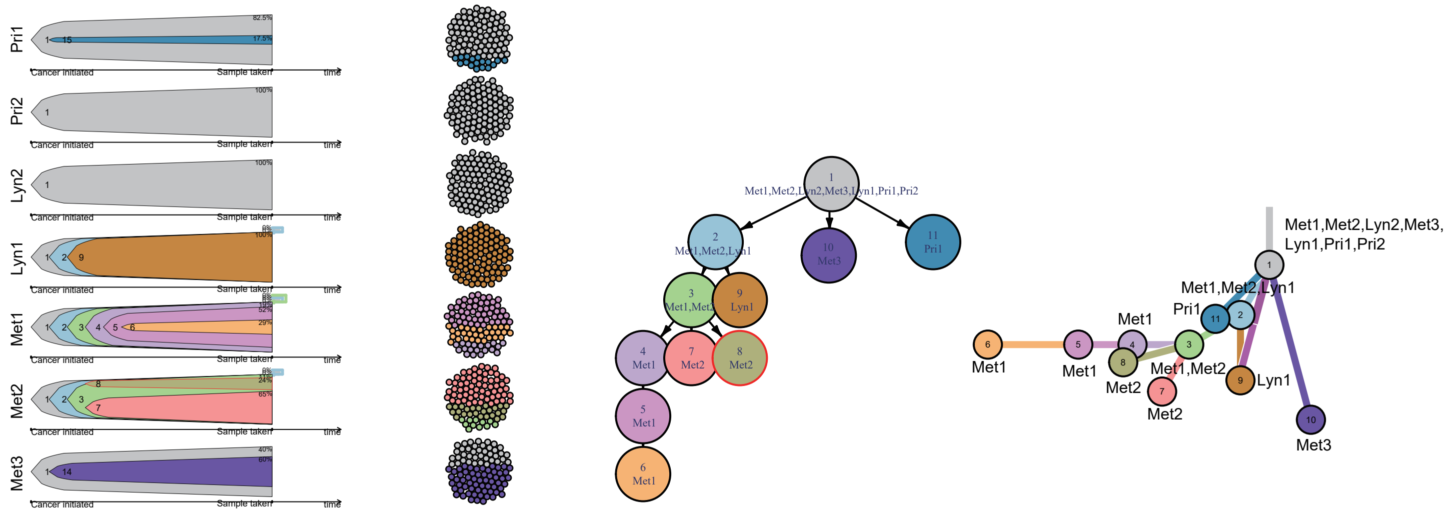

1: NULL

2: SPEN

3: NULL

4: Amp_MED12L;Amp_MYC;Del_EP300;Amp_PIK3CA

5: NULL

6: SETBP1;MECOM

7: NULL

8: LYL1

9: NULL

10: ATF1;ZFHX3;CRTC3;CAD;UBE2Z ARHGAP26;MED12L;Del_NFKBIA;CREB1;SH3PXD2A SOX17;Del_CCND1;NF1;Del_ATG13;MAP3K11 MDN1;

11: NULL

Unique location

Multiple location

o

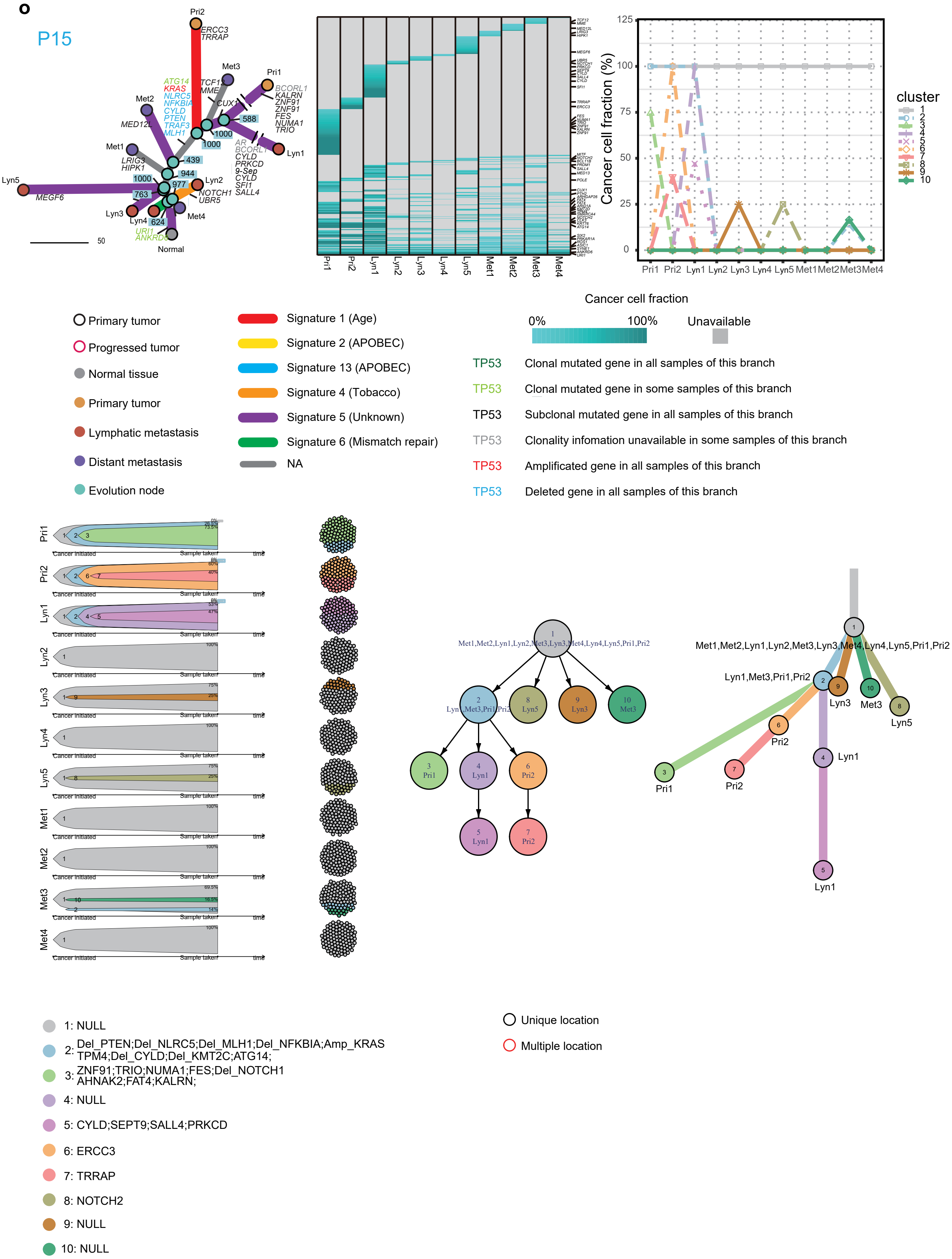
P15
Pri2
ERCC3
TRRAP
Met3
Pri1
ATG14
KRAS
NLRC5
NFKBIA
CYLD
PTEN
TRAF3
MLH1
TCF12
MME
CUX1
BCORL1
KALRN
ZNF91
ZNF91
FES
NUMA1
TRIO
Met2
MED12L
588
1000
1000
AR
BCORL1
CYLD
PRKCD
9-Sep
CYLD
SFI1
SALL4
Lyn1
Met1
LRIG3
HIPK1
439
944
Lyn5
MEGF6
1000
977
Lyn2
763
NOTCH1
UBR5
Lyn3
Lyn4
624
Met4
URI1
ANKRD6
Normal
50
Pri1 Pri2 Lyn1 Lyn2 Lyn3 Lyn4 Lyn5 Met1 Met2 Met3 Met4
Cancer cell fraction (%)
125
100
75
50
25
0
cluster
1
2
3
4
5
6
7
8
9
10
Cancer cell fraction
0%
100%
Unavailable
Primary tumor
Progressed tumor
Normal tissue
Primary tumor
Lymphatic metastasis
Distant metastasis
Evolution node
Signature 1 (Age)
Signature 2 (APOBEC)
Signature 13 (APOBEC)
Signature 4 (Tobacco)
Signature 5 (Unknown)
Signature 6 (Mismatch repair)
NA
TP53 Clonal mutated gene in all samples of this branch
TP53 Clonal mutated gene in some samples of this branch
TP53 Subclonal mutated gene in all samples of this branch
TP53 Clonality infomation unavailable in some samples of this branch
TP53 Amplificated gene in all samples of this branch
TP53 Deleted gene in all samples of this branch
Pri1
Pri2
Lyn1
Lyn2
Lyn3
Lyn4
Lyn5
Met1
Met2
Met3
Met4
Cancer initiated
Sample taken
time
Met1,Met2,Lyn1,Lyn2,Met3,Lyn3,Met4,Lyn4,Lyn5,Pri1,Pri2
Lyn1,Met3,Pri1,Pri2
Lyn3
Met3
Lyn5
Pri2
Lyn1
Pri1
Pri2
Lyn1
1: NULL
2: Del_PTEN;Del_NLRC5;Del_MLH1;Del_NFKBIA;Amp_KRAS
TPM4;Del_CYLD;Del_KMT2C;ATG14;
3: ZNF91;TRIO;NUMA1;FES;Del_NOTCH1
AHNAK2;FAT4;KALRN;
4: NULL
5: CYLD;SEPT9;SALL4;PRKCD
6: ERCC3
7: TRRAP
8: NOTCH2
9: NULL
10: NULL
Unique location
Multiple location

p

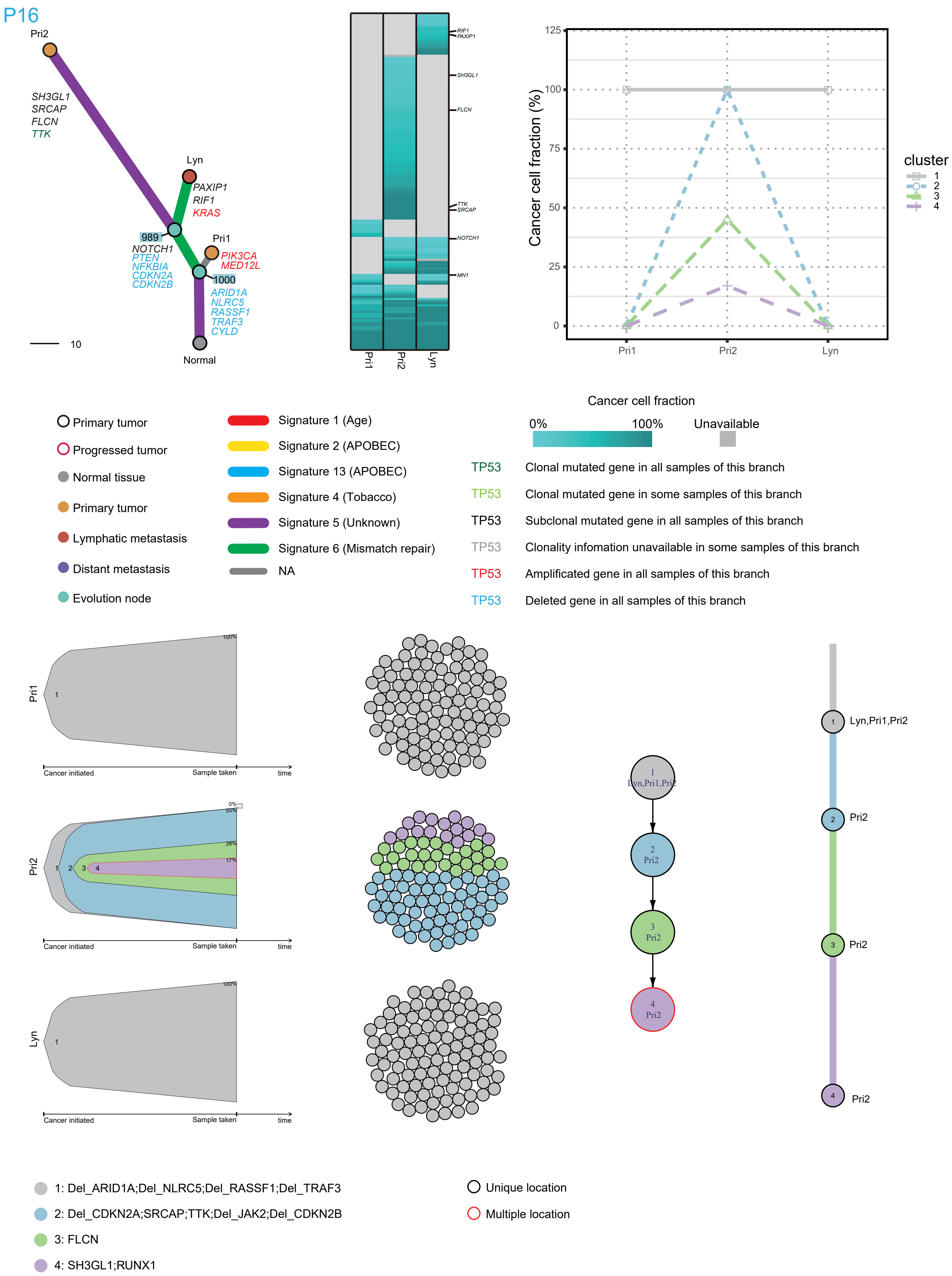
P16
Pri2
SH3GL1
SRCAP
FLCN
TTK
Lyn
PAXIP1
RIF1
KRAS
989
NOTCH1
PTEN
NFKBIA
CDKN2A
CDKN2B
Pri1
PIK3CA
MED12L
1000
ARID1A
NLRC5
RASSF1
TRAF3
CYLD
10
Normal
RIF1
PAXIP1
SH3GL1
FLCN
TTK
SRCAP
NOTCH1
MN1
Pri1
Pri2
Lyn
Cancer cell fraction (%)
125
100
75
50
25
0
Pri1
Pri2
Lyn
cluster
1
2
3
4
Primary tumor
Progressed tumor
Normal tissue
Primary tumor
Lymphatic metastasis
Distant metastasis
Evolution node
Signature 1 (Age)
Signature 2 (APOBEC)
Signature 13 (APOBEC)
Signature 4 (Tobacco)
Signature 5 (Unknown)
Signature 6 (Mismatch repair)
NA
Cancer cell fraction
0%
100%
Unavailable
TP53 Clonal mutated gene in all samples of this branch
TP53 Clonal mutated gene in some samples of this branch
TP53 Subclonal mutated gene in all samples of this branch
TP53 Clonality infomation unavailable in some samples of this branch
TP53 Amplificated gene in all samples of this branch
TP53 Deleted gene in all samples of this branch
Pri1
100%
1
Cancer initiated
Sample taken
time
Pri2
0%
55%
28%
17%
1
2
3
4
Cancer initiated
Sample taken
time
Lyn
100%
1
Cancer initiated
Sample taken
time
1
Lyn,Pri1,Pri2
2
Pri2
3
Pri2
4
Pri2
1 Lyn,Pri1,Pri2
2 Pri2
3 Pri2
4 Pri2
1: Del_ARID1A;Del_NLRC5;Del_RASSF1;Del_TRAF3
2: Del_CDKN2A;SRCAP;TTK;Del_JAK2;Del_CDKN2B
3: FLCN
4: SH3GL1;RUNX1
Unique location
Multiple location

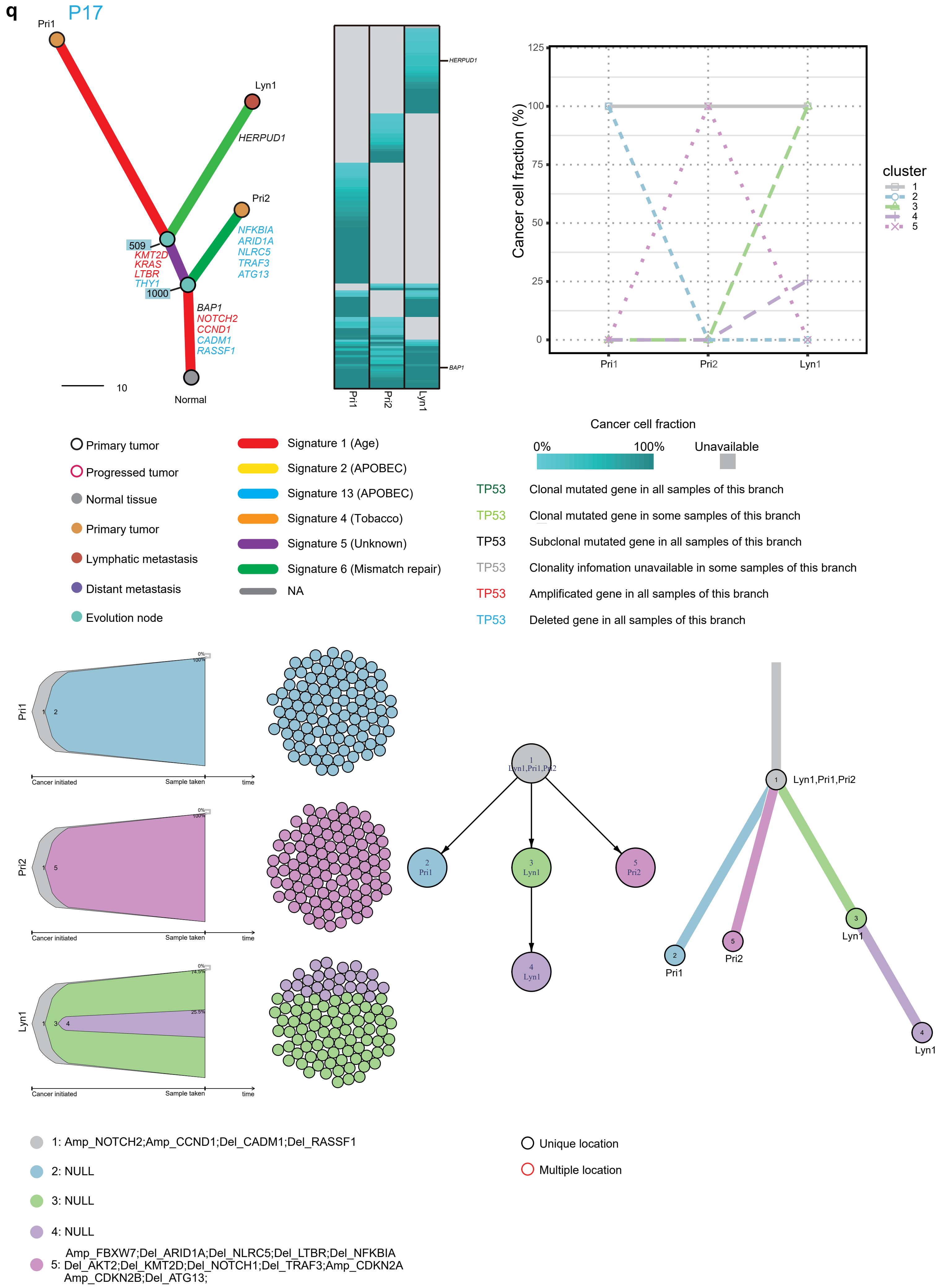
q
P17
Pri1
Lyn1
HERPUD1
Pri2
NFKBIA
ARID1A
NLRC5
TRAF3
ATG13
509
KMT2D
KRAS
LTBR
THY1
1000
BAP1
NOTCH2
CCND1
CADM1
RASSF1
10
Normal
HERPUD1
BAP1
Pri1
Pri2
Lyn1
Cancer cell fraction (%)
125
100
75
50
25
0
cluster
1
2
3
4
5
Cancer cell fraction
0%
100%
Unavailable
Primary tumor
Progressed tumor
Normal tissue
Primary tumor
Lymphatic metastasis
Distant metastasis
Evolution node
Signature 1 (Age)
Signature 2 (APOBEC)
Signature 13 (APOBEC)
Signature 4 (Tobacco)
Signature 5 (Unknown)
Signature 6 (Mismatch repair)
NA
TP53 Clonal mutated gene in all samples of this branch
TP53 Clonal mutated gene in some samples of this branch
TP53 Subclonal mutated gene in all samples of this branch
TP53 Clonality infomation unavailable in some samples of this branch
TP53 Amplificated gene in all samples of this branch
TP53 Deleted gene in all samples of this branch
Cancer initiated
Sample taken
time
1 Lyn1,Pri1,Pri2
2 Pri1
3 Lyn1
5 Pri2
4 Lyn1
Lyn1,Pri1,Pri2
1: Amp_NOTCH2;Amp_CCND1;Del_CADM1;Del_RASSF1
2: NULL
3: NULL
4: NULL
5: Amp_FBXW7;Del_ARID1A;Del_NLRC5;Del_LTBR;Del_NFKBIA Del_AKT2;Del_KMT2D;Del_NOTCH1;Del_TRAF3;Amp_CDKN2A Amp_CDKN2B;Del_ATG13;
Unique location
Multiple location

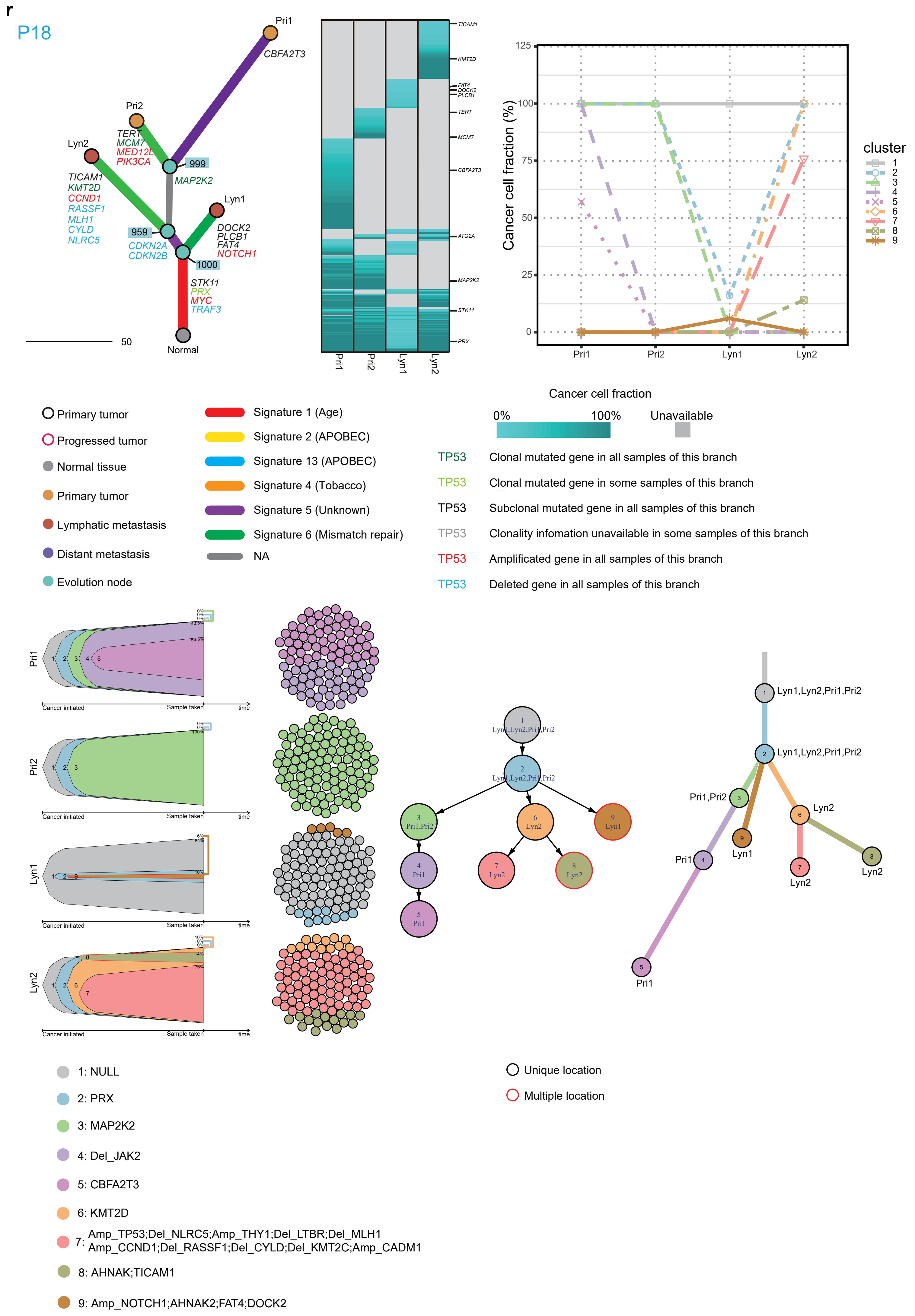
r
P18
Pri1
CBFA2T3
Pri2
TERT
MCM7
MED12L
PIK3CA
Lyn2
999
MAP2K2
TICAM1
KMT2D
CCND1
RASSF1
MLH1
CYLD
NLRC5
959
CDKN2A
CDKN2B
Lyn1
DOCK2
PLCB1
FAT4
NOTCH1
1000
STK11
PRX
MYC
TRAF3
50
Normal
TICAM1
KMT2D
FAT4
DOCK2
PLCB1
TERT
MCM7
CBFA2T3
ATG2A
MAP2K2
STK11
PRX
Pri1
Pri2
Lyn1
Lyn2
Cancer cell fraction (%)
125
100
75
50
25
0
cluster
1
2
3
4
5
6
7
8
9
Cancer cell fraction
0%
100%
Unavailable
Primary tumor
Progressed tumor
Normal tissue
Primary tumor
Lymphatic metastasis
Distant metastasis
Evolution node
Signature 1 (Age)
Signature 2 (APOBEC)
Signature 13 (APOBEC)
Signature 4 (Tobacco)
Signature 5 (Unknown)
Signature 6 (Mismatch repair)
NA
TP53 Clonal mutated gene in all samples of this branch
TP53 Clonal mutated gene in some samples of this branch
TP53 Subclonal mutated gene in all samples of this branch
TP53 Clonality infomation unavailable in some samples of this branch
TP53 Amplificated gene in all samples of this branch
TP53 Deleted gene in all samples of this branch
Cancer initiated
Sample taken
time
1 Lyn1,Lyn2,Pri1,Pri2
2 Lyn1,Lyn2,Pri1,Pri2
3 Pri1,Pri2
4 Pri1
5 Pri1
6 Lyn2
7 Lyn2
8 Lyn2
9 Lyn1
Unique location
Multiple location
1: NULL
2: PRX
3: MAP2K2
4: Del_JAK2
5: CBFA2T3
6: KMT2D
7: Amp_TP53;Del_NLRC5;Amp_THY1;Del_LTBR;Del_MLH1
Amp_CCND1;Del_RASSF1;Del_CYLD;Del_KMT2C;Amp_CADM1
8: AHNAK;TICAM1
9: Amp_NOTCH1;AHNAK2;FAT4;DOCK2

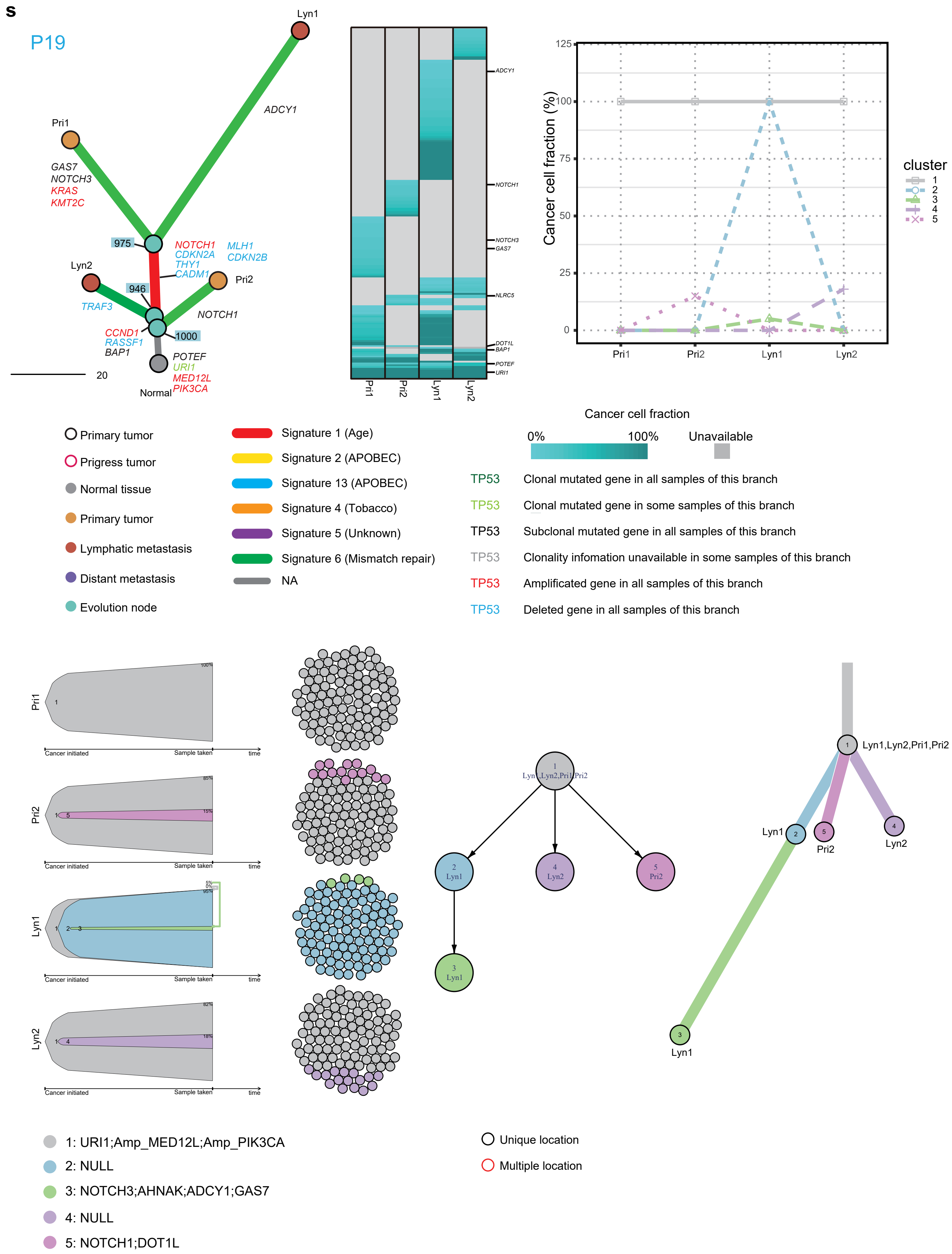
s
P19
Lyn1
ADCY1
Pri1
GAS7
NOTCH3
KRAS
KMT2C
975
NOTCH1
CDKN2A
THY1
CADM1
MLH1
CDKN2B
Lyn2
946
Pri2
TRAF3
NOTCH1
CCND1
RASSF1
BAP1
1000
POTEF
URI1
MED12L
PIK3CA
Normal
20
ADCY1
NOTCH1
NOTCH3
GAS7
NLRC5
DOT1L
BAP1
POTEF
URI1
Pri1
Pri2
Lyn1
Lyn2
Cancer cell fraction (%)
125
100
75
50
25
0
cluster
1
2
3
4
5
Cancer cell fraction
0%
100%
Unavailable
Primary tumor
Prigress tumor
Normal tissue
Primary tumor
Lymphatic metastasis
Distant metastasis
Evolution node
Signature 1 (Age)
Signature 2 (APOBEC)
Signature 13 (APOBEC)
Signature 4 (Tobacco)
Signature 5 (Unknown)
Signature 6 (Mismatch repair)
NA
TP53 Clonal mutated gene in all samples of this branch
TP53 Clonal mutated gene in some samples of this branch
TP53 Subclonal mutated gene in all samples of this branch
TP53 Clonality infomation unavailable in some samples of this branch
TP53 Amplificated gene in all samples of this branch
TP53 Deleted gene in all samples of this branch
Pri1
Cancer initiated
Sample taken
time
100%
Pri2
85%
15%
Lyn1
Lyn2
82%
18%
1 Lyn1,Lyn2,Pri1,Pri2
2 Lyn1
4 Lyn2
5 Pri2
3 Lyn1
Lyn1,Lyn2,Pri1,Pri2
Lyn1
Pri2
Lyn2
Lyn1
1: URI1;Amp_MED12L;Amp_PIK3CA
2: NULL
3: NOTCH3;AHNAK;ADCY1;GAS7
4: NULL
5: NOTCH1;DOT1L
Unique location
Multiple location

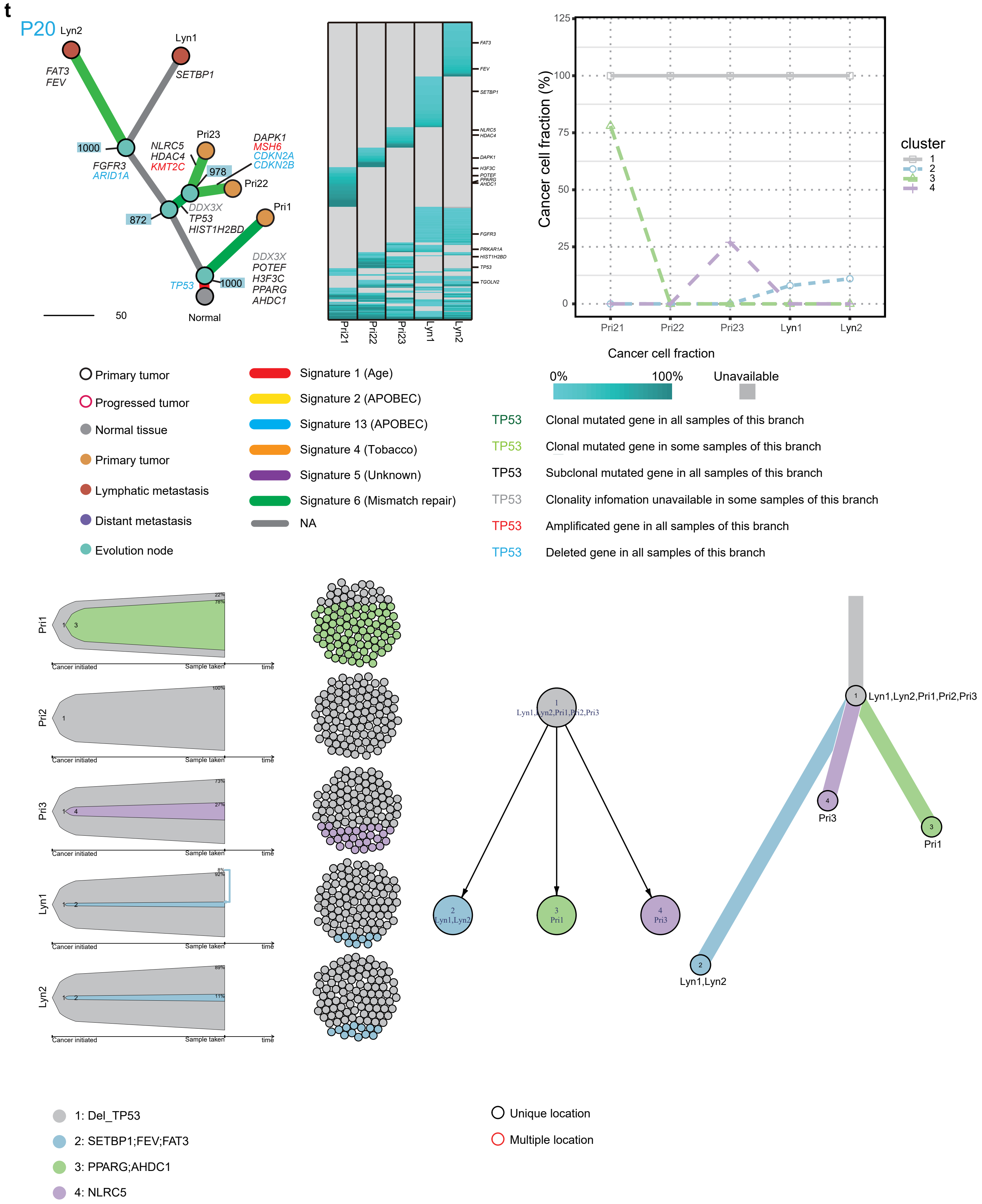
t
P20
Lyn2
Lyn1
FAT3
FEV
SETBP1
1000
NLRC5
HDAC4
KMT2C
Pri23
DAPK1
MSH6
CDKN2A
CDKN2B
FGFR3
ARID1A
978
Pri22
872
DDX3X
TP53
HIST1H2BD
Pri1
DDX3X
POTEF
H3F3C
PPARG
AHDC1
TP53
1000
50
Normal
FAT3
FEV
SETBP1
NLRC5
HDAC4
DAPK1
H3F3C
POTEF
PPARG
AHDC1
FGFR3
PRKAR1A
HIST1H2BD
TP53
TGOLN2
Pri21
Pri22
Pri23
Lyn1
Lyn2
Cancer cell fraction (%)
125
100
75
50
25
0
Pri21
Pri22
Pri23
Lyn1
Lyn2
Cancer cell fraction
cluster
1
2
3
4
Primary tumor
Progressed tumor
Normal tissue
Primary tumor
Lymphatic metastasis
Distant metastasis
Evolution node
Signature 1 (Age)
Signature 2 (APOBEC)
Signature 13 (APOBEC)
Signature 4 (Tobacco)
Signature 5 (Unknown)
Signature 6 (Mismatch repair)
NA
0%
100%
Unavailable
TP53 Clonal mutated gene in all samples of this branch
TP53 Clonal mutated gene in some samples of this branch
TP53 Subclonal mutated gene in all samples of this branch
TP53 Clonality infomation unavailable in some samples of this branch
TP53 Amplificated gene in all samples of this branch
TP53 Deleted gene in all samples of this branch
Pri1
22%
78%
Cancer initiated
Sample taken
time
Pri2
100%
Pri3
73%
27%
Lyn1
8%
92%
Lyn2
89%
11%
1
Lyn1,Lyn2,Pri1,Pri2,Pri3
2
Lyn1,Lyn2
3
Pri1
4
Pri3
Lyn1,Lyn2,Pri1,Pri2,Pri3
Pri3
Pri1
Lyn1,Lyn2
1: Del_TP53
2: SETBP1;FEV;FAT3
3: PPARG;AHDC1
4: NLRC5
Unique location
Multiple location

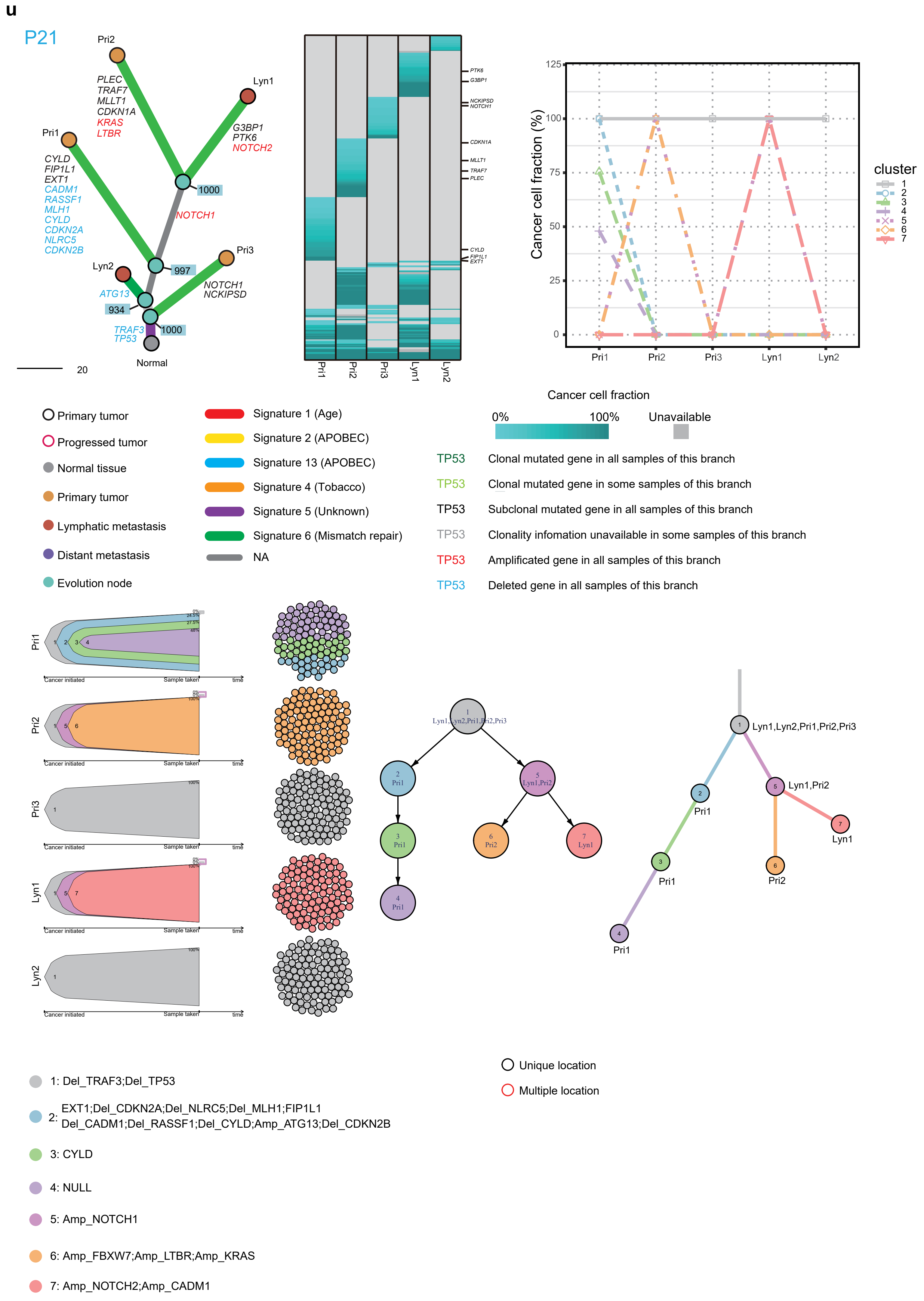
u
P21
Pri2
Lyn1
Pri1
Lyn2
Pri3
Normal
PLEC
TRAF7
MLLT1
CDKN1A
KRAS
LTBR
G3BP1
PTK6
NOTCH2
CYLD
FIP1L1
EXT1
CADM1
RASSF1
MLH1
CYLD
CDKN2A
NLRC5
CDKN2B
NOTCH1
ATG13
NOTCH1
NCKIPSD
TRAF3
TP53
1000
997
934
1000
20
PTK6
G3BP1
NCKIPSD
NOTCH1
CDKN1A
MLLT1
TRAF7
PLEC
CYLD
FIP1L1
EXT1
Pri1
Pri2
Pri3
Lyn1
Lyn2
Cancer cell fraction (%)
125
100
75
50
25
0
cluster
1
2
3
4
5
6
7
Cancer cell fraction
0%
100%
Unavailable
Primary tumor
Progressed tumor
Normal tissue
Primary tumor
Lymphatic metastasis
Distant metastasis
Evolution node
Signature 1 (Age)
Signature 2 (APOBEC)
Signature 13 (APOBEC)
Signature 4 (Tobacco)
Signature 5 (Unknown)
Signature 6 (Mismatch repair)
NA
TP53 Clonal mutated gene in all samples of this branch
TP53 Clonal mutated gene in some samples of this branch
TP53 Subclonal mutated gene in all samples of this branch
TP53 Clonality infomation unavailable in some samples of this branch
TP53 Amplificated gene in all samples of this branch
TP53 Deleted gene in all samples of this branch
Cancer initiated
Sample taken
time
1
Lyn1,Lyn2,Pri1,Pri2,Pri3
2
Pri1
3
Pri1
4
Pri1
5
Lyn1,Pri2
6
Pri2
7
Lyn1
Unique location
Multiple location
1: Del_TRAF3;Del_TP53
2: EXT1;Del_CDKN2A;Del_NLRC5;Del_MLH1;FIP1L1
Del_CADM1;Del_RASSF1;Del_CYLD;Amp_ATG13;Del_CDKN2B
3: CYLD
4: NULL
5: Amp_NOTCH1
6: Amp_FBXW7;Amp_LTBR;Amp_KRAS
7: Amp_NOTCH2;Amp_CADM1

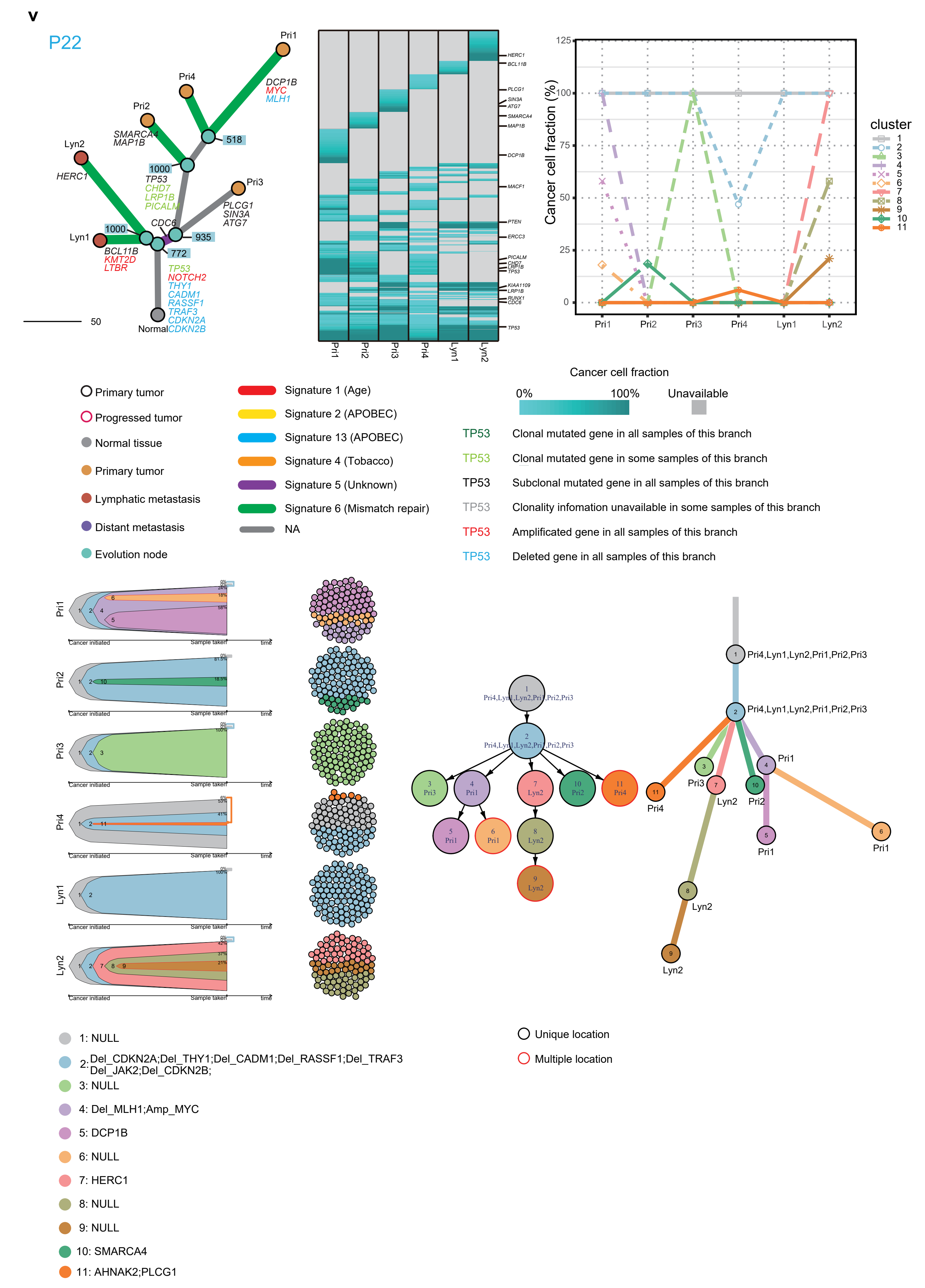
v
P22
Pri1
Pri4
Pri2
Lyn2
Pri3
Lyn1
Normal
DCP1B
MYC
MLH1
518
SMARCA4
MAP1B
1000
TP53
CHD7
LRP1B
PICALM
HERC1
PLCG1
SIN3A
ATG7
CDC6
1000
935
BCL11B
KMT2D
LTBR
772
TP53
NOTCH2
THY1
CADM1
RASSF1
TRAF3
CDKN2A
CDKN2B
50
HERC1
BCL11B
PLCG1
SIN3A
ATG7
SMARCA4
MAP1B
DCP1B
MACF1
PTEN
ERCC3
PICALM
CHD7
LRP1B
TP53
KIAA1109
LRP1B
RUNX1
CDC6
TP53
Pri1
Pri2
Pri3
Pri4
Lyn1
Lyn2
Cancer cell fraction (%)
125
100
75
50
25
0
Pri1
Pri2
Pri3
Pri4
Lyn1
Lyn2
cluster
1
2
3
4
5
6
7
8
9
10
11
Cancer cell fraction
0%
100%
Unavailable
Primary tumor
Progressed tumor
Normal tissue
Primary tumor
Lymphatic metastasis
Distant metastasis
Evolution node
Signature 1 (Age)
Signature 2 (APOBEC)
Signature 13 (APOBEC)
Signature 4 (Tobacco)
Signature 5 (Unknown)
Signature 6 (Mismatch repair)
NA
TP53 Clonal mutated gene in all samples of this branch
TP53 Clonal mutated gene in some samples of this branch
TP53 Subclonal mutated gene in all samples of this branch
TP53 Clonality infomation unavailable in some samples of this branch
TP53 Amplificated gene in all samples of this branch
TP53 Deleted gene in all samples of this branch
Pri1
Pri2
Pri3
Pri4
Lyn1
Lyn2
Cancer initiated
Sample taken
time
Pri4,Lyn1,Lyn2,Pri1,Pri2,Pri3
Pri4,Lyn1,Lyn2,Pri1,Pri2,Pri3
1: NULL
2: Del_CDKN2A;Del_THY1;Del_CADM1;Del_RASSF1;Del_TRAF3
Del_JAK2;Del_CDKN2B;
3: NULL
4: Del_MLH1;Amp_MYC
5: DCP1B
6: NULL
7: HERC1
8: NULL
9: NULL
10: SMARCA4
11: AHNAK2;PLCG1
Unique location
Multiple location

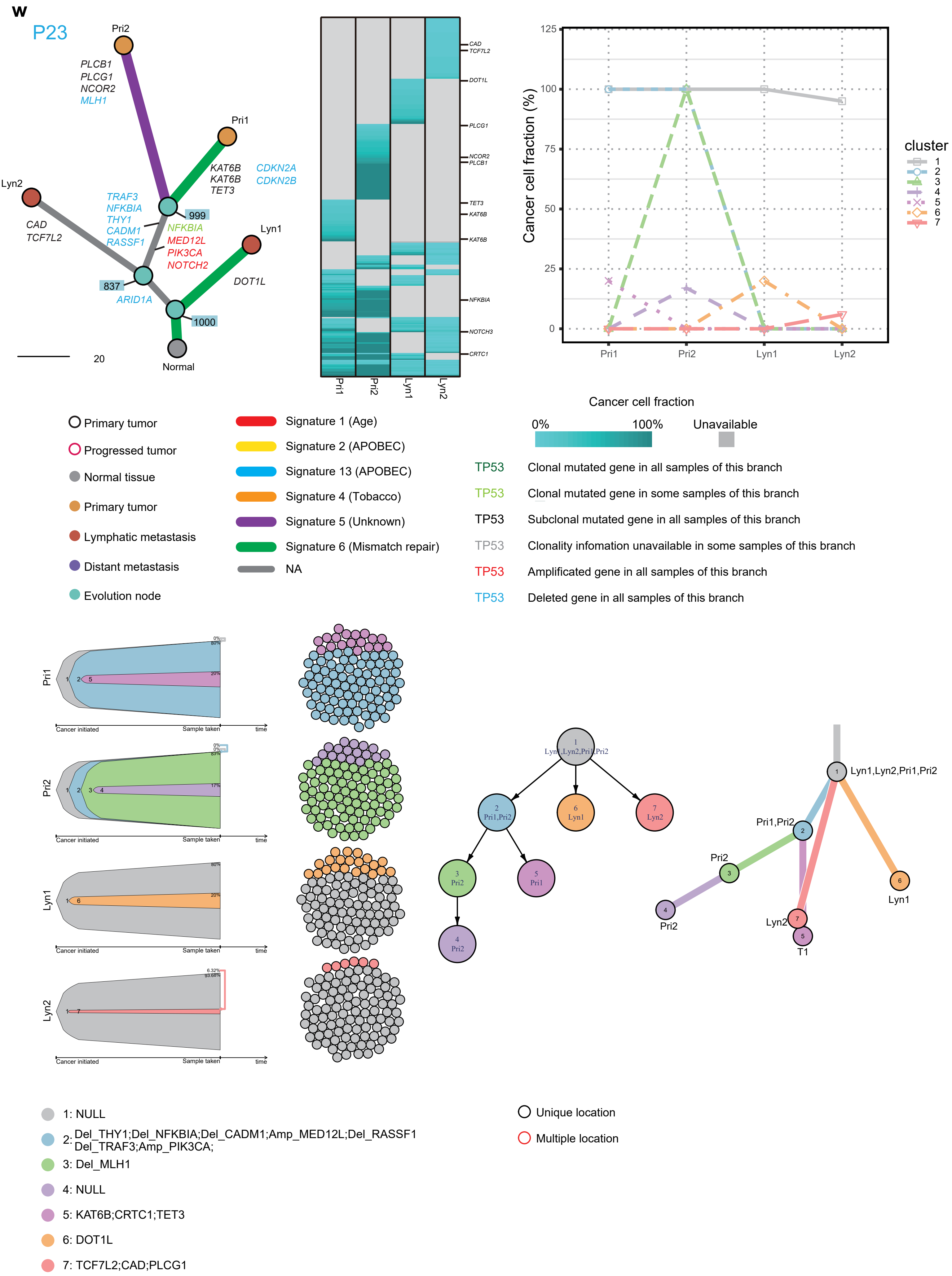
w
P23
Pri2
PLCB1
PLCG1
NCOR2
MLH1
Pri1
KAT6B
KAT6B
TET3
CDKN2A
CDKN2B
Lyn2
CAD
TCF7L2
TRAF3
NFKBIA
THY1
CADM1
RASSF1
999
NFKBIA
MED12L
PIK3CA
NOTCH2
Lyn1
DOT1L
837
ARID1A
1000
Normal
20
Cancer cell fraction (%)
cluster
Pri1
Pri2
Lyn1
Lyn2
Cancer cell fraction
0%
100%
Unavailable
Primary tumor
Progressed tumor
Normal tissue
Primary tumor
Lymphatic metastasis
Distant metastasis
Evolution node
Signature 1 (Age)
Signature 2 (APOBEC)
Signature 13 (APOBEC)
Signature 4 (Tobacco)
Signature 5 (Unknown)
Signature 6 (Mismatch repair)
NA
TP53 Clonal mutated gene in all samples of this branch
TP53 Clonal mutated gene in some samples of this branch
TP53 Subclonal mutated gene in all samples of this branch
TP53 Clonality infomation unavailable in some samples of this branch
TP53 Amplificated gene in all samples of this branch
TP53 Deleted gene in all samples of this branch
Cancer initiated
Sample taken
time
Lyn1,Lyn2,Pri1,Pri2
Pri1,Pri2
Lyn1
Lyn2
Pri2
Pri1
T1
1: NULL
2: Del_THY1;Del_NFKBIA;Del_CADM1;Amp_MED12L;Del_RASSF1
Del_TRAF3;Amp_PIK3CA;
3: Del_MLH1
4: NULL
5: KAT6B;CRTC1;TET3
6: DOT1L
7: TCF7L2;CAD;PLCG1
Unique location
Multiple location

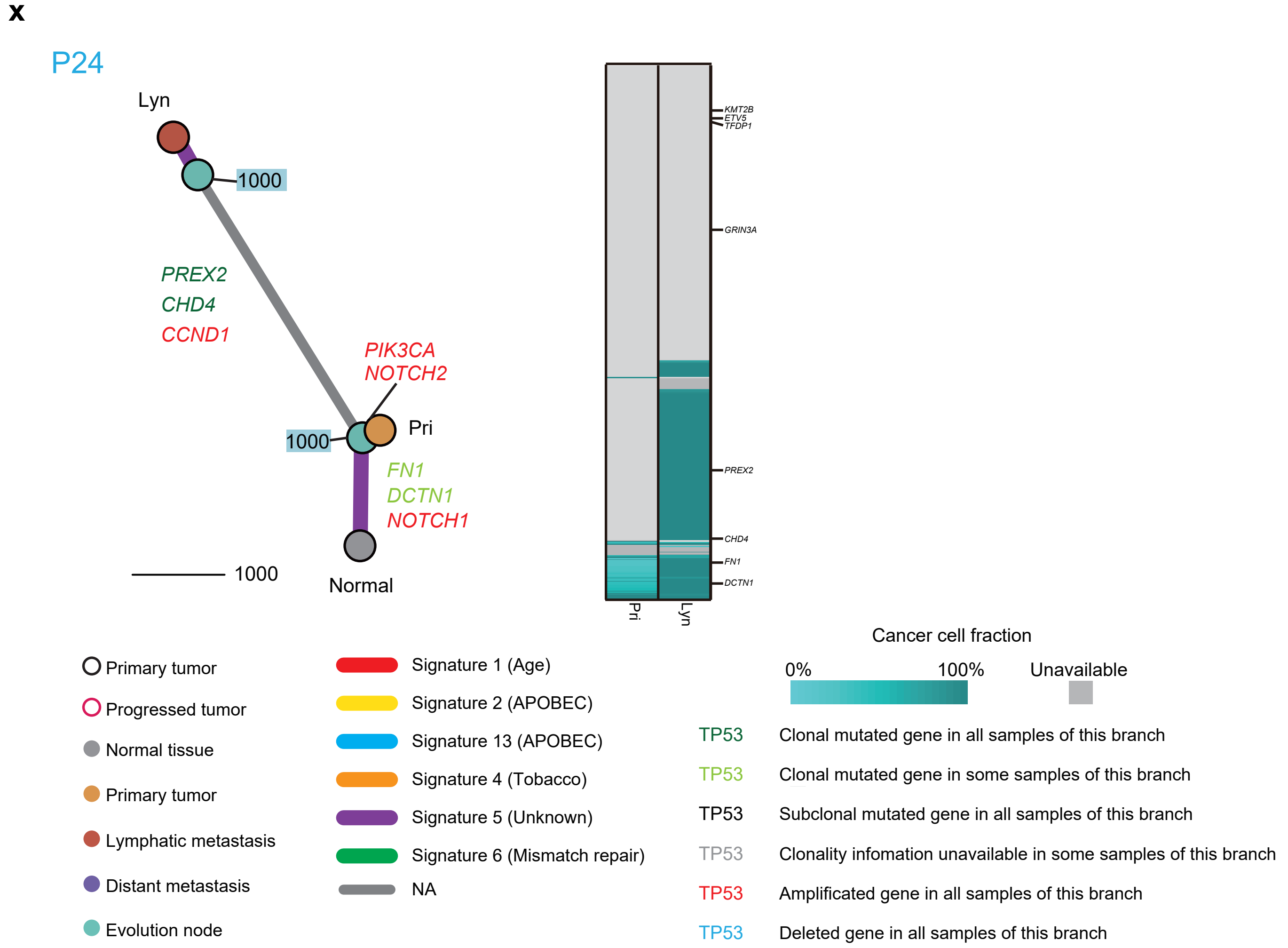
x
P24
Lyn
1000
PREX2
CHD4
CCND1
PIK3CA
NOTCH2
Pri
1000
FN1
DCTN1
NOTCH1
1000
Normal
KMT2B
ETV5
TFDP1
GRIN3A
PREX2
CHD4
FN1
DCTN1
Pri
Lyn
Cancer cell fraction
0%
100%
Unavailable
Primary tumor
Progressed tumor
Normal tissue
Primary tumor
Lymphatic metastasis
Distant metastasis
Evolution node
Signature 1 (Age)
Signature 2 (APOBEC)
Signature 13 (APOBEC)
Signature 4 (Tobacco)
Signature 5 (Unknown)
Signature 6 (Mismatch repair)
NA
TP53 Clonal mutated gene in all samples of this branch
TP53 Clonal mutated gene in some samples of this branch
TP53 Subclonal mutated gene in all samples of this branch
TP53 Clonality infomation unavailable in some samples of this branch
TP53 Amplificated gene in all samples of this branch
TP53 Deleted gene in all samples of this branch

y

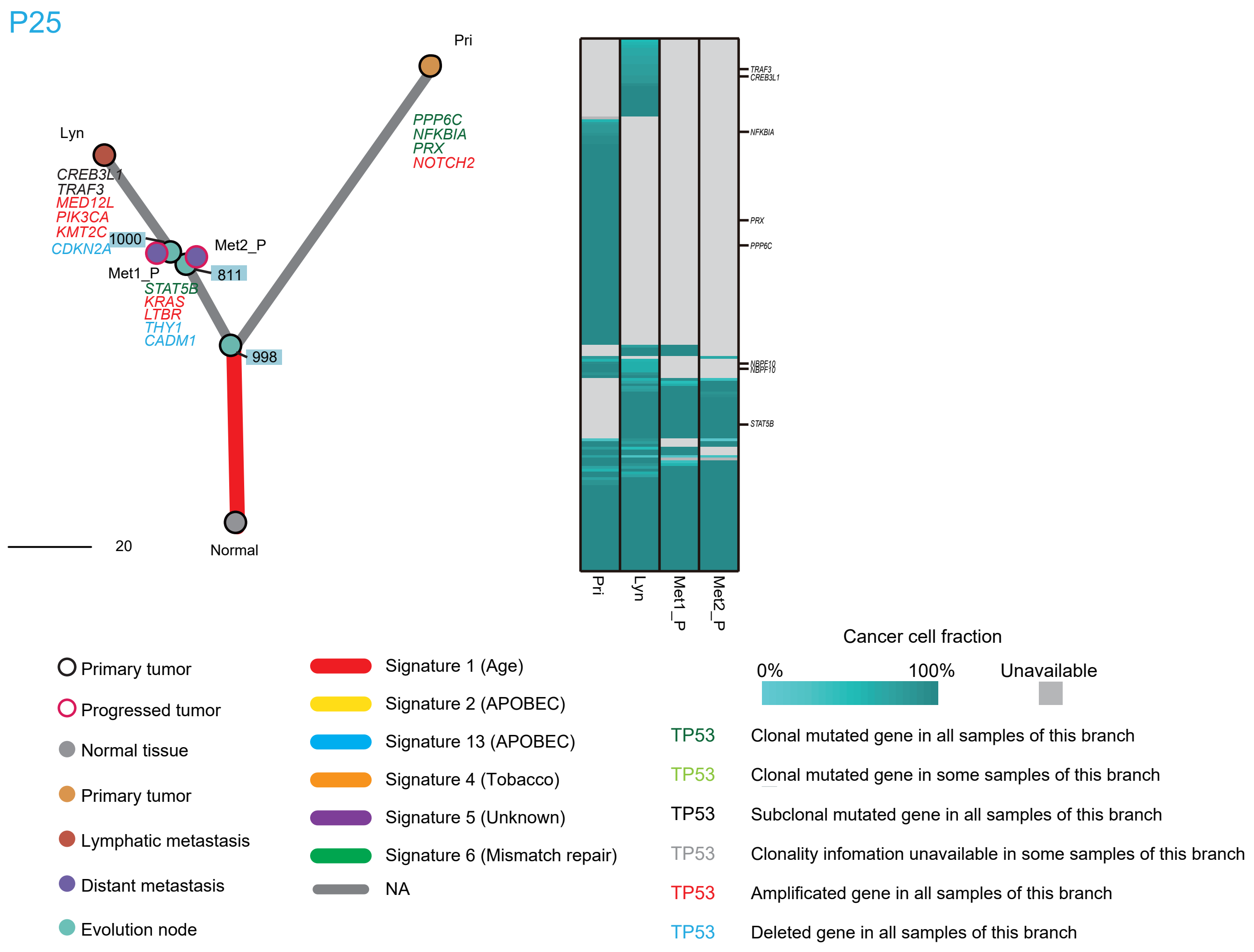
P25
Pri
Lyn
PPP6C
NFKBIA
PRX
NOTCH2
CREB3L1
TRAF3
MED12L
PIK3CA
KMT2C
CDKN2A
1000
Met2_P
Met1_P
811
STAT5B
KRAS
LTBR
THY1
CADM1
998
Normal
20
TRAF3
CREB3L1
NFKBIA
PRX
PPP6C
NBPF10
NBPF10
STAT5B
Pri
Lyn
Met1_P
Met2_P
Cancer cell fraction
0%
100%
Unavailable
Primary tumor
Progressed tumor
Normal tissue
Primary tumor
Lymphatic metastasis
Distant metastasis
Evolution node
Signature 1 (Age)
Signature 2 (APOBEC)
Signature 13 (APOBEC)
Signature 4 (Tobacco)
Signature 5 (Unknown)
Signature 6 (Mismatch repair)
NA
TP53 Clonal mutated gene in all samples of this branch
TP53 Clonal mutated gene in some samples of this branch
TP53 Subclonal mutated gene in all samples of this branch
TP53 Clonality infomation unavailable in some samples of this branch
TP53 Amplificated gene in all samples of this branch
TP53 Deleted gene in all samples of this branch

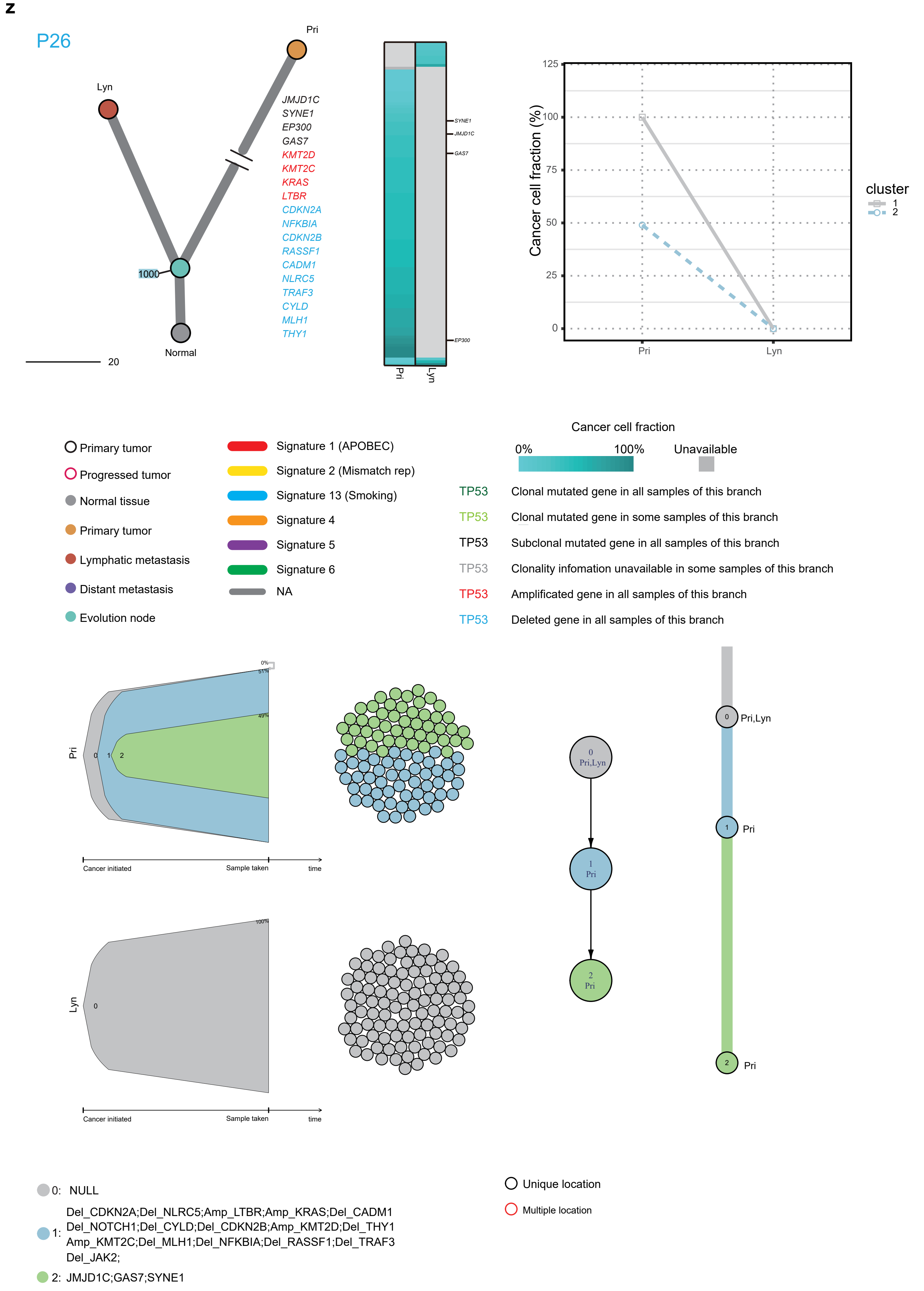
z
P26
Pri
Lyn
JMJD1C
SYNE1
EP300
GAS7
KMT2D
KMT2C
KRAS
LTBR
CDKN2A
NFKBIA
CDKN2B
RASSF1
CADM1
NLRC5
TRAF3
CYLD
MLH1
THY1
1000
Normal
20
SYNE1
JMJD1C
GAS7
EP300
Pri
Lyn
125
100
75
50
25
0
Cancer cell fraction (%)
Pri
Lyn
cluster
1
2
Primary tumor
Progressed tumor
Normal tissue
Primary tumor
Lymphatic metastasis
Distant metastasis
Evolution node
Signature 1 (APOBEC)
Signature 2 (Mismatch rep)
Signature 13 (Smoking)
Signature 4
Signature 5
Signature 6
NA
Cancer cell fraction
0%
100%
Unavailable
TP53 Clonal mutated gene in all samples of this branch
TP53 Clonal mutated gene in some samples of this branch
TP53 Subclonal mutated gene in all samples of this branch
TP53 Clonality infomation unavailable in some samples of this branch
TP53 Amplificated gene in all samples of this branch
TP53 Deleted gene in all samples of this branch
Pri
0 1 2
0%
51%
49%
Cancer initiated
Sample taken
time
Lyn
0
100%
Cancer initiated
Sample taken
time
0
Pri,Lyn
1
Pri
2
Pri
0 Pri,Lyn
1 Pri
2 Pri
0: NULL
1: Del_CDKN2A;Del_NLRC5;Amp_LTBR;Amp_KRAS;Del_CADM1
Del_NOTCH1;Del_CYLD;Del_CDKN2B;Amp_KMT2D;Del_THY1
Amp_KMT2C;Del_MLH1;Del_NFKBIA;Del_RASSF1;Del_TRAF3
Del_JAK2;
2: JMJD1C;GAS7;SYNE1
Unique location
Multiple location

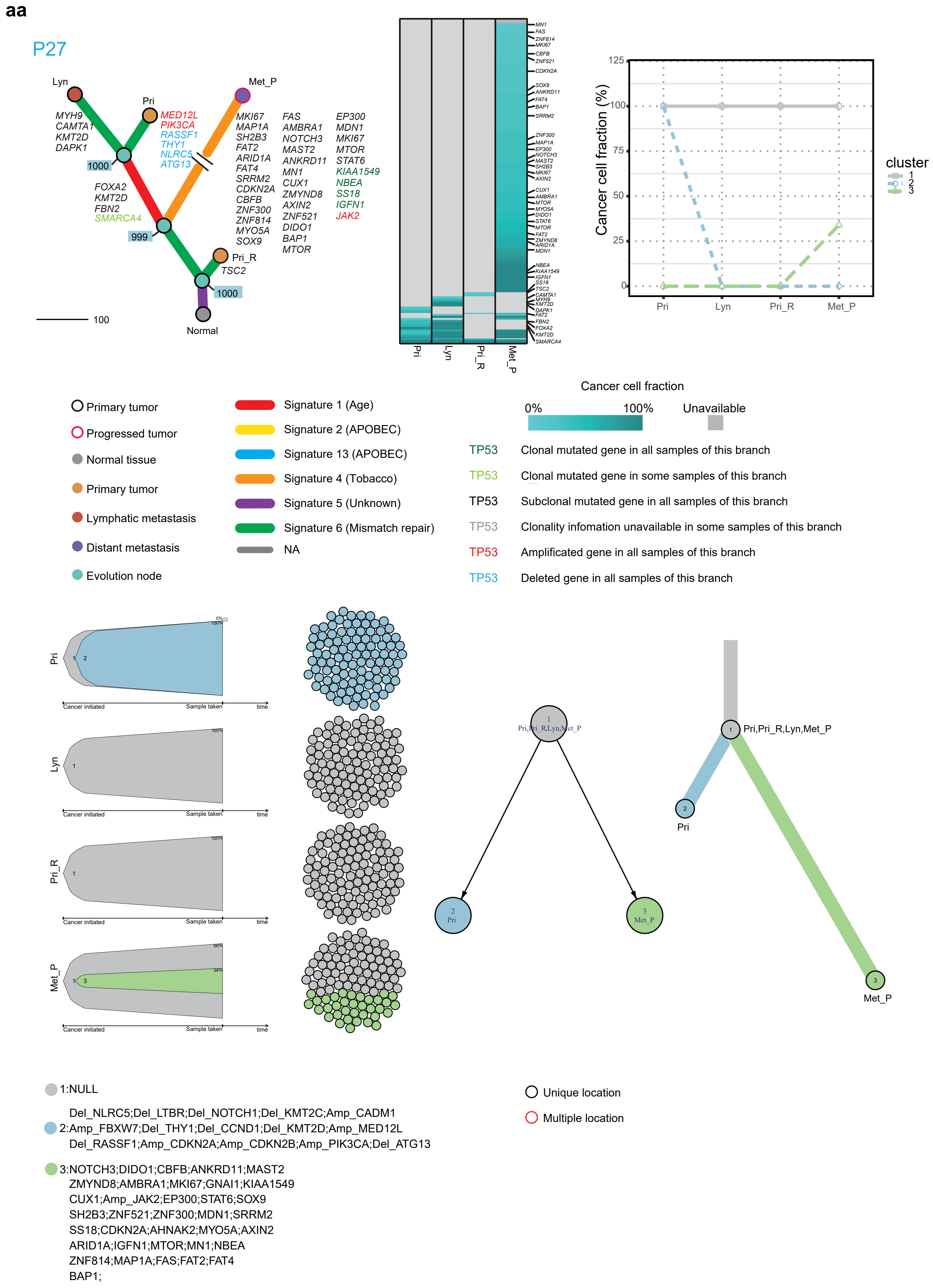

aa
P27
Lyn
Pri
Met_P
MYH9
CAMTA1
KMT2D
DAPK1
MED12L
PIK3CA
RASSF1
THY1
NLRC5
ATG13
1000
FOXA2
KMT2D
FBN2
SMARCA4
999
Pri_R
TSC2
1000
Normal
100
MKI67 MAP1A SH2B3 FAT2 ARID1A FAT4 SRRM2 CDKN2A CBFB ZNF300 ZNF814 MYO5A SOX9
FAS AMBRA1 NOTCH3 MAST2 ANKRD11 MN1 CUX1 ZMYND8 AXIN2 ZNF521 DIDO1 BAP1 MTOR
EP300 MDN1 MKI67 MTOR STAT6 KIAA1549 NBEA SS18 IGFN1 JAK2
Pri
Lyn
Pri_R
Met_P
Cancer cell fraction (%)
cluster
1
2
3
Cancer cell fraction
0%
100%
Unavailable
Primary tumor
Progressed tumor
Normal tissue
Primary tumor
Lymphatic metastasis
Distant metastasis
Evolution node
Signature 1 (Age)
Signature 2 (APOBEC)
Signature 13 (APOBEC)
Signature 4 (Tobacco)
Signature 5 (Unknown)
Signature 6 (Mismatch repair)
NA
TP53 Clonal mutated gene in all samples of this branch
TP53 Clonal mutated gene in some samples of this branch
TP53 Subclonal mutated gene in all samples of this branch
TP53 Clonality infomation unavailable in some samples of this branch
TP53 Amplificated gene in all samples of this branch
TP53 Deleted gene in all samples of this branch
Cancer initiated
Sample taken
time
Pri,Pri_R,Lyn,Met_P
1:NULL
2:Amp_FBXW7;Del_THY1;Del_CCND1;Del_KMT2D;Amp_MED12L
Del_NLRC5;Del_LTBR;Del_NOTCH1;Del_KMT2C;Amp_CADM1
Del_RASSF1;Amp_CDKN2A;Amp_CDKN2B;Amp_PIK3CA;Del_ATG13
3:NOTCH3;DIDO1;CBFB;ANKRD11;MAST2
ZMYND8;AMBRA1;MKI67;GNAI1;KIAA1549
CUX1;Amp_JAK2;EP300;STAT6;SOX9
SH2B3;ZNF521;ZNF300;MDN1;SRRM2
SS18;CDKN2A;AHNAK2;MYO5A;AXIN2
ARID1A;IGFN1;MTOR;MN1;NBEA
ZNF814;MAP1A;FAS;FAT2;FAT4
BAP1;
Unique location
Multiple location

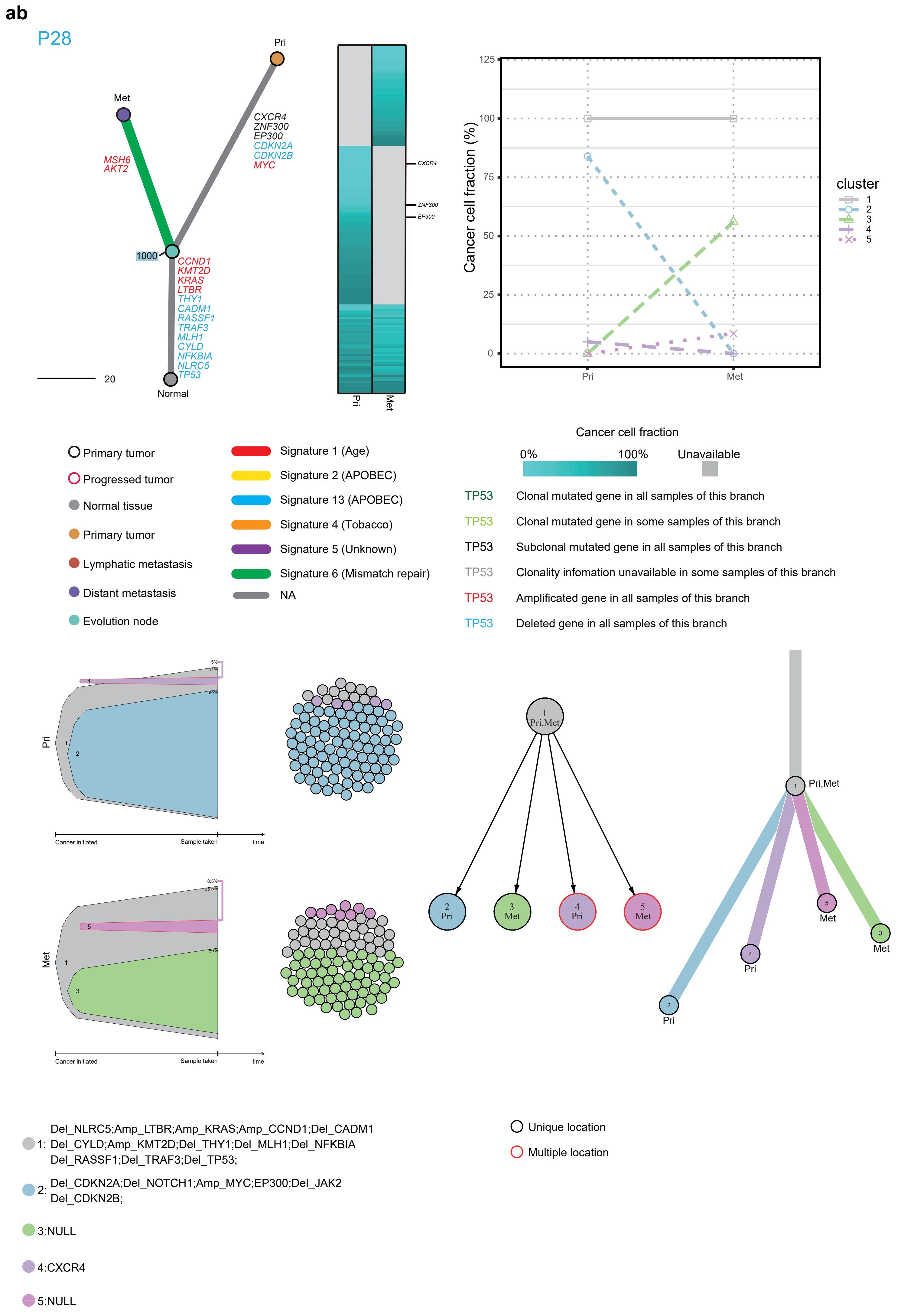
ab
P28
Pri
Met
MSH6
AKT2
CXCR4
ZNF300
EP300
CDKN2A
CDKN2B
MYC
1000
CCND1
KMT2D
KRAS
LTBR
THY1
CADM1
RASSF1
TRAF3
MLH1
CYLD
NFKBIA
NLRC5
TP53
20
Normal
CXCR4
ZNF300
EP300
Pri
Met
Cancer cell fraction (%)
125
100
75
50
25
0
Pri
Met
cluster
1
2
3
4
5
Primary tumor
Progressed tumor
Normal tissue
Primary tumor
Lymphatic metastasis
Distant metastasis
Evolution node
Signature 1 (Age)
Signature 2 (APOBEC)
Signature 13 (APOBEC)
Signature 4 (Tobacco)
Signature 5 (Unknown)
Signature 6 (Mismatch repair)
NA
Cancer cell fraction
0%
100%
Unavailable
TP53 Clonal mutated gene in all samples of this branch
TP53 Clonal mutated gene in some samples of this branch
TP53 Subclonal mutated gene in all samples of this branch
TP53 Clonality infomation unavailable in some samples of this branch
TP53 Amplificated gene in all samples of this branch
TP53 Deleted gene in all samples of this branch
Pri
5%
11%
84%
4
1
2
Cancer initiated
Sample taken
time
Met
8.5%
35.5%
56%
5
1
3
Cancer initiated
Sample taken
time
1 Pri,Met
2 Pri
3 Met
4 Pri
5 Met
1 Pri,Met
5 Met
3 Met
4 Pri
2 Pri
1: Del_NLRC5;Amp_LTBR;Amp_KRAS;Amp_CCND1;Del_CADM1 Del_CYLD;Amp_KMT2D;Del_THY1;Del_MLH1;Del_NFKBIA Del_RASSF1;Del_TRAF3;Del_TP53;
2: Del_CDKN2A;Del_NOTCH1;Amp_MYC;EP300;Del_JAK2 Del_CDKN2B;
3:NULL
4:CXCR4
5:NULL
Unique location
Multiple location

ac

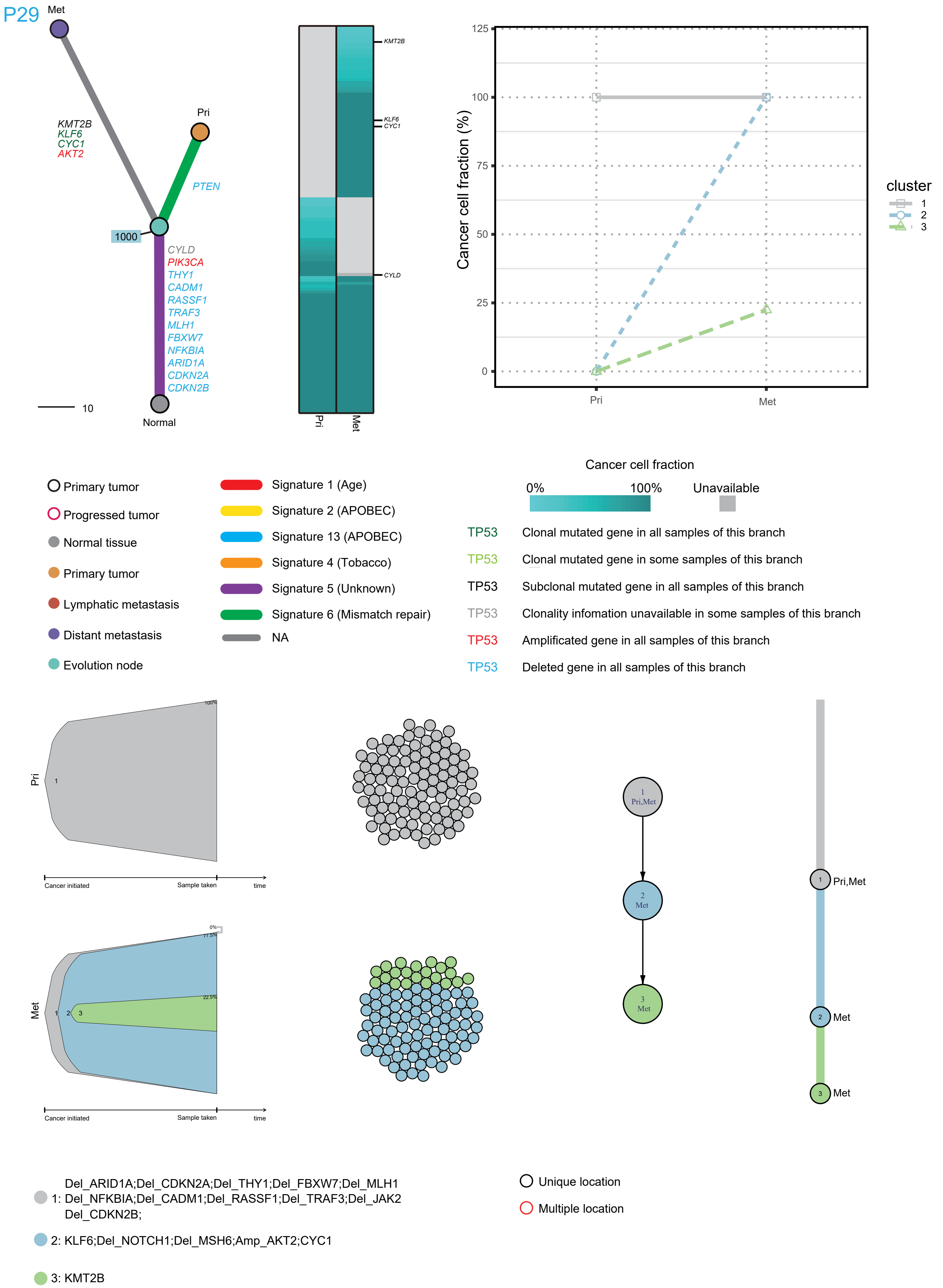

P29
Met
Pri
Normal
1000
10
KMT2B
KLF6
CYC1
AKT2
PTEN
CYLD
PIK3CA
THY1
CADM1
RASSF1
TRAF3
MLH1
FBXW7
NFKBIA
ARID1A
CDKN2A
CDKN2B
Pri
Met
Cancer cell fraction (%)
125
100
75
50
25
0
cluster
1
2
3
Primary tumor
Progressed tumor
Normal tissue
Primary tumor
Lymphatic metastasis
Distant metastasis
Evolution node
Signature 1 (Age)
Signature 2 (APOBEC)
Signature 13 (APOBEC)
Signature 4 (Tobacco)
Signature 5 (Unknown)
Signature 6 (Mismatch repair)
NA
Cancer cell fraction
0%
100%
Unavailable
TP53 Clonal mutated gene in all samples of this branch
TP53 Clonal mutated gene in some samples of this branch
TP53 Subclonal mutated gene in all samples of this branch
TP53 Clonality infomation unavailable in some samples of this branch
TP53 Amplificated gene in all samples of this branch
TP53 Deleted gene in all samples of this branch
Pri
1
100%
Cancer initiated
Sample taken
time
Met
1
2
3
0%
77.5%
22.5%
Cancer initiated
Sample taken
time
1 Pri,Met
2 Met
3 Met
1 Pri,Met
2 Met
3 Met
1: Del_ARID1A;Del_CDKN2A;Del_THY1;Del_FBXW7;Del_MLH1
Del_NFKBIA;Del_CADM1;Del_RASSF1;Del_TRAF3;Del_JAK2
Del_CDKN2B;
2: KLF6;Del_NOTCH1;Del_MSH6;Amp_AKT2;CYC1
3: KMT2B
Unique location
Multiple location

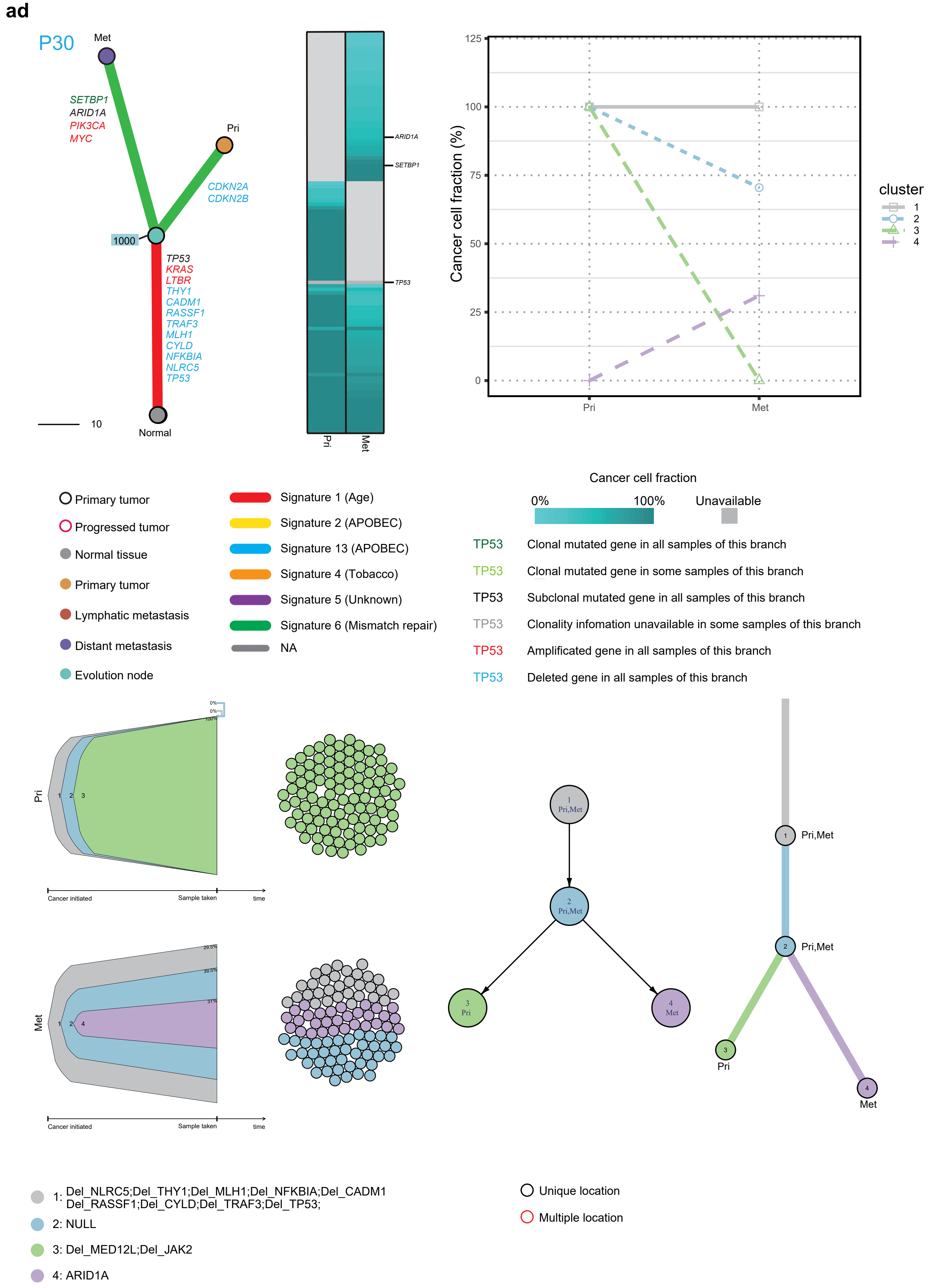
ad
P30
Met
SETBP1
ARID1A
PIK3CA
MYC
Pri
CDKN2A
CDKN2B
1000
TP53
KRAS
LTBR
THY1
CADM1
RASSF1
TRAF3
MLH1
CYLD
NFKBIA
NLRC5
TP53
10
Normal
ARID1A
SETBP1
TP53
Pri
Met
Cancer cell fraction (%)
125
100
75
50
25
0
Pri
Met
cluster
1
2
3
4
Primary tumor
Progressed tumor
Normal tissue
Primary tumor
Lymphatic metastasis
Distant metastasis
Evolution node
Signature 1 (Age)
Signature 2 (APOBEC)
Signature 13 (APOBEC)
Signature 4 (Tobacco)
Signature 5 (Unknown)
Signature 6 (Mismatch repair)
NA
Cancer cell fraction
0%
100%
Unavailable
TP53 Clonal mutated gene in all samples of this branch
TP53 Clonal mutated gene in some samples of this branch
TP53 Subclonal mutated gene in all samples of this branch
TP53 Clonality infomation unavailable in some samples of this branch
TP53 Amplificated gene in all samples of this branch
TP53 Deleted gene in all samples of this branch
0%
0%
100%
Pri
1 2 3
Cancer initiated
Sample taken
time
29.5%
39.5%
31%
Met
1 2 4
Cancer initiated
Sample taken
time
1 Pri,Met
2 Pri,Met
3 Pri
4 Met
1 Pri,Met
2 Pri,Met
3 Pri
4 Met
1: Del_NLRC5;Del_THY1;Del_MLH1;Del_NFKBIA;Del_CADM1 Del_RASSF1;Del_CYLD;Del_TRAF3;Del_TP53;
2: NULL
3: Del_MED12L;Del_JAK2
4: ARID1A
Unique location
Multiple location

ae

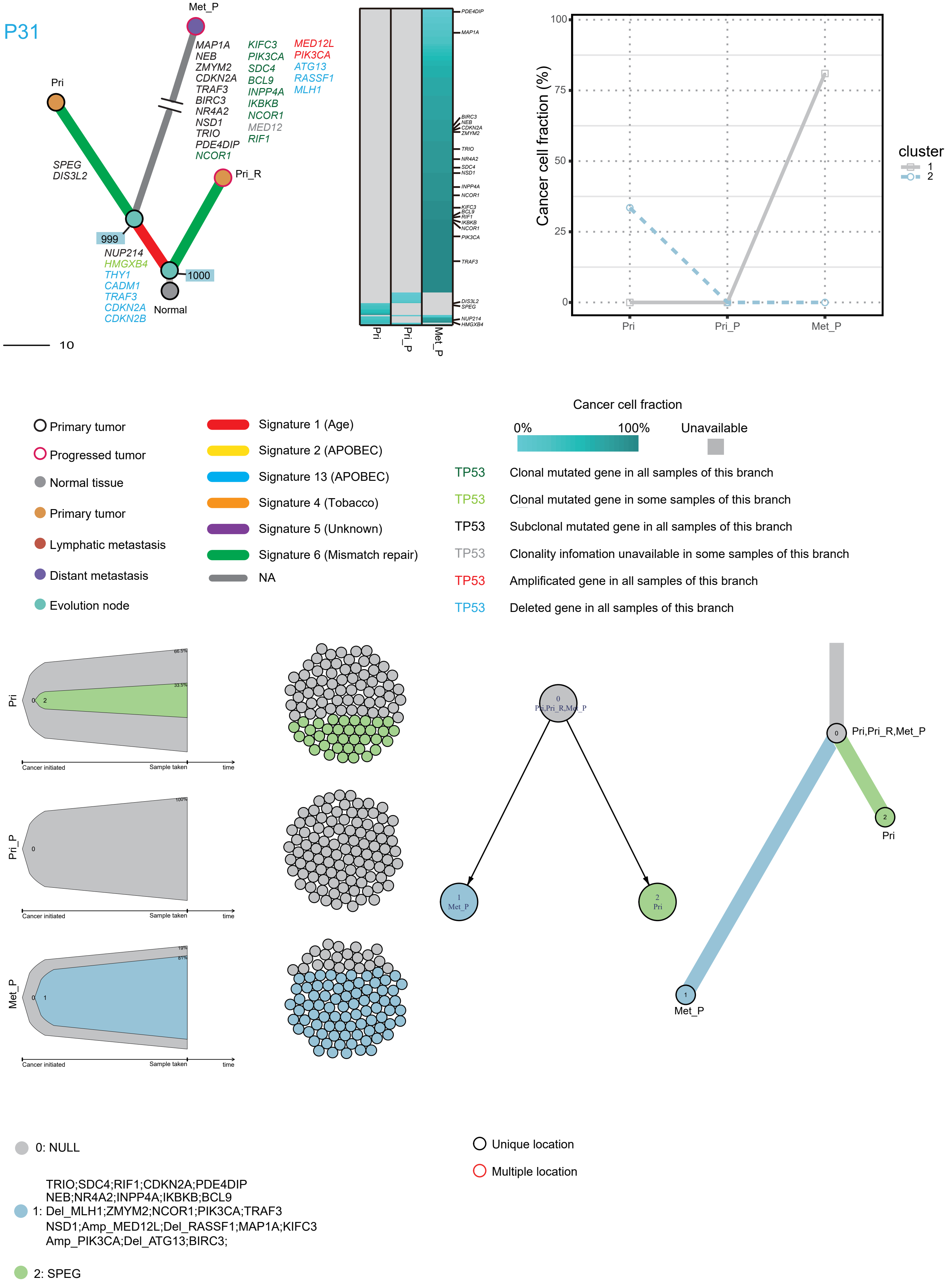
P31
Met_P
Pri
Pri_R
Normal
999
1000
10
MAP1A
NEB
ZMYM2
CDKN2A
TRAF3
BIRC3
NR4A2
NSD1
TRIO
PDE4DIP
NCOR1
KIFC3
PIK3CA
SDC4
BCL9
INPP4A
IKBKB
NCOR1
MED12
RIF1
MED12L
PIK3CA
ATG13
RASSF1
MLH1
SPEG
DIS3L2
NUP214
HMGXB4
THY1
CADM1
TRAF3
CDKN2A
CDKN2B
Pri
Pri_P
Met_P
Cancer cell fraction (%)
100
75
50
25
0
cluster
1
2
Primary tumor
Progressed tumor
Normal tissue
Primary tumor
Lymphatic metastasis
Distant metastasis
Evolution node
Signature 1 (Age)
Signature 2 (APOBEC)
Signature 13 (APOBEC)
Signature 4 (Tobacco)
Signature 5 (Unknown)
Signature 6 (Mismatch repair)
NA
Cancer cell fraction
0%
100%
Unavailable
TP53 Clonal mutated gene in all samples of this branch
TP53 Clonal mutated gene in some samples of this branch
TP53 Subclonal mutated gene in all samples of this branch
TP53 Clonality infomation unavailable in some samples of this branch
TP53 Amplificated gene in all samples of this branch
TP53 Deleted gene in all samples of this branch
Pri
Pri_P
Met_P
Cancer initiated
Sample taken
time
0
Pri,Pri_R,Met_P
1
Met_P
2
Pri
Pri,Pri_R,Met_P
Unique location
Multiple location
0: NULL
1: TRIO;SDC4;RIF1;CDKN2A;PDE4DIP
NEB;NR4A2;INPP4A;IKBKB;BCL9
Del_MLH1;ZMYM2;NCOR1;PIK3CA;TRAF3
NSD1;Amp_MED12L;Del_RASSF1;MAP1A;KIFC3
Amp_PIK3CA;Del_ATG13;BIRC3;
2: SPEG

af

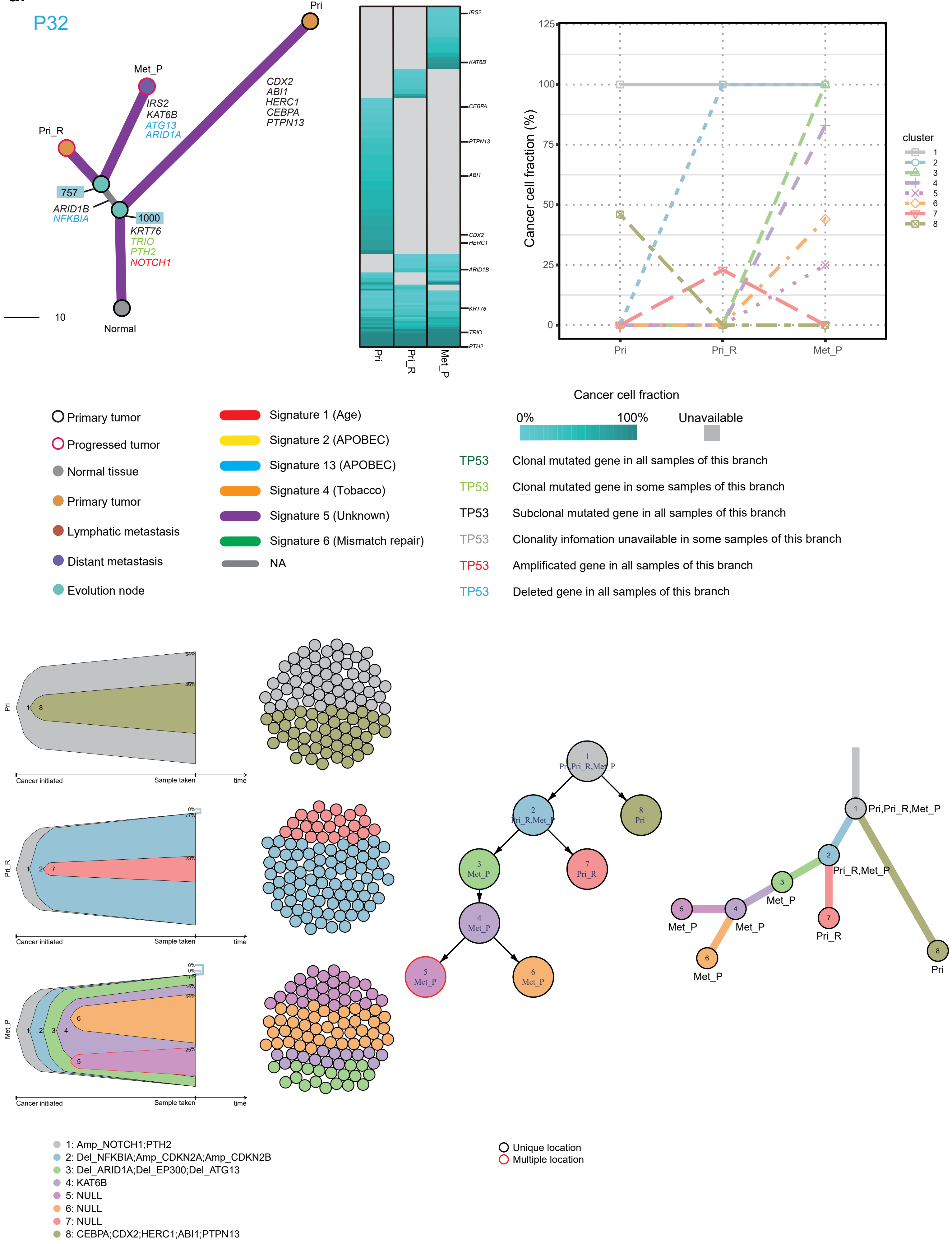

P32
Pri
Met_P
Pri_R
IRS2
KAT6B
ATG13
ARID1A
CDX2
ABI1
HERC1
CEBPA
PTPN13
757
ARID1B
NFKBIA
1000
KRT76
TRIO
PTH2
NOTCH1
10
Normal
IRS2
KAT6B
CEBPA
PTPN13
ABI1
CDX2
HERC1
ARID1B
KRT76
TRIO
PTH2
Pri
Pri_R
Met_P
Cancer cell fraction (%)
125
100
75
50
25
0
Pri
Pri_R
Met_P
cluster
1
2
3
4
5
6
7
8
Primary tumor
Progressed tumor
Normal tissue
Primary tumor
Lymphatic metastasis
Distant metastasis
Evolution node
Signature 1 (Age)
Signature 2 (APOBEC)
Signature 13 (APOBEC)
Signature 4 (Tobacco)
Signature 5 (Unknown)
Signature 6 (Mismatch repair)
NA
Cancer cell fraction
0%
100%
Unavailable
TP53 Clonal mutated gene in all samples of this branch
TP53 Clonal mutated gene in some samples of this branch
TP53 Subclonal mutated gene in all samples of this branch
TP53 Clonality infomation unavailable in some samples of this branch
TP53 Amplificated gene in all samples of this branch
TP53 Deleted gene in all samples of this branch
Pri
Cancer initiated
Sample taken
time
Pri_R
Cancer initiated
Sample taken
time
Met_P
Cancer initiated
Sample taken
time
1 Pri,Pri_R,Met_P
2 Pri_R,Met_P
8 Pri
3 Met_P
7 Pri_R
4 Met_P
5 Met_P
6 Met_P
Pri,Pri_R,Met_P
Pri_R,Met_P
Met_P
Met_P
Met_P
Met_P
Pri_R
Pri
1: Amp_NOTCH1;PTH2
2: Del_NFKBIA;Amp_CDKN2A;Amp_CDKN2B
3: Del_ARID1A;Del_EP300;Del_ATG13
4: KAT6B
5: NULL
6: NULL
7: NULL
8: CEBPA;CDX2;HERC1;ABI1;PTPN13
Unique location
Multiple location

ag

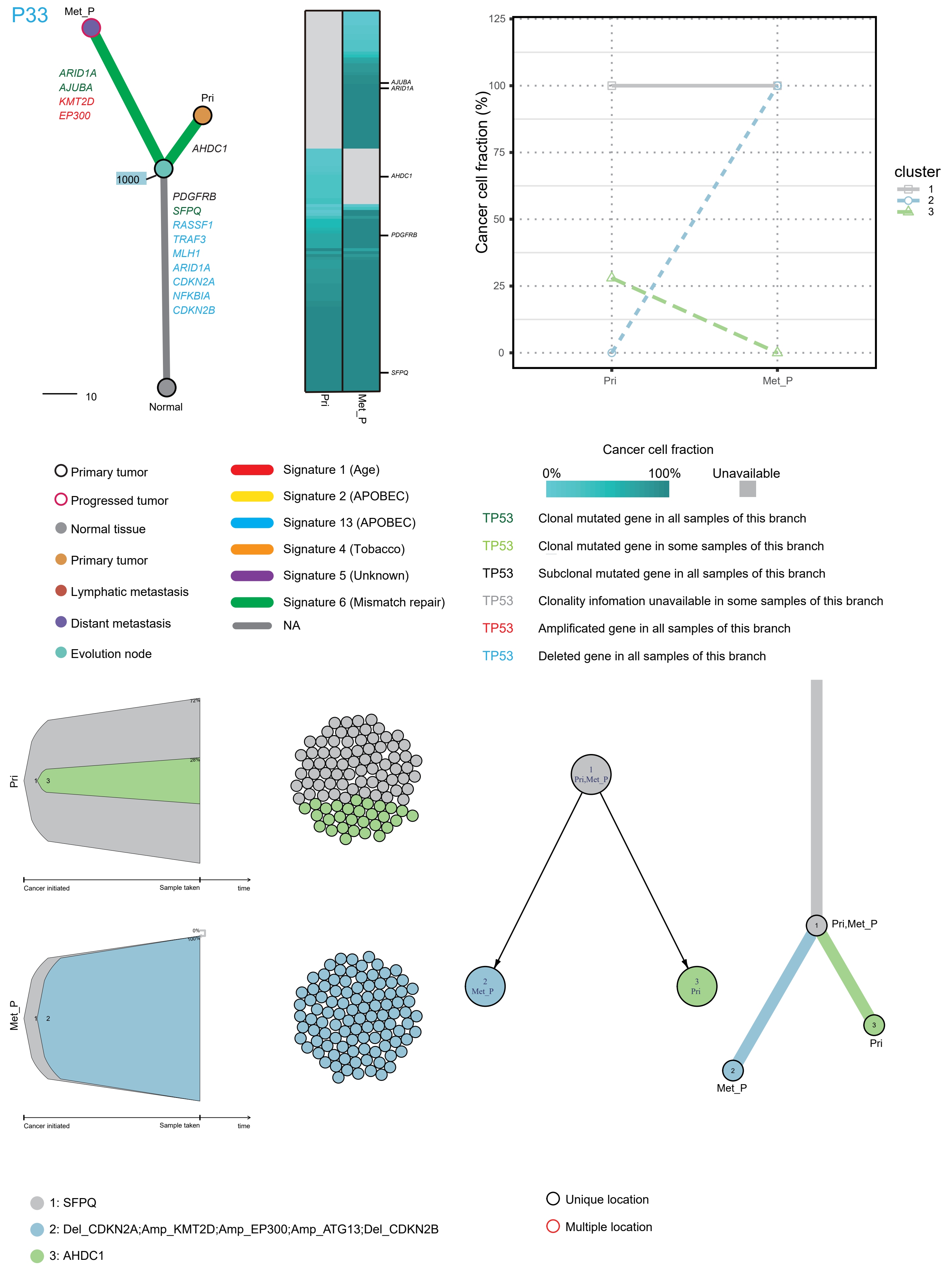

P33
Met_P
ARID1A
AJUBA
KMT2D
EP300
Pri
AHDC1
1000
PDGFRB
SFPQ
RASSF1
TRAF3
MLH1
ARID1A
CDKN2A
NFKBIA
CDKN2B
10
Normal
AJUBA
ARID1A
AHDC1
PDGFRB
SFPQ
Pri
Met_P
Cancer cell fraction (%)
125
100
75
50
25
0
Pri
Met_P
cluster
1
2
3
Primary tumor
Progressed tumor
Normal tissue
Primary tumor
Lymphatic metastasis
Distant metastasis
Evolution node
Signature 1 (Age)
Signature 2 (APOBEC)
Signature 13 (APOBEC)
Signature 4 (Tobacco)
Signature 5 (Unknown)
Signature 6 (Mismatch repair)
NA
Cancer cell fraction
0%
100%
Unavailable
TP53 Clonal mutated gene in all samples of this branch
TP53 Clonal mutated gene in some samples of this branch
TP53 Subclonal mutated gene in all samples of this branch
TP53 Clonality infomation unavailable in some samples of this branch
TP53 Amplificated gene in all samples of this branch
TP53 Deleted gene in all samples of this branch
Pri
72%
28%
1
3
Cancer initiated
Sample taken
time
Met_P
0%
100%
1
2
Cancer initiated
Sample taken
time
1
Pri,Met_P
2
Met_P
3
Pri
1
Pri,Met_P
2
Met_P
3
Pri
1: SFPQ
2: Del_CDKN2A;Amp_KMT2D;Amp_EP300;Amp_ATG13;Del_CDKN2B
3: AHDC1
Unique location
Multiple location

ah

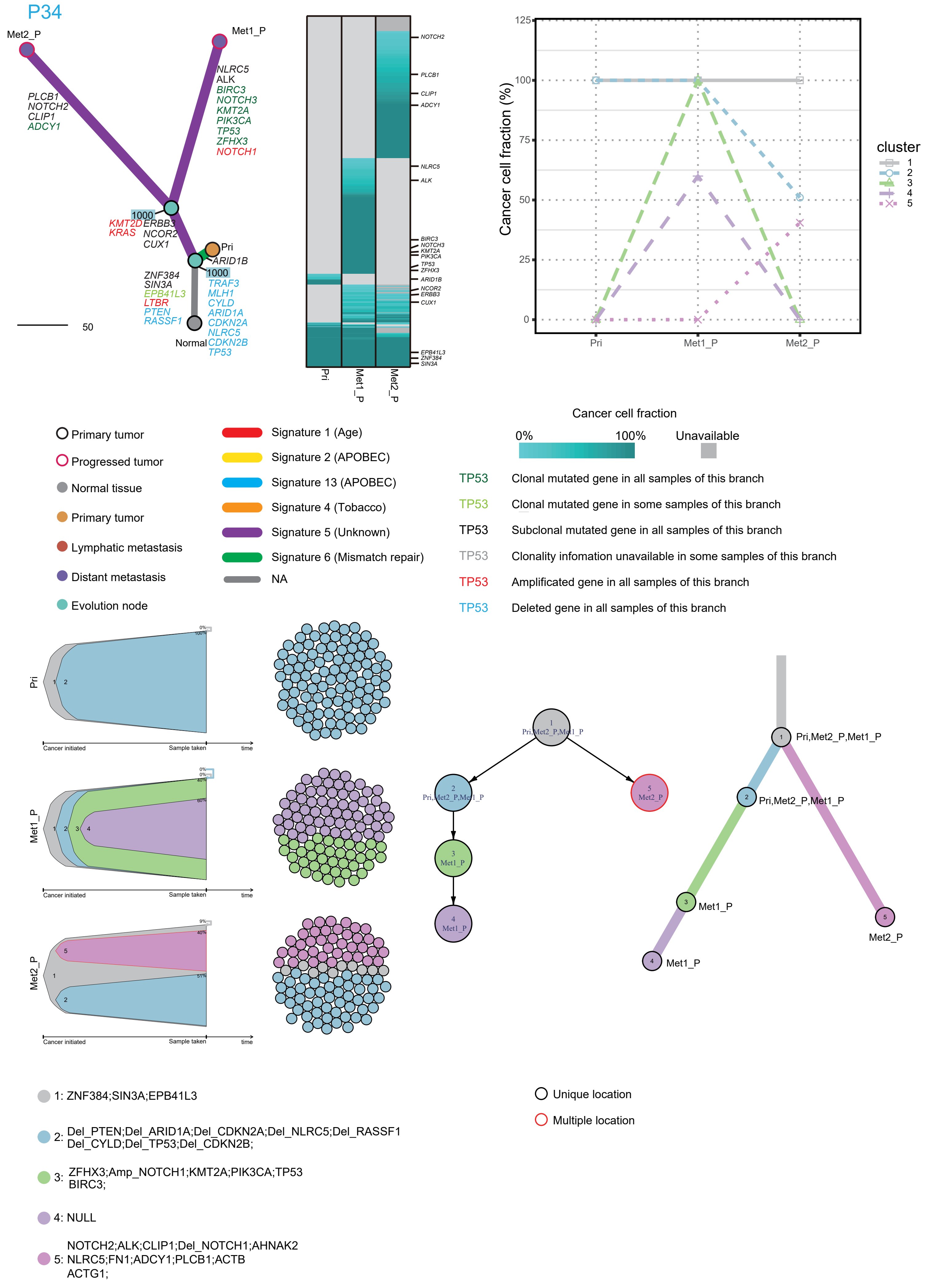
P34
Met2_P
Met1_P
PLCB1
NOTCH2
CLIP1
ADCY1
NLRC5
ALK
BIRC3
NOTCH3
KMT2A
PIK3CA
TP53
ZFHX3
NOTCH1
1000
KMT2D
KRAS
ERBB3
NCOR2
CUX1
Pri
ARID1B
ZNF384
SIN3A
EPB41L3
LTBR
PTEN
RASSF1
TRAF3
MLH1
CYLD
ARID1A
CDKN2A
NLRC5
CDKN2B
TP53
Normal
50
NOTCH2
PLCB1
CLIP1
ADCY1
NLRC5
ALK
BIRC3
NOTCH3
KMT2A
PIK3CA
TP53
ZFHX3
ARID1B
NCOR2
ERBB3
CUX1
EPB41L3
ZNF384
SIN3A
Pri
Met1_P
Met2_P
Cancer cell fraction (%)
125
100
75
50
25
0
Pri
Met1_P
Met2_P
cluster
1
2
3
4
5
Cancer cell fraction
0%
100%
Unavailable
Primary tumor
Progressed tumor
Normal tissue
Primary tumor
Lymphatic metastasis
Distant metastasis
Evolution node
Signature 1 (Age)
Signature 2 (APOBEC)
Signature 13 (APOBEC)
Signature 4 (Tobacco)
Signature 5 (Unknown)
Signature 6 (Mismatch repair)
NA
TP53 Clonal mutated gene in all samples of this branch
TP53 Clonal mutated gene in some samples of this branch
TP53 Subclonal mutated gene in all samples of this branch
TP53 Clonality infomation unavailable in some samples of this branch
TP53 Amplificated gene in all samples of this branch
TP53 Deleted gene in all samples of this branch
Pri
Cancer initiated
Sample taken
time
Met1_P
Met2_P
Pri,Met2_P,Met1_P
Met1_P
Met2_P
Unique location
Multiple location
1: ZNF384;SIN3A;EPB41L3
2: Del_PTEN;Del_ARID1A;Del_CDKN2A;Del_NLRC5;Del_RASSF1
Del_CYLD;Del_TP53;Del_CDKN2B;
3: ZFHX3;Amp_NOTCH1;KMT2A;PIK3CA;TP53
BIRC3;
4: NULL
5: NOTCH2;ALK;CLIP1;Del_NOTCH1;AHNAK2
NLRC5;FN1;ADCY1;PLCB1;ACTB
ACTG1;

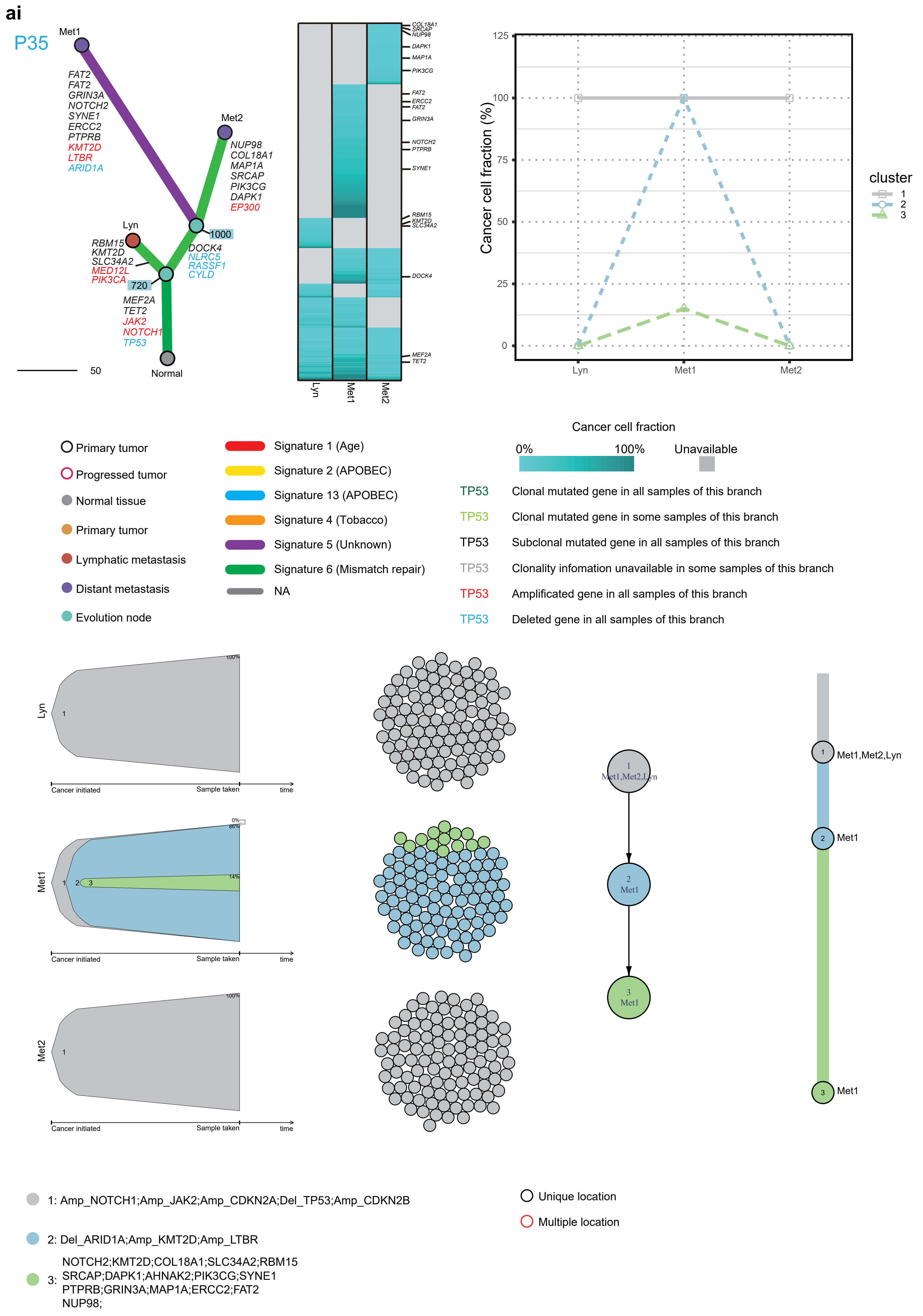

ai
P35
Met1
FAT2
FAT2
GRIN3A
NOTCH2
SYNE1
ERCC2
PTPRB
KMT2D
LTBR
ARID1A
Met2
NUP98
COL18A1
MAP1A
SRCAP
PIK3CG
DAPK1
EP300
1000
Lyn
RBM15
KMT2D
SLC34A2
MED12L
PIK3CA
DOCK4
NLRC5
RASSF1
CYLD
720
MEF2A
TET2
JAK2
NOTCH1
TP53
50
Normal
COL18A1
SRCAP
NUP98
DAPK1
MAP1A
PIK3CG
FAT2
ERCC2
FAT2
GRIN3A
NOTCH2
PTPRB
SYNE1
RBM15
KMT2D
SLC34A2
DOCK4
MEF2A
TET2
Lyn
Met1
Met2
Cancer cell fraction (%)
125
100
75
50
25
0
Lyn
Met1
Met2
cluster
1
2
3
Primary tumor
Progressed tumor
Normal tissue
Primary tumor
Lymphatic metastasis
Distant metastasis
Evolution node
Signature 1 (Age)
Signature 2 (APOBEC)
Signature 13 (APOBEC)
Signature 4 (Tobacco)
Signature 5 (Unknown)
Signature 6 (Mismatch repair)
NA
Cancer cell fraction
0%
100%
Unavailable
TP53 Clonal mutated gene in all samples of this branch
TP53 Clonal mutated gene in some samples of this branch
TP53 Subclonal mutated gene in all samples of this branch
TP53 Clonality infomation unavailable in some samples of this branch
TP53 Amplificated gene in all samples of this branch
TP53 Deleted gene in all samples of this branch
Lyn
1
100%
Cancer initiated
Sample taken
time
Met1
1
2
3
0%
86%
14%
Cancer initiated
Sample taken
time
Met2
1
100%
Cancer initiated
Sample taken
time
1
Met1,Met2,Lyn
2
Met1
3
Met1
1 Met1,Met2,Lyn
2 Met1
3 Met1
1: Amp_NOTCH1;Amp_JAK2;Amp_CDKN2A;Del_TP53;Amp_CDKN2B
2: Del_ARID1A;Amp_KMT2D;Amp_LTBR
3: NOTCH2;KMT2D;COL18A1;SLC34A2;RBM15
SRCAP;DAPK1;AHNAK2;PIK3CG;SYNE1
PTPRB;GRIN3A;MAP1A;ERCC2;FAT2
NUP98;
Unique location
Multiple location

Supplementary Fig. 11. Evolution analysis of each patient.

Evolution analysis for 35 patients. A phylogenetic tree was constructed for each patient (top left figure for each patient). For each branch, key variants are highlighted in the tree, including clonal mutated driver genes in all samples (dark green), clonal mutated driver genes in some samples (light green), subclonal mutated driver genes in all samples (black), and mutated driver genes with unclear clonality (grey) as well as key amplified or deleted driver genes (red and blue). Bootstrap values are marked on each divergence node, and dominant signatures (contribution > 0.5) of each branch are mapped in different colours (except the branches that contain less than 10 mutations or had no dominant signature detected). The top middle figures for each patient show the fraction of tumour cells with shared and private mutations for each tumour sample, and key mutations are also marked. The top right figures show the CCF flow of each mutation cluster (subclones) according to each sample. The bottom left figures show the subclone-based evolution architecture of each sample and their sampling transect, which reflect the fractions of each subclone at the time of sampling. The bottom right two figures show the evolution architecture of subclones in node-based and branch-based trees. The black boundary subclones in the node-based tree indicate that these subclones are located in a unique place of the architecture, whereas the red subclones indicate that the subclones are so small that they could be placed in multiple locations without penalty (see Method). Driver variants are also listed for each subclone.

SUPPLEMENTARY TABLES

Supplementary Table 1. Clinical data of the sequenced NPC cohort.

Type	Group	Frequency	Percent
Age			
	Median (yrs)		42
Sex			
	Female	4	9.09%
	Male	40	90.91%
Smoking			
	No	27	61.36%
	Yes	17	38.64%
T stage			
	1	2	4.55%
	2	4	9.09%
	3	17	38.64%
	4	21	47.73%
N stage			
	1	7	15.91%
	2	14	31.82%
	3	23	52.27%
M stage			
	0	9	20.45%
	1	35	79.55%
Disease stage			
	II	1	2.27%
	III	2	4.55%
	IVa	5	13.63%
	IVb	35	79.55%
Progression status			
	no	10	22.73%
	yes	34	77.27%
PFS			
	Median (months)		12

Supplementary Table 2. Single cell RNA sequencing information.

Patient	Sample	UMI (mean)	Genes (mean)	UMI (std)	Genes (std)	Cells
P14	Pri	4763.9	1203.1	5931.7	847.1	4921
	LLN	2983.2	938.9	3615.9	615.7	1954
	RLN	3217	962.7	2561.3	466.1	3074
	Liver	7335.2	1473.2	11502.2	1218.9	3156
	Bone	4203.6	1019.9	6380	837.8	478
	ILN	2694.8	811.1	2533.2	494.3	6102
P15	Pri	4428.3	1131.8	5811	895.3	4868
	LLN	3665.2	966.3	2934.5	468.3	6809
	RLN	3877.3	1081.1	3246.5	484.8	7172
	LALN	3559.5	981.4	2579.2	485.3	7886
	RALN	4952.8	1195.1	5989.2	807.7	7493

Supplementary Table 3. Key image characteristics of the training and validation datasets.

Type	Lymphatic median(range)	Hematogenous median(range)	P value*
**Training dataset (n = 13)**			
Axial maximum diameter of tumor (mm)	19.4 (14.9-46.4)	48.9 (28-53.2)	0.006216
Coronal maximum diameter (mm)	22.75 (16.1-47)	53.7 (38-64.8)	0.003108
Sagittal maximum diameter (mm)	18.7 (10.7-64.7)	48.7 (35.2-74.1)	0.010878
Number of lower cervical lymph nodes	14.5 (5-22)	5 (0-12)	0.0225166
Number of organ metastasis	2 (0-9)	6 (0-52)	0.2370052
Number of bone metastasis	18 (0-27)	0 (0-7)	0.0594305
**Metastatic NPC cohort (n = 104)**			
Axial maximum diameter of tumor(mm)	34.35 (14.1-59.0)	39.55 (29.9-66.1)	0.03881
Coronal maximum diameter (mm)	32.15 (13.5-67.5)	38.15 (25.7-69.4)	0.012888
Sagittal maximum diameter (mm)	27.7 (4.12-63.9)	35.3 (20.1-62.1)	0.039573
Number of lower cervical lymph nodes	3 (0-33)	2.5 (0-6)	0.09207
Number of organ metastasis	1 (1-4)	1 (0-3)	0.950072
Number of bone metastasis	3 (0-5)	1.5 (0-5)	0.513893
**M0 stage NPC cohort (n = 201)**			
Axial maximum diameter of tumor(mm)	18 (5-48)	28 (8-71)	1.657E-09
Coronal maximum diameter (mm)	19 (5-48)	28 (7-61)	2.158E-09
Sagittal maximum diameter (mm)	19 (5-70)	25.5 (8-59)	4.267E-11
Number of lower cervical lymph nodes	0 (0-6)	0 (0-7)	0.0374658
**Immunotherapy cohort (n = 66)**			
Axial maximum diameter of tumor(mm)	31 (9.5-40)	37.15 (19.9-58.5)	0.0123199
Coronal maximum diameter (mm)	34.9 (15.9-46.2)	36.2 (13.1-60.3)	0.3505177
Sagittal maximum diameter (mm)	28.5 (13.1-44.4)	35.3 (13.7-60.9)	0.0390839
Number of lower cervical lymph nodes	2 (0-8)	0 (0-10)	0.051894

*P value was calculated using Wilcoxon signed-rank test (two-sided).

Supplementary Table 4. Clinical characteristics of the de novo metastatic NPC cohort used for the validation of the radiomics prediction model (N = 104 individuals).

Type	Group	Frequency	Percent
Metastatic route			
	Lymphatic route	78	75.0%
	Hematogenous route	26	25.0%
Local-regional radiotherapy			
	Yes	50	48.1%
	No	54	51.9%
Age, years			
	Media (IQR)		37.0-52.0
Gender			
	Male	87	83.7%
	Female	17	16.3%
Smoking			
	Yes	32	30.8%
	No	72	69.2%
KPS score			
	Median (IQR)		90-90
Pathology (WHO)			
	I/II	5	4.8%
	III	99	95.2%
T stage			
	1-2	12	11.5%
	3-4	92	88.5%
N stage			
	0-1	22	21.2%
	2-3	82	78.8%
Bone metastases			
	No	27	26.0%
	Yes	77	74.0%
Liver metastases			
	No	73	70.2%
	Yes	31	29.8%
Lung metastases			
	No	74	71.10%
	Yes	30	28.90%
Number of metastatic lesions			
	1-2	31	30.00%
	≥3	73	70.00%

Supplementary Table 5. Comparison of clinicopathologic features of the training and de novo metastatic NPC cohorts.

Category and variable	training cohort (n = 13)	validation cohort (n=104)	P value*
Age	46.0 (38.5-51.0)	47.0 (36.5-53.0)	0.423[a]
Sex			0.455
Female	1(7.7%)	15(14.4%)	
Male	12(92.3%)	89(85.6%)	
Number of metastatic sites			0.327
Single	5(38.5%)	28(26.9%)	
Multiple	8(61.5%)	76(73.1%)	
Liver involvement			0.053
No	6(46.2%)	72(69.2%)	
Yes	7(53.8%)	32(30.8%)	
Treatment			0.257
CT+RT	4(30.8%)	46(44.2%)	
CT	9(69.2%)	58(55.8%)	
Group			0.388
Lymphatic	78(75.0%)	8(61.5%)	
Hematogenous	26(25.0%)	5(38.5%)	

*P value was calculated using Chi-square test (two-sided). [a] P value was calculated using two-sided t test. CT: Chemotherapy; RT: Radiotherapy.

Supplementary Table 6. Clinical characteristics of the M0-stage nasopharyngeal carcinoma cohort used for the validation of the radiomics prediction model (N=201 individuals).

Type	Group	Frequency	Percent
Metastatic route			
	Lymphatic route	113	56.22%
	Hematogenous route	88	43.78%
Age			
	Median (IQR)	45	38-53
KPS			
	Median (IQR)	90	90-90
Gender			
	Male	144	71.64%
	Female	57	28.36%
Pathology (WHO)			
	I/II	8	3.98%
	III	193	96.02%
T stage			
	1-2	92	45.77%
	3-4	109	54.23%
N stage			
	0-1	147	73.13%
	2-3	54	26.87%
Chemotherapy			
	No	18	8.96%
	Yes	183	91.04%

Supplementary Table 7. Clinical characteristics of the immunotherapy NPC cohort (N = 66 individuals).

Type	Lymphatic route (n=52)	Hematogenous route (n=14)	P value*
Stage			0.766
Primary metastases	1	0	
Recurrence with distant metastases	43	11	
Local regional-recurrence	8	3	
Age, years			0.899[a]
Median (IQR)	47 (35.0-56.0)	44 (37.0-53.0)	
Gender			0.73
Male	40	10	
Female	12	4	
Smoking			0.971
Yes	22	6	
No	30	8	
KPS score			1[a]
Median (IQR)	90 (90-90)	90 (90-90)	
Pathology (WHO)			1
I/II	2	0	
III	50	14	

*P values were calculated by Chi-square test. [a] P value was calculated using two-sided t test.

Supplementary Table 8. Multivariate analysis assessing the risk factors for PFS in the de novo metastatic NPC cohort.

Category and variable	PFS<br>HR (95% CI)	PFS<br>P value*
Age		
	0.978 (0.955-1.003)	0.082
Sex		
Female	Ref	
Male	0.894(0.467-1.714)	0.737
Number of metastatic sites		
Single	Ref	
Multiple	1.103(0.64-1.898)	0.725
Liver involvement		
No	Ref	
Yes	0.986(0.569-1.709)	0.96
Treatment		
CT	Ref	
CT+RT	2.65(1.612-4.356)	<0.001
Group		
Lymphatic	Ref	
Hematogenous	0.397(0.191-0.825)	0.013

*P values were calculated using a Cox proportional hazards model. CT: Chemotherapy; RT: Radiotherapy.
