# Supplementary material for: Evolutionary route of nasopharyngeal carcinoma metastasis and its clinical significance
Source: Nat Commun. 2023 Feb 4;14:610. doi: 10.1038/s41467-023-35995-2 (PMC9899247; doi:10.1038/s41467-023-35995-2)
Supplement: Supplementary file 2 — Description to Additional Supplementary Files [file 41467_2023_35995_MOESM2_ESM.pdf]

SUPPLEMENTARY DATA

Supplementary Data 1. Genome sequencing (WGS/WES) information.

Supplementary Data 2. Transcriptome sequencing information.

Supplementary Data 3. Somatic mutations of all samples.

Supplementary Data 4. Mutation validation by Sanger sequencing.

Supplementary Data 5. Copy number variant segmentation file for all samples.

Supplementary Data 6. All 192 radiomics features of the training dataset.

Supplementary Data 7. Custom codes.
